# Parallel Evolution of Complex Centipede Venoms Revealed by Comparative Proteotranscriptomic Analyses

**DOI:** 10.1093/molbev/msz181

**Published:** 2019-08-08

**Authors:** Ronald A Jenner, Bjoern M von Reumont, Lahcen I Campbell, Eivind A B Undheim

**Affiliations:** 1 Department of Life Sciences, Natural History Museum, London, United Kingdom; 2 LOEWE Centre for Translational Biodiversity Genomics (LOEWE-TBG), Frankfurt, Germany; 3 Institute for Insect Biotechnology, Justus-Liebig University Giessen, Giessen, Germany; 4 Department of Bioresources, Fraunhofer Institute for Molecular Biology and Applied Ecology, Animal Venomics, Giessen, Germany; 5 The European Molecular Biology Laboratory, The European Bioinformatics Institute, Hinxton, United Kingdom; 6 Centre for Advanced Imaging, University of Queensland, St Lucia, Australia; 7 Institute for Molecular Bioscience, University of Queensland, St Lucia, Australia; 8 Centre for Ecology and Evolutionary Synthesis, Department of Bioscience, University of Oslo, Oslo, Norway

**Keywords:** centipedes, proteomics, transcriptomics, venom

## Abstract

Centipedes are among the most ancient groups of venomous predatory arthropods. Extant species belong to five orders, but our understanding of the composition and evolution of centipede venoms is based almost exclusively on one order, Scolopendromorpha. To gain a broader and less biased understanding we performed a comparative proteotranscriptomic analysis of centipede venoms from all five orders, including the first venom profiles for the orders Lithobiomorpha, Craterostigmomorpha, and Geophilomorpha. Our results reveal an astonishing structural diversity of venom components, with 93 phylogenetically distinct protein and peptide families. Proteomically-annotated gene trees of these putative toxin families show that centipede venom composition is highly dynamic across macroevolutionary timescales, with numerous gene duplications as well as functional recruitments and losses of toxin gene families. Strikingly, not a single family is found in the venoms of representatives of all five orders, with 67 families being unique for single orders. Ancestral state reconstructions reveal that centipede venom originated as a simple cocktail comprising just four toxin families, with very little compositional evolution happening during the approximately 50 My before the living orders had diverged. Venom complexity then increased in parallel within the orders, with scolopendromorphs evolving particularly complex venoms. Our results show that even venoms composed of toxins evolving under the strong constraint of negative selection can have striking evolutionary plasticity on the compositional level. We show that the functional recruitments and losses of toxin families that shape centipede venom arsenals are not concentrated early in their evolutionary history, but happen frequently throughout.

## Introduction

Venom is one of nature’s most frequently evolved adaptations. A conservative estimate suggests it has evolved more than 80 times in the animal kingdom to play important roles in predation, defense, blood feeding, and other functions (Fry et al. 2009; Casewell et al. 2013; Jenner and Undheim 2017). Venoms are typically complex cocktails of bioactive molecules, commonly referred to as toxins that disrupt normal physiological functioning of envenomated victims. Most of these toxins are proteins and peptides, which are thought to have mainly evolved through the functional recruitment of physiological components into venom, where they can evolve new roles and functions as toxins (Casewell et al. 2013). This makes venoms well-suited models for understanding the evolution of novel adaptive traits. Various mechanisms can be involved in toxin recruitment and evolution, including recruitment of single-copy nontoxin genes, changes in the location and level of gene expression, gene duplication followed by positive selection to facilitate functional diversification, gene or domain duplication combined with concerted evolution to increase effective expression levels, negative selection to conserve the function of ecologically important toxins, and functional loss of toxins through transcriptional and translational downregulation, which can lead to pseudogenization or complete loss of toxin genes (Moran et al. 2008; Fry et al. 2009; Casewell et al. 2013; Hargreaves et al. 2014; Reyes-Velasco et al. 2015; Sunagar and Moran 2015; Madio et al. 2018; Laxme et al. 2019). These mechanisms of toxin recruitment, maintenance, diversification, and loss also drive the macroevolution of venom on a compositional level. However, their relative contribution to the evolutionary dynamics of venom composition on the level of gene families remains poorly understood.

**Fig. 1.**
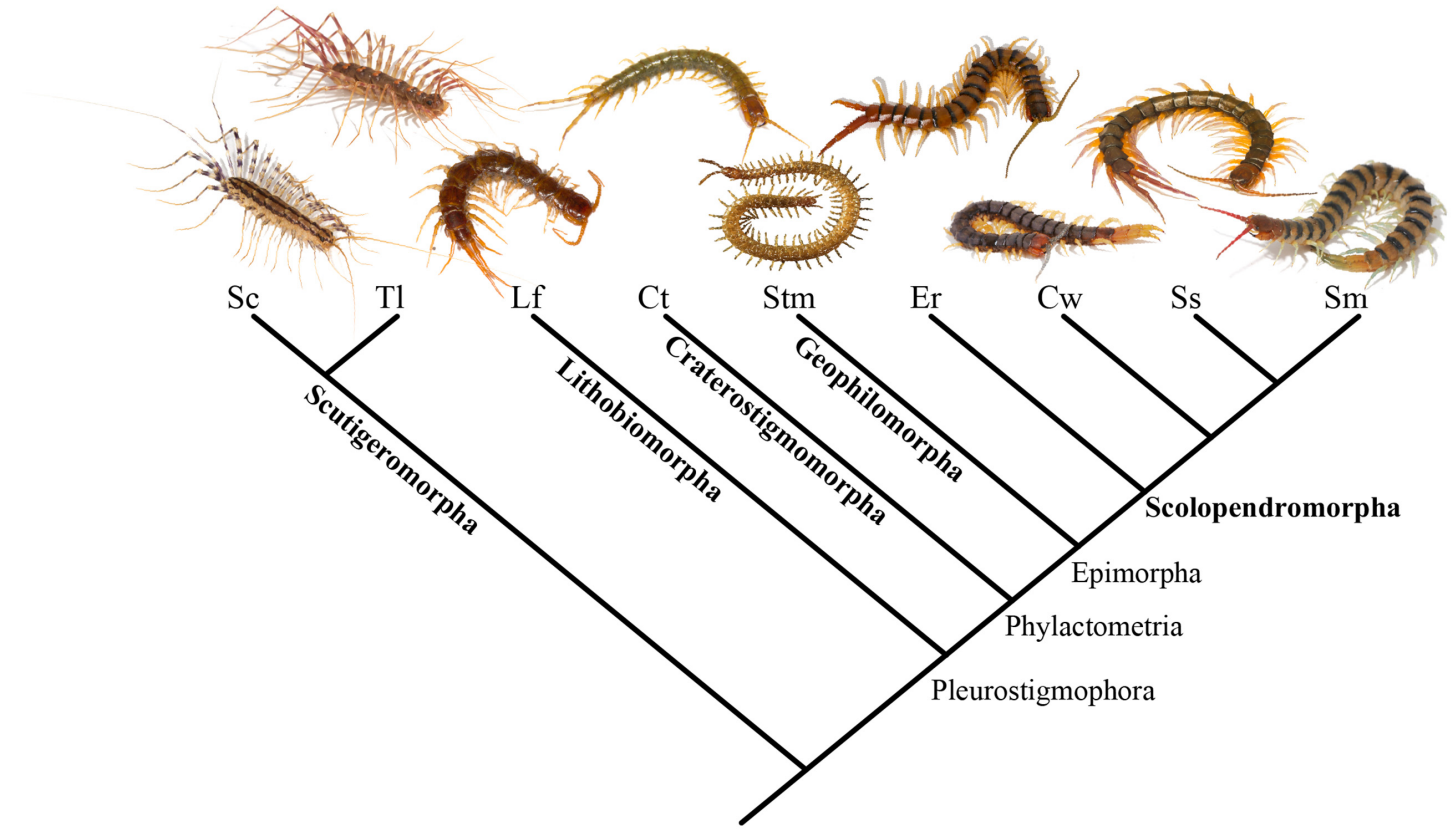
Phylogenetic relationships of the species analyzed in this study. Names of the centipede orders are shown in bold and higher-level clades are indicated at the bases of their respective lineages. Abbreviations of species names are as follows: Sc*, Scutigera coleoptrata*; Tl*, Thereuopoda longicornis*; Lf*, Lithobius forficatus*; Ct*, Craterostigmus tasmanianus*; Stm*, Strigamia maritima*; Er*, Ethmostigmus rubripes*; Cw*, Cormocephalus westwoodi*; Ss*, Scolopendra subspinipes*; Sm*, Scolopendra morsitans*. See Materials and Methods for references supporting the tree topology.

This study focuses on the venoms of centipedes (Myriapoda: Chilopoda), an ancient group of predatory arthropods with more than 3,100 described species. Centipedes are among the oldest known terrestrial venomous lineages, with a fossil record going back at least 420 My. A diagnostic trait of centipedes is the modification of their first walking appendages into venom-delivering claw-like structures called forcipules. Because all known extant centipede species have a forcipular venom system, and forcipules are known from the oldest known centipede fossils, venom is assumed to have evolved once in the stem lineage of centipedes. The living species belong to five lineages that are classified as orders (fig. 1): Scutigeromorpha (house centipedes), Lithobiomorpha (stone centipedes), Geophilomorpha (earth centipedes with long, thin bodies), Craterostigmomorpha (two species from New Zealand and Tasmania), and Scolopendromorpha (the most familiar centipedes, including large tropical species). Although these orders diverged during the Paleozoic (Fernández et al. 2016), members of all orders have generally very similar morphologies and they use their venoms for similar purposes, namely predation on predominantly arthropod prey and defense against a variety of invertebrate and vertebrate predators. Centipedes thus offer a rare opportunity to examine the evolution of a homologous venom system across species that have retained relatively similar body plans and life histories for several hundreds of millions of years.

The empirical foundation of our understanding of centipede venoms is very narrow. With the sole exception of Undheim, Jones, et al. (2014), who profiled the composition of the first scutigeromorph venom, investigations of centipede venom composition are restricted to scolopendromorphs. Combined proteomic and transcriptomic (proteotranscriptomic) venom profiles are available for only six scolopendromorph species in the family Scolopendridae, four of which are from the genus *Scolopendra* (*Scolopendra morsitans*, *Scolopendra subspinipes*, *Scolopendra viridis*, and *Scolopendra dehaani*) (Liu et al. 2012; Yang et al. 2012; Gonzalez-Morales et al. 2014; Undheim, Jones, et al. 2014; Undheim, Fry, et al. 2015; Rong et al. 2015; Smith and Undheim 2018; Ward and Rokyta 2018). This taxonomic bias toward scolopendromorphs is equally conspicuous in the recent surge of papers exploring the promise of centipede venoms for the discovery and development of new pharmaceuticals (Yang et al. 2013; Chen et al. 2014; Hakim et al. 2015; Undheim et al. 2016; Lee et al. 2017; Wang et al. 2017).

Our current understanding of centipede venom evolution is based on four comparative studies (Undheim, Jones, et al. 2014; Undheim, Sunagar, et al. 2014; Undheim, Grimm, et al. 2015; Smith and Undheim 2018). These have revealed that centipede venoms are complex cocktails, encompassing more than 60 phylogenetically distinct protein and peptide families, including enzymes, β-pore-forming toxins (β-PFTxs), protease inhibitors, a great diversity of cysteine-rich peptides, as well as completely uncharacterized proteins (Undheim, Fry, et al. 2015). Although the majority of these components remain to be functionally characterized, for brevity we will refer to them as toxins and toxin families throughout the paper. Interestingly, although there is variation in venom composition between, as well as within, scolopendromorph species (Smith and Undheim 2018; Ward and Rokyta 2018), the most striking differences are observed between the venoms of scolopendromorphs and the only nonscolopendromorph studied to date, the scutigeromorph *Thereuopoda longicornis* (Undheim, Jones, et al. 2014). Scutigeromorph venom is less complex, and a larger proportion of it is made up of higher molecular weight proteins, with putatively cytolytic β-PFTxs being especially abundant. In contrast, scolopendromorph venoms contain a much greater diversity of cysteine-rich peptides, which are thought to be predominantly neurotoxic. Fascinatingly, the relative complexity of scutigeromorph and scolopendromorph venoms mirrors the complexity of their venom glands, with the more complex glands of scolopendromorphs producing more complex venoms (Undheim, Hamilton, et al. 2015). Throughout this paper we define venom complexity as the number of different toxin families found in venom, which agrees with commonly accepted measures of organismal complexity in terms of the number of different part types on each level of organization (Mcshea 2016). These striking differences between scutigeromorph and scolopendromorph venoms suggest that they may rely on different prey envenomation strategies, with scutigeromorphs chiefly relying on cytolytic β-PFTxs, and scolopendromorphs on neurotoxic peptides.

A comparison of scutigeromorph and scolopendromorph venoms allowed the first reconstruction of the ancestral venom protein arsenal of centipedes as a cocktail comprising a dozen protein and peptide families, including proteases, β-PFTx, CAP (*c*ysteine-rich secretory proteins, *a*ntigen 5 and *p*athogenesis-related) proteins, and cysteine-rich peptides (Undheim, Jones, et al. 2014; Undheim, Fry, et al. 2015). However, this ancestral reconstruction was very tentative because it was based on data from only two of the five centipede lineages. Interestingly, the phylogenetic distribution of venom components suggested that venom complexity increased in parallel in the scutigeromorph and scolopendromorph lineages since they diverged from the last common centipede ancestor (Undheim, Jones, et al. 2014). Furthermore, phylogenetic analyses provide evidence for substantial lineage-specific radiations for several peptide and protein families, such as β-PFTx, CAP proteins, and cysteine-rich peptides (Undheim, Jones, et al. 2014). Nevertheless, Sunagar and Moran (2015) concluded that centipede venom proteins have accumulated surprisingly little sequence variation despite their long evolutionary history, and have evolved slowly, under the strong constraint of purifying selection.

To gain a more balanced and a more detailed understanding of the composition and evolution of centipede venoms, more data are needed for nonscolopendromorph venoms. In this study, we present the first comparative proteotranscriptomic analysis of centipede venom composition with representatives of all five extant centipede lineages, including the first venom profiles for the orders Lithobiomorpha, Craterostigmomorpha, and Geophilomorpha. Our analyses provide the first overall picture of centipede venom composition, and reveal an astounding structural diversity of venom components, with over 90 phylogenetically distinct putative toxin families. Strikingly, there is no such thing as a typical centipede venom—not a single toxin family is found in the venom proteomes of all species or even in representatives of all five orders, with more than two thirds of protein families being restricted to the venoms of one of the orders. Phylogenetic analyses of these toxin families and reconstruction of their evolutionary histories across the species phylogeny reveal that centipede venom composition is highly dynamic over macroevolutionary timescales, with gene duplications contributing to toxin diversity, and numerous functional recruitments and losses of toxin gene families shaping the venom arsenals. Our data suggest that the ancestral centipede venom cocktail was considerably simpler than hitherto thought, and that centipede venom remained simple until after the major lineages had diverged from each other. Substantial increases in venom complexity then evolved in parallel within each of the orders. This dynamic picture of lineage-specific compositional evolution is in marked contrast to the previous finding that centipede venom toxins evolve slowly under the strong constraint of negative selection. Our results highlight the importance of frequent functional recruitments and losses of toxin families in shaping centipede venom arsenals throughout their evolutionary history.

## Results and Discussion

### Transcriptomic and Proteomic Venom Profiles of Centipedes

**Fig. 2.**
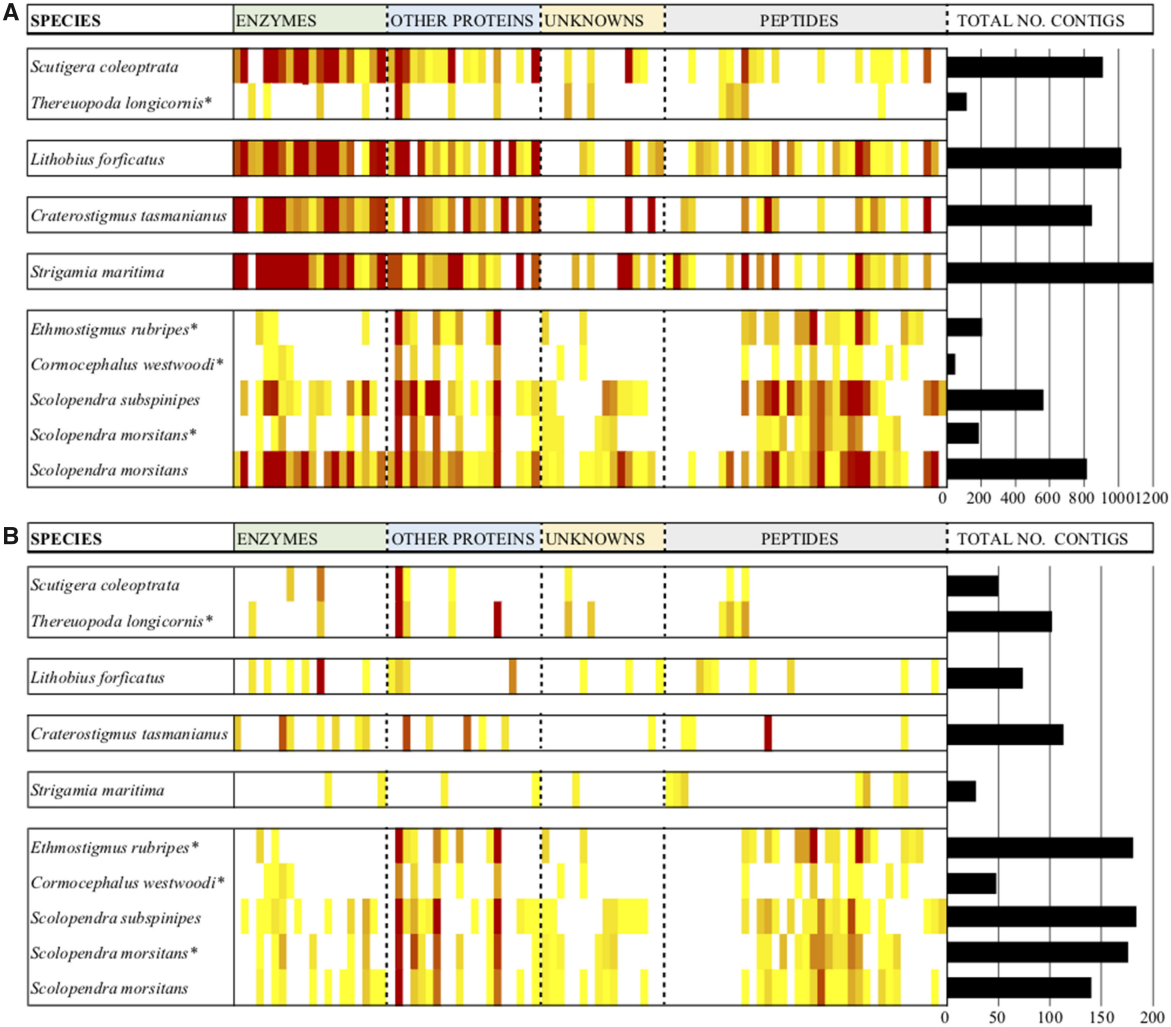
General composition of centipede venoms. The distribution of unique contigs encoding venom proteins and peptides in each family (columns) identified by (*A*) transcriptomics alone or (*B*) combined transcriptomics and venom proteomics are shown as heatmaps, whereas the total number of unique contigs identified in each species are shown as horizontal bars. The species are boxed according to their order (see fig. 1). The colors in the heatmap range from light yellow (indicating a single contig) to dark red (indicating a number within the 95th percentile, with maxima in the transcriptome [*A*] and proteome [*B*] data sets being 206 and 58 contigs, respectively). Asterisks mark species that were part of the study of Undheim, Jones, et al. (2014), with transcriptomes sequenced using Roche 454 technology. The other species were sequenced with Illumina technology. The full table used for generating the heatmap is provided as supplementary material S3 (Supplementary Material online).

To identify and classify venom components with putative toxic function, and to minimize toxin annotation error rates (Smith and Undheim 2018), we used a combined proteotranscriptomic and phylogenetic approach to analyze the venom composition of a set of species from the five order-level lineages (fig. 1). We found a total of 1,096 contigs encoding amino acid sequences that were identified in the venom proteomes. BLAST search of these against the respective transcriptomes of each species identified an additional 4,814 unique contigs encoding toxin-like amino acid sequences that were not identified in any venom proteome. The resulting total of 5,910 contigs were classified into 93 phylogenetically distinct toxin families according to the nomenclature established by Undheim, Jones, et al. (2014), each of which had at least one representative identified in the venom proteome of at least one species (fig. 2, supplementary material S1, Supplementary Material online). Among these were 14 putative toxin families that had not previously been described from centipede venom, including five protein families, namely acid phosphatase (*Craterostigmus tasmanianus*), phosphodiesterase (*S. morsitans*), calycin/lipocalin (*C. tasmanianus*), IgE Epididymal secretory protein-like (ESP-like; *C. tasmanianus*), and Pesticidal Crystal Protein Domain-containing Protein-like proteins (PCPDP-like; *Lithobius forficatus*). The remaining nine toxin families represent novel proteins and peptides, including two protein families with no similarity to any characterized protein or domain (Uncharacterized families 16 and 17 from *C. tasmanianus* and *L. forficatus*, respectively), and seven new venom peptide families with no recognizable similarity to any described peptide or peptide fold. Of these new peptide families, three were found only in the venom of *L. forficatus* (Lithotoxin, or LTHTX, 1–3), one was found in the venoms of *C. tasmanianus* and *Strigamia maritima* (Chilotoxin, or CHLTX01), and two were found only in the venom of *Stm. maritima* (Geotoxin, or GEOTX, 1 and 2).

**Fig. 3.**
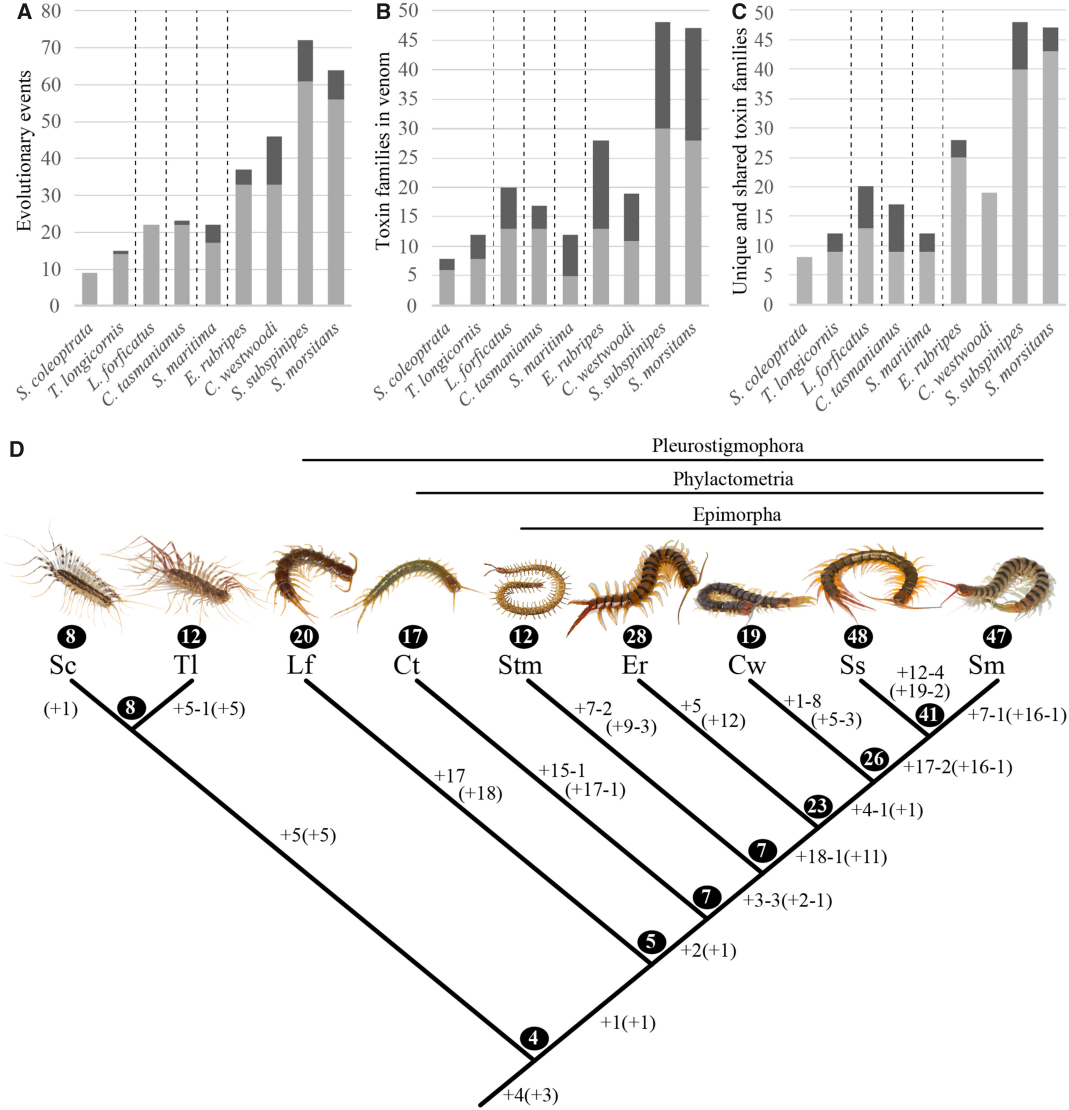
Comparison of evolutionary dynamics of venom composition between lineages. For each species, the graphs display the ACCTRAN estimations of (*A*) the total number of evolutionary events in the venom proteome since the last common centipede ancestor and how many of these are functional recruitments (light gray) or losses (dark gray), (*B*) the number of toxin families identified in the venom proteome and how many of these are proteins (light gray) or peptides (dark gray), and (*C*) the number of toxin families present in the venom proteome and how many of these are shared with at least one other species (light gray) or are unique to that species (dark gray). Dashed vertical lines demarcate orders. (*D*) The inferred numbers of functional recruitments (+) and losses (−) of toxin families from venom proteomes mapped onto the phylogeny of these species are indicated along the lineages, under both ACCTRAN and DELTRAN (numbers in parentheses) optimization. The number of toxin families identified in the venom of each species and those reconstructed to be present in the hypothetical ancestral venoms are indicated in circles. Abbreviations of species names are as follows: Sc*, Scutigera coleoptrata*; Tl*: Thereuopoda longicornis*; Lf*: Lithobius forficatus*; Ct*: Craterostigmus tasmanianus*; Stm*: Strigamia maritima*; Er*: Ethmostigmus rubripes*; Cw*: Cormocephalus westwoodi*; Ss*: Scolopendra subspinipes*; Sm*: Scolopendra morsitans*.

Mapping the sequences identified in the venom gland transcriptomes (fig. 2*A*) and venom proteomes (figs. 2*B* and 3) of all species revealed dramatic differences in the overall number and diversity of unique toxin contigs, as well as venom complexity (the number of toxin families). There were considerable differences on the level of toxin families, with no family showing consistently high diversity of contigs across all taxa, and no toxin family was detected in the venom proteomes of all species. Moreover, each species has its own unique toxin profile, with profiles differing greatly between species. The estimates of venom composition were markedly different depending on whether they were based on transcriptome or proteome data. Such a discrepancy of transcriptomic versus proteomic venom profiles has been noted before for centipedes, as well as other taxa, including snakes, cone snails, cnidarians, insects, scorpions, and crustaceans (Werren et al. 2010; Casewell et al. 2014; Gacesa et al. 2015; Madio et al. 2017; Von Reumont et al. 2017; Drukewitz et al. 2018; Smith and Undheim 2018; Ward and Rokyta 2018). Although methodological or expression-level related factors may contribute to these discrepancies, an important reason why transcriptomic venom profiles on their own can be misleading is that venom toxins evolve from nontoxin ancestral proteins and peptides. Homology searches, such as BLAST, can seriously overestimate venom complexity by not distinguishing toxin and nontoxin homologs, and even erroneously annotate nontoxin gene families as toxins (Madio et al. 2017; Smith and Undheim 2018).

Variation in sequencing depth can also complicate the comparison of transcriptomic venom profiles. The six transcriptomes generated on the Illumina NextSeq platform contain more contigs than those generated with Roche 454 technology. This impact of sequencing method is revealed when we perform a phylogenetic analysis of the distribution of toxin families, using a data set that scored the absence/presence of each family in the transcriptomes (supplementary fig. S1, Supplementary Material online). The strict consensus of the two most parsimonious trees groups the species based on sequencing method rather than phylogenetic relationship. The six species sequenced with Illumina NextSeq technology form a paraphyletic grouping within which is nested a clade formed of the four species sequenced with Roche 454 technology, which is exclusively supported by the losses of transcripts. Our interpretations of the composition and evolution of centipede venoms are therefore guided by our proteomic data.

Our proteotranscriptomic data show that there is no such thing as a typical or representative centipede venom (figs. 2*B* and 3, supplementary material S3, Supplementary Material online). We found large differences in the number and identity of toxin families contained in each venom, with the most complex venom containing six times as many families as the simplest one. The two *Scolopendra* species have the most complex venoms, with 47 (*S. morsitans*) and 48 (*S. subspinipes*) toxin families. The scutigeromorphs and the geophilomorph have the simplest venoms, with 8 and 12 toxin families in the venoms of the scutigeromorphs *Scutigera coleoptrata* and *T. longicornis*, respectively, and 12 toxin families in the venom of the geophilomorph *Stm. maritima*. The other species have venoms of intermediate complexity, with the number of toxin families ranging from 17 in the craterostigmomorph *C. tasmanianus* to 28 in the scolopendromorph *Ethmostigmus rubripes*. Furthermore, the venoms of most species contain between three and eight unique toxin families, except the scutigeromorph *Sc. coleoptrata* and the scolopendromorph *Cormocephalus westwoodi*, with there being no toxin families unique for these species. When judged by the number of unique relative to the total number of toxin families found in their venoms, the craterostigmomorph and the lithobiomorph venoms emerge as the most distinctive, with respectively 47% and 35% of their putative toxin families not being found in any other species (fig. 2*B*, supplementary material S3, Supplementary Material online). We note that because the transcriptomic and proteomic venom profiles for *L. forficatus* were sampled from different populations our data provide a minimum estimate of venom complexity in this species.

We also found that venom composition was remarkably poorly conserved between taxa (fig. 3*C*), with none of the 93 identified toxin families being present in the venoms of all five orders. Only 3 toxin families are found in four of the orders, another 3 are found in three orders, 20 families are present in two orders, and 67 families are only found in a single order. Of these single-order families, 35 were found in orders with more than one sampled species (Scutigeromorpha and Scolopendromorpha). Eighteen of these were found only in the venom of a single species, suggesting that our estimates of venom composition conservation are not an artifact due to our limited species sampling within each order, and suggesting that most of the compositional evolution of centipede venoms occurred within the extant orders.

Although there is no toxin family uniquely diagnostic for centipede venoms, all clades in our species tree do have synapomorphic toxin families (supplementary material S4, Supplementary Material online). Moreover, the clades Scutigeromorpha, Epimorpha, Scolopendromorpha, and the genus *Scolopendra*, all have unique synapomorphic toxin families that are present in all species in the clade. We therefore tested whether the protein composition of centipede venoms preserves the phylogenetic signal of species relationships, and performed a phylogenetic analysis on a data set (supplementary material S5, Supplementary Material online) that scores the absence/presence of toxin families in the venoms of the species based on our proteomic data. The analysis resulted in nine equally parsimonious trees, the strict consensus of which reflects expected species relationships in the presence of three clades: a clade that groups the two scutigeromorph species, a clade that groups the scolopendromorph species, and a clade uniting the *Scolopendra* species (supplementary fig. S2, Supplementary Material online). However, the relationships within the scolopendromorph clade do not follow the expected pattern, and the phylogenetic positions of the geophilomorph, craterostigmomorph, and lithobiomorph species remain unresolved. These results show that venom composition retains some phylogenetic signal of the accepted species tree, but that relatively little compositional evolution happened during early centipede evolution.

### Complex Evolutionary Dynamics Govern the Evolution of Centipede Venom Protein Families

To shed light on the evolutionary processes that produced the genetic diversity that forms the basis of these striking differences in venom composition, we reconstructed the phylogenetic histories of individual toxin families. In short, outgroup sequences were identified by performing a BLAST search of all nonredundant contigs from our venom gland transcriptomes against the UniProtKB protein database as well as a custom database of myriapod transcriptomes. Contigs that were identified in the venom proteomes of our species were annotated on all gene trees (for more details see Materials and Methods). All gene alignments can be found in supplementary material S6 (Supplementary Material online) and all gene trees in supplementary figures S3–S72 (Supplementary Material online). The resulting proteomically annotated phylogenies of putative toxins and their nontoxin homologs revealed a highly complex picture of numerous gene duplication events, functional recruitments, as well as losses of toxin families from both proteomes and transcriptomes (see supplementary material S7, Supplementary Material online, for all toxin recruitment trees).

**Fig. 4.**
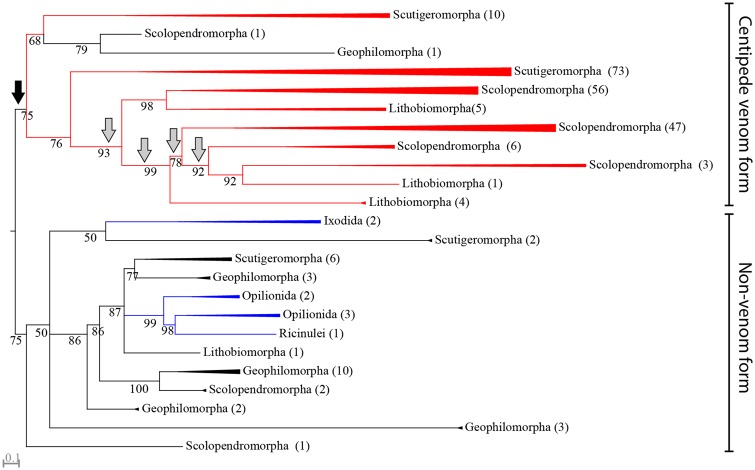
Gene duplication and loss drives the evolution of β-PFTx venom proteins. Maximum likelihood (ML) phylogenetic reconstruction of the β-PFTx family (under WAG+F+R5, chosen according to Bayesian Information Criterion) displayed as rooted with a clade containing taxonomic outgroup sequences. Gene tree/species tree reconciliation suggests a single gene duplication occurred in the ancestral centipede, indicated by a black arrow, whereas four subsequent duplications occurred along the stem lineage of Pleurostigmophora, indicated by gray arrows. Functional losses were associated with all these suggested duplication events. Clades containing only sequences from a single order are collapsed, with the number of sequences in each collapsed clade shown in parentheses. Clades containing sequences identified in venom proteomes are colored red, whereas noncentipede sequences are colored blue. Bootstrap support values are shown at each node, and nodes with support <50 are collapsed into polytomies. For the phylogenetic tree without collapsed clades see supplementary materialfigure S3 (Supplementary Material online).

An example of a family with a complex evolutionary history is the β-PFTx family, which was recruited as one of the first venom components in the common centipede ancestor. Gene tree/species tree reconciliation suggests that the evolution of this family is governed by gene family expansions as well as functional losses at different times during the evolutionary history of centipedes (fig. 4). Our data suggest that on the order level, there occurred a minimum of five duplication events within this gene family, one along the centipede stem lineage, and four along the stem lineage of pleurostigmophorans. Each of these duplications was associated with subsequent functional losses, especially in Geophilomorpha and Craterostigmomorpha, which have just a single or no venom β-PFTx sequences, respectively. This complex scenario of duplications and losses is also evident within centipede orders, particularly in the diversification of the β-PFTx family within Scutigeromorpha and Scolopendromorpha (fig. 4, supplementary fig. S3, Supplementary Material online). In both of these orders, two separate clades of β-PFTx have undergone extensive convergent gene family expansions at the base of the families examined (Scutigeridae in Scutigeromorpha and Scolopendridae in Scolopendromorpha). However, of these β-PFTx clades, only the two that diversified in the scolopendromorphs show convincing evidence of numerous losses of β-PFTx forms from both the venom proteomes and transcriptomes of species.

**Fig. 5.**
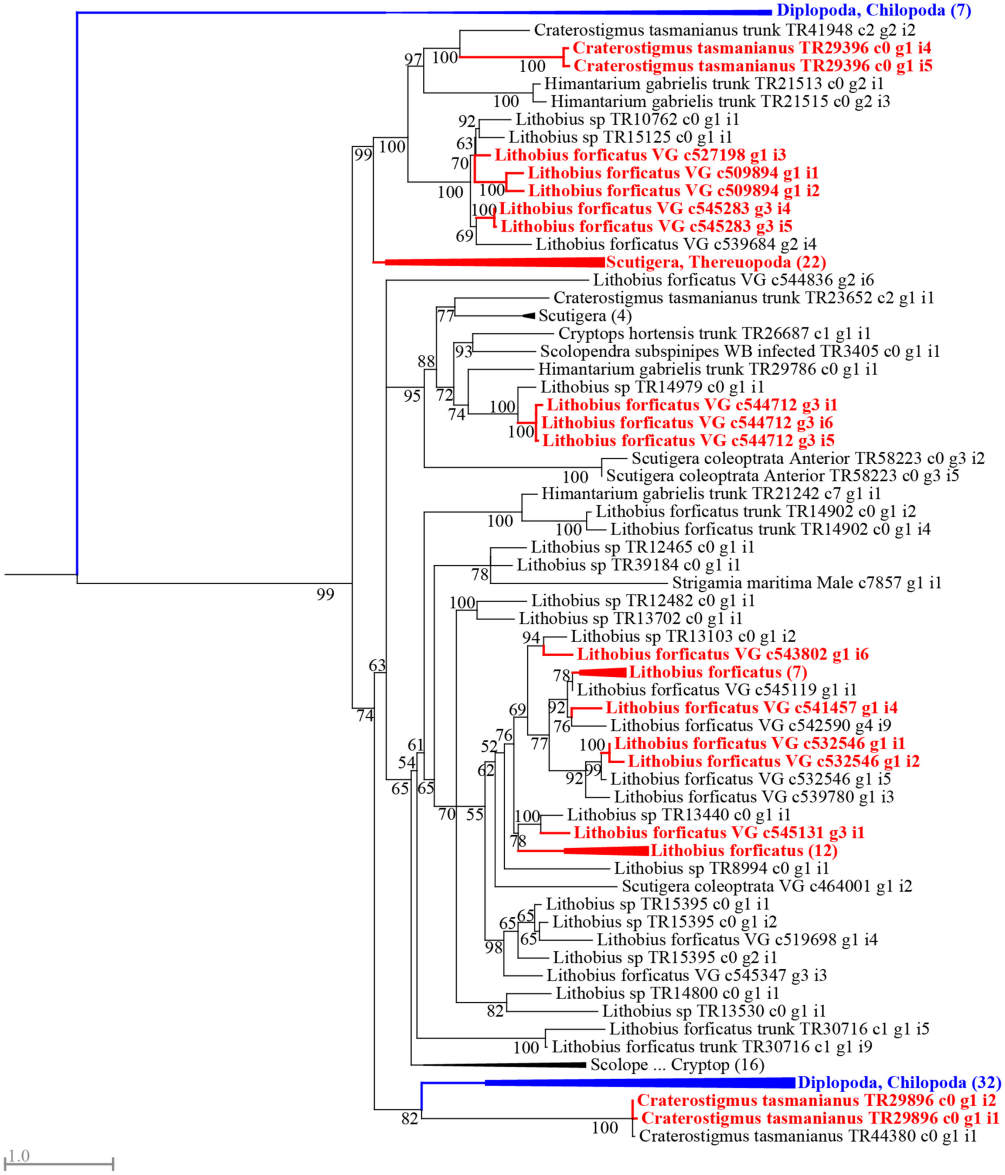
The complex evolutionary history of the centipede M12A toxin family. Phylogenetic reconstruction of the myriapod M12A protein family by maximum likelihood (ML) under WAG+F+R7 (chosen according to Bayesian Information Criterion) displayed as a midpoint rooted tree. Sequences identified in venom proteomes are colored red, whereas collapsed clades also containing nonchilopod sequences are colored blue. Clades containing only sequences identified in venom proteomes are collapsed, with the number of sequences indicated in parentheses, except the clade “Scutigera, Thereuopoda (22),” which contains 19 proteome-identified sequences and 3 nonproteome sequences. Bootstrap support values are shown at each node, and nodes with support <50 are collapsed into polytomies. For the phylogenetic tree without collapsed clades see supplementary figure S4 (Supplementary Material online).

Such taxonomically restricted evolution is common for centipede venom toxin families. This pattern is evidenced by the number of order-specific toxin families, but it is also evident among toxin families that are shared between orders. In these taxonomically more widespread toxin families, the main radiations appear to have occurred not within orthologous toxin clades, but ancient paralogous toxin clades. Examples of this include β-PFTx, where about half of the main toxin clades show evidence of centipede lineage-specific diversification (fig. 4), and M12A (astacin-like Zn-metalloprotease, MEROPS family M12A). The M12A tree (fig. 5) suggests that this family has diversified substantially in both Scutigeromorpha and Lithobiomorpha, but not in orthologous clades. Instead, the main diversification in Scutigeromorpha is located in the upper-paralogous clade (99% bootstrap support), whereas the lithobiomorph diversification happened in the lower-paralogous clade (74% bootstrap support). These lineage-specific evolutionary trends paint a picture of a highly plastic venom system that has evolved without strong constraints from an existing “core” set of pharmacologically crucial components.

In addition to lineage-specific toxin diversification and multiple independent recruitments of single protein and peptide families, phylogenetic analyses revealed several cases of paralogous toxin clades densely interspersed by closely related but likely nontoxin centipede homologs, including nonvenom gland (trunk) sequences. This pattern was found in eight toxin families, namely the M12A proteases (fig. 5), GH18 hydrolases (chitinase-like glycoside hydrolase, CAZY family GH18), GGT (γ-Glutamyltransferase), PCPDP-like proteins (Pesticidal Crystal Protein Domain-containing Protein), transferrin, CAP proteins, SLPTX04, and SLPTX16. Although we cannot discard the possibility of numerous independent recruitments, this pattern is suggestive of reciprocal functional recruitments, both from physiological to venom functions and from venom to physiological functions inside and/or outside the venom gland. One example of a possible reverse recruitment of a toxin back to a physiological role can be found in the collapsed clade of scutigeromorph sequences labeled “Scutigera, Thereuopoda (22)” in the M12A tree (fig. 5). This clade consists of a paraphyletic group of sequences found in the venom proteome within which are nested three sequences that are not found in the proteome, and that come from a transcriptome that is not venom gland-specific (supplementary fig. S4, Supplementary Material online). Such a scenario of dynamic toxin–nontoxin evolution has to our knowledge only been described from snake venoms (Casewell et al. 2012). Our results suggest this might be a more widespread phenomenon, particularly for toxin types with activities closely related to that of their physiological ancestors. However, comprehensive sampling of nonvenom gland tissues is needed to properly test this idea. Alternatively, the differential presence of paralogs from the same toxin family in venom may signify the adaptive fine-tuning of venom composition to local circumstances. This interpretation is suggested by research that demonstrated that several snake species can differentially regulate transcription/translation of individual toxin paralogs, causing a lack of correlation between the transcriptomic and proteomic abundances of paralogs (Casewell et al. 2014; Amazonas et al. 2018). The differential expression of such paralogs between different individuals, especially those with lower-expression levels, may record adaptive variation in venom composition in response to environmental changes.

### The Compositional Evolution of Centipede Venom Is Highly Dynamic

**Fig. 6.**
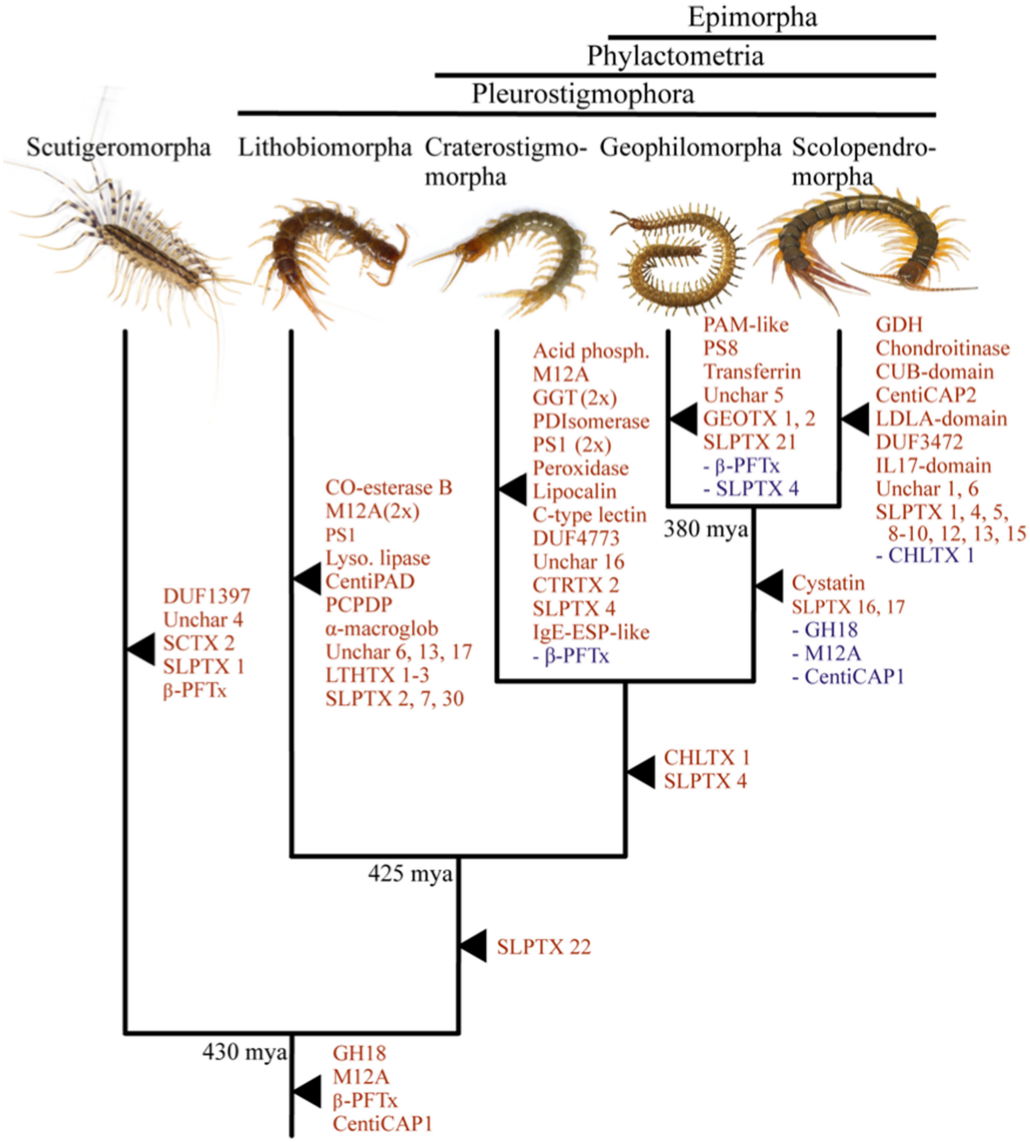
The evolutionary history of centipede venom composition. Order-level phylogeny of centipedes, with ACCTRAN optimized functional recruitments (red) and losses (blue) of toxin families identified in the venom proteomes of the species analyzed in this study. Changes that are synapomorphic for the orders are shown; for changes within the orders see supplementary material S4. The estimated clade ages, in millions of years ago (Ma), are from Fernández et al. (2016).

The processes that shape the genetic diversity of toxin families set the parameters for what is possible during venom evolution, but our data show that the evolution of venom composition has its own distinctive dynamics. We therefore reconstructed the macroevolutionary history of functional recruitments and losses of toxin families from centipede venom proteomes, using parsimony-based character state optimizations informed by our phylogenetic analyses of toxin gene families (figs. 3 and 6) (see supplementary materials S4 and S7, Supplementary Material online, for all character optimization results). We used both DELTRAN (Delayed Transformation) and ACCTRAN (Accelerated Transformation) optimization to reconstruct the evolution of venom composition, but we focused our main discussions around the latter because it presents a conservative estimate of the extent to which centipede venom complexity evolved in parallel within the five orders, while maximizing the amount of early compositional evolution that is inferred along shared ancestral lineages. Our analyses reveal a highly dynamic picture of venom evolution, with numerous recruitments of toxin families increasing venom complexity in parallel in all lineages, and functional loss of toxin families streamlining venoms. Seventy-one toxin families were recruited into venoms once, whereas 22 families were recruited convergently in different parts of the centipede tree. Toxin families were lost from centipede venoms between 12 (DELTRAN) and 24 (ACCTRAN) times across the species tree. In the latter optimization, this involved 21 toxin families, but only GH18, β-PFTx, and SLPTX04 were lost convergently.

The functional loss of toxin families has streamlined venoms in all lineages except those of the scutigeromorph *Sc. coleoptrata* and the lithobiomorph *L. forficatus*. In contrast, the venoms of the scolopendromorph *Co. westwoodi* and the geophilomorph *Stm. maritima* show that protein family loss can have major impacts on venom composition. Along the lineage of *Stm. maritima* 17 protein families were recruited into the venom, whereas five families were lost from the venom, including all four ancestral centipede venom protein families. Losses have streamlined the venom of *Co. westwoodi* even more, with 14 families lost and 32 families recruited since it diverged from the last common centipede ancestor. *Cormocephalus westwoodi* is the only species in our study for which we were able to infer an actual decrease in venom complexity after it diverged from its last common ancestor with the genus *Scolopendra*. The reasons for such venom streamlining remain unclear, but the loss of venom complexity has been linked to dietary shifts in snakes and heteropteran insects (Li et al. 2005; Walker et al. 2018).

We confirmed that *Co. westwoodi’*s venom does not lack complexity due to the “shallow” Roche 454 sequencing of its venom gland transcriptome by incorporating the recently published venom of *S. viridis* into our recruitment analyses. When placed in our species tree, *S. viridis* is the closest relative of *Co. westwoodi* according to current molecular phylogenetic insights (Vahtera et al. 2013). Ward and Rokyta (2018) sequenced the venom gland transcriptome of an *S. viridis* individual with Illumina HiSeq technology, which they used to identify 39 protein families in its venom proteome. These represent 16 toxin families when reclassified according to the phylogeny-based toxin classification adopted in our paper. This makes the venom of *S. viridis* the simplest scolopendromorph venom known. Interestingly, character optimization (see supplementary materials S4 and S8, Supplementary Material online) suggests that the main cause of the simplicity of the venoms of *Co. westwoodi* and *S. viridis* is the evolutionary streamlining of venom along their shared ancestral lineage, with the loss of seven protein families. One and three more protein families were subsequently lost along the branches leading to *Co. westwoodi* and *S. viridis*, respectively. The evolutionary streamlining of the venoms of these species is less prominent under DELTRAN optimization, with the loss of two families along their shared lineage, as well as one and two additional losses along the lineages of *Co. westwoodi* and *S. viridis*, respectively.

### The Evolutionary History of Centipede Venom

Our analyses of the compositional evolution of centipede venoms allow us to generate a first reconstruction of the evolutionary history of centipede venoms. Our ancestral state reconstructions revealed that the ancestral centipede venom was probably a simple cocktail containing just two enzymes (GH18 and M12A), a putative pore-forming toxin (β-PFTx), and a cysteine-rich protein (CAP1) (fig. 6). This finding contrasts with the previously reconstructed ancestral centipede venom, which included another six protein and three peptide families, while lacking GH18 (Undheim, Jones, et al. 2014; Undheim, Fry, et al. 2015). Although direct experimental evidence for the bioactivities and roles of these centipede venom components is currently lacking, it is reasonable to suggest that if our ancestral reconstruction is accurate, the toxins together must constitute a functional arsenal, and in this combination, we propose that this is indeed the case. We speculate that CAP1 and β-PFTx would have been primarily responsible for prey immobilization. CAP proteins function as neurotoxins in many venoms (Fry et al. 2009), and centipede CAP2 has been shown to inhibit voltage-gated calcium channels (Undheim, Fry, et al. 2015). β-PFTx may achieve neurotoxicity through pore formation in cell membranes, in a manner similar to the strongly neurotoxic pore-forming latrotoxin from black-widow spider venom (Garb and Hayashi 2013), and it has been hypothesized that β-PFTx may be responsible for neurotoxic effects on cockroach nerves observed in earlier studies of centipede venom (Undheim, Fry, et al. 2015). M12A is an endopeptidase, and we speculate that it acts as a spreading factor that degrades matrix proteins, as has been suggested for spider venom M12A (Undheim, Fry, et al. 2015). Moreover, since M12A is the only protease present in the ancestral venom, it probably also acted as an activator of β-PFTx, which requires activation by proteolysis (Undheim, Fry, et al. 2015; Ward and Rokyta 2018). Finally, GH18, which comprises chitinases and chitotriosidase in the centipede venoms, may act to dislodge soft tissue from its anchor points on the chitinous exoskeleton of the prey, in a manner similar to that proposed for the venoms of octopuses and remipede crustaceans (Von Reumont et al. 2017). Like these taxa centipedes prefer to hollow out rather than ingest the more chitinous parts of their arthropod prey (Lewis 1981). These components together may thus act as an effective venom arsenal for the immobilization and initial processing of prey.

Our ancestral reconstructions suggest that centipede venoms remained simple cocktails for much of the early evolutionary history of the group, with relatively little compositional evolution occurring along the lineages leading to Pleurostigmophora, Phylactometria, and Epimorpha. It is possible that more compositional evolution occurred in these basal parts of the centipede tree, but that this has become obscured by subsequent evolution. This is unlikely, however, because these internodes also have short branch lengths in phylogenomic trees and chronograms of centipede relationships. That indicates that these internodes bracket relatively small amounts of molecular evolution and evolutionary time compared with the order-level lineages diverging from these internodes (Fernández et al. 2014, 2016). Moreover, the lack of compositional venom evolution in this basal part of the tree is matched by a correspondingly small number of family recruitments and losses occurring on the transcriptomic level (supplementary material S4, Supplementary Material online). Most compositional evolution has clearly happened after the orders split from each other. Seventy-eight percent of the total amount of compositional evolution (115 of 147 recruitments/losses) happened within the five orders, with only 22% occurring along their shared stem lineages (fig. 3).

Venom complexity evolved in parallel along all species lineages, and the scutigeromorph, phylactometrian, and epimorphan ancestors evolved venoms with roughly double the gene family diversity of the ancestral centipede venom (figs. 3 and 6). Along the lineage leading to Scutigeromorpha, four protein families were recruited into the venom, DUF1397 (a protein with a *d*omain of *u*nknown *f*unction), uncharacterized protein family 4 (a protein family with no similarity to characterized proteins or domains), Scutigerotoxin 2 (SCTX02), and SLPTX01. The first three of these are unique for scutigeromorphs, whereas SLPTX01 has been convergently recruited into scolopendromorph venoms. Interestingly, the venom of *Sc. coleoptrata* is identical to that reconstructed for the scutigeromorph ancestor, whereas *T. longicornis* has recruited an additional five protein families and lost GH18 from both its venom and venom gland transcriptome. The pleurostigmophoran ancestor recruited just one additional protein family to the ancestral centipede venom, the peptide family SLPTX22. A modest increase in venom complexity subsequently evolved along the stem lineage leading to Phylactometria, with the recruitment of two protein families (CHLTX01 and SLPTX04). Three more protein families (cystatin, SLPTX16, SLPTX17) were recruited into the ancestral epimorphan venom, but its complexity remained the same as that of the phylactometrian ancestor (seven protein families) because of the loss of three protein families along the epimorphan stem lineage (GH18, M12A, CAP1). These increases in venom complexity along the stem lineages of Pleurostigmophora, Phylactometria, and Epimorpha are with the exception of a single protein family (cystatin) entirely due to the recruitment of peptides, resulting in an increase in the proportion of venom peptide families from none in the ancestral centipede venom, to 20% in the ancestral pleurostigmophoran venom, 43% in the ancestral phylactometrian venom, and 71% in the ancestral epimorphan venom (fig. 3*B*).

A much more substantial increase in venom complexity evolved at the base of Scolopendromorpha, with the recruitment of 16 new protein families, and the loss of only 1. With an estimated 23 venom protein families, the ancestral scolopendromorph venom was almost 6 times more complex than that of the last common centipede ancestor. A further modest increase in venom complexity evolved along the stem lineage of Scolopendrinae (the clade containing *Co. westwoodi* and the two *Scolopendra* species) with the recruitment of 4 protein families and the loss of 1, before another massive increase in venom complexity evolved along the base of the genus *Scolopendra*, with the recruitment of 17 and the loss of 2 protein families. Although the reason for this sudden increase in toxin diversity is unclear, it is tempting to speculate that it may be related to a combination of increases in venom gland complexity and body size, which enabled an ecological shift from largely arthropod-restricted prey to an opportunistic and more diverse diet that included larger vertebrate prey. An increase in the diversity of venom toxins has been correlated with a change to a more diverse diet in cone snails and spiders (Phuong et al. 2016; Pekár et al. 2018; Phuong and Mahardika 2018), and in centipedes could have driven an increase in complexity of both the venom and the associated venom gland (Undheim, Hamilton, et al. 2015).

Although similarly systematic and comprehensive studies of the compositional evolution of venoms are currently lacking for other taxa, relevant insights have been generated for several venomous groups, notably reptiles, cnidarians, and heteropteran insects (Fry et al. 2012; Jaimes-Becerra et al. 2017; Walker et al. 2018). Much of the compositional evolution of venoms in these groups was inferred to have happened early in their evolutionary histories, with venoms first emerging as complex venom cocktails. Sunagar and Moran (2015) formalized this insight in what they called the “two-speed model” of venom evolution, according to which a rapid diversification of the venom arsenal early in the evolutionary history of a venomous lineage would be followed by a longer period of purification and fixation to preserve the toxic potency of the venom. Although this model was formulated on the basis of comparative sequence analyses, neither it nor the early evolution of complex venoms in reptiles or advanced snakes, cnidarians, and heteropterans correspond well with our findings about centipede venom composition. Our data do not suggest a rapid early expansion of the centipede venom arsenal, but instead a dynamic picture of compositional evolution throughout the phylogenetic history of the group, with parallel increases in venom complexity within all five orders. Extensive compositional evolution also happened between taxa of much more recent origins, including congeneric species, such as *S. subspinipes* and *S. morsitans*, and even within species, as has been shown for *S. subspinipes* and *S. viridis* (Smith and Undheim 2018; Ward and Rokyta 2018).

The new insights generated by our study are of course conditional upon our broad, but nevertheless limited, taxon sampling. Future studies aiming to produce a more detailed understanding of the patterns and processes of centipede venom evolution will benefit from generating genomic resources for the major lineages, sampling a larger number of species within them, as well as estimating the divergence times of all relevant clades. However, the results we present here strikingly illustrate the contrast between early and late compositional evolution of centipede venoms. More compositional evolution happened along the lineage of *S. subspinipes* since it diverged from its congener *S. morsitans* than happened along the 50 My or so that span the combined stem lineages of Chilopoda, Pleurostigmophora, Phylactometria, and Epimorpha.

## Conclusions

Centipedes have recently been receiving increasing attention as sources of novel bioactive peptides with potential for development into molecular tools and human therapeutics (Undheim et al. 2016). However, biodiscovery-driven research tends to focus on species that are easily obtainable and yield sufficient quantities of venom (Herzig et al. 2019). Consequently, our understanding of the composition and evolution of centipede venoms has been heavily biased toward large-bodied species in the scolopendromorph family Scolopendridae. A danger of this bias is that some venom researchers have come to regard these scolopendromorphs as representative centipedes, incorrectly attributing their traits to centipedes in general, including incorrect generalizations regarding predation on vertebrate prey (e.g., Chen et al. 2014, p. 159; Hakim et al. 2015, p. 4833), and the clinical relevance of centipede bites (e.g., Yang et al. 2012, p. 640; Yang et al. 2015, p. 2). Our results clearly show that scolopendromorph venoms are not representative for centipedes as a whole, but neither are the venoms of the other orders. All have diverged substantially from a simple ancestral venom, with complex venoms evolving in parallel within the orders, with strikingly unique toxin cocktails resulting from the exclusive recruitment of most toxin families into the venoms of single orders.

To our knowledge, this is the first study that uses a combination of proteome-annotated gene tree analyses and explicit character optimization algorithms to reconstruct the origin and compositional evolution of venom across an entire group. It thus substantially broadens the empirical, taxonomic, and conceptual foundation of centipede venomics, as well as our understanding of venom evolution more generally. Our results show that even venoms composed of toxins that are under pervasive strong negative selection (Undheim, Sunagar, et al. 2014; Sunagar and Moran 2015) can have a striking level of evolutionary plasticity on the level of toxin composition. Functional recruitment and loss of venom components have generally been considered relatively rare events compared with functional radiation of existing venom components by structural diversification. However, our findings highlight the important roles that functional recruitment and loss of toxin families can play throughout the evolutionary history of venomous groups.

## Materials and Methods

### Species Selection and Specimen Collection

To gain an overview of the evolution of centipede venom across all five extant orders, we analyzed the previously unstudied venoms of four species using a combined transcriptomic and proteomic (proteotranscriptomic) approach: *Sc. coleoptrata* (Scutigeromorpha), *L. forficatus* (Lithobiomorpha), *C. tasmanianus* (Craterostigmomorpha), and *Stm. maritima* (Geophilomorpha). We also resequenced *S. morsitans* (Scolopendromorpha), previously analyzed by Undheim, Jones, et al. (2014), in order to assess potential experimental artifacts stemming from sequencing depth and technology. We compared these newly generated transcriptome and proteome data to previously published venom and venom gland proteotranscriptomic data sets for the scutigeromorph *T. longicornis* (NCBI transcriptome shotgun assembly [TSA] accession GASR) and the scolopendromorphs *E. rubripes* (TSA accession GASI), *Co. westwoodi* (TSA accession GASL), and *S. morsitans* (TSA accession GASH). The data for these species were analyzed using the same proteotranscriptomic and phylogenetic approach as used here, but their transcriptomes were sequenced with Roche 454 technology and assembled using MIRA (Undheim, Jones, et al. 2014). We also included *S. subspinipes* (TSA accession GGDW), which was analyzed according to the same protocols used here and sequenced on an Illumina NextSeq platform (Smith and Undheim 2018). The proteotranscriptomic study of *S. viridis* venom from Florida by Ward and Rokyta (2018) was published too late for us to reanalyze their data with our analytic pipeline. However, we did incorporate their results into our analyses of venom protein family recruitment as described below.

Eight *L. forficatus* specimens were collected at Tooting Bec Common (seven specimens) and near Hither Green (one specimen) in London, United Kingdom, and forcipules from these specimens were dissected and pooled for transcriptomic profiling. Specimens for proteomic analyses were collected in Greifswald, Germany. Specimens of *Stm. maritima* for transcriptomic and proteomic analyses were collected near Brora, Scotland. The forcipules of 80 males and 57 females were dissected and pooled for each sex to create 2 transcriptomic profiles. These profiles were combined for all downstream analyses. Specimens of *Sc. coleoptrata* for transcriptomic and proteomic analyses were collected in Ibiza, Spain. The forcipules of 20 specimens were dissected and pooled for transcriptomic profiling. Specimens of *C. tasmanianus* for transcriptomic and proteomic analyses were collected near Hellyer Gorge in Tasmania, Australia, and forcipules from five adults dissected and pooled for transcriptomic profiling. The regenerating venom glands from a single female *S. morsitans* collected in Glenmorgan, Queensland, Australia were resequenced as well.

### Transcriptomics

For *L. forficatus, C. tasmanianus*, *Stm. maritima*, and *S. morsitans*, we sequenced the transcriptomes of forcipules or dissected venom glands (*S. morsitans*) 2 days after depleting venom by electrostimulation. The forcipules of *Sc. coleoptrata* were dissected without depleting the venom glands beforehand because the animals recovered very poorly from electrostimulation. Total RNA was extracted from all samples using standard TRIzol protocol (ThermoFisher). For *C. tasmanianus* and *S. morsitans*, samples were submitted to the University of Queensland Institute for Molecular Bioscience Sequencing Facility for library preparation and sequencing. Paired-end libraries with 180 bp insert size were constructed using the Illumina TruSeq-3 Stranded mRNA kit and sequenced on an Illumina NextSeq using a 300 cycle (2 × 150bp) Mid Output Run. The samples for *Stm. maritima*, *L. forficatus*, and *Sc. coleoptrata* were submitted to the Natural Environment Research Council Center for Genomic Research at the University of Liverpool for library preparation and sequencing. The libraries were paired-end sequenced on an Illumina HiSeq2000 platform, with 100 bp insert size. The resulting reads were trimmed with Trimmomatic 0.35 (Bolger et al. 2014) with a phred score of 20 and minimum length of 50 bp for *L. forficatus*, *Sc. coleoptrata*, *C. tasmanianus*, and *S. morsitans*. For *Stm. maritima* the resulting reads were examined for sequence quality with FastQC (Andrews 2010), then processed with NGSQC Toolkit v2.3.3 (Patel and Jain 2012) to remove low quality reads (IlluQC_PRLL.pl) with PHRED score of <20, then trimmed by 10 bp on the 5′ end to remove low quality/biased base calls (TrimmingReads.pl) using a sequence length cutoff of 75 bp. The processed FastQ reads were subsequently assembled for all samples with Trinity v.2014-07-17 (Grabherr et al. 2011) applying the standard settings except a minimum length of 101 bp. Seqclean was used with the flags –N –A –M to find and exclude remaining contaminant sequences from assembled transcripts (https://sourceforge.net/projects/seqclean/files/; last accessed August 14, 2019).

### Proteomics

To identify proteins and peptides present in the venom, venom obtained by electrostimulation was analyzed by a bottom-up approach. For each sample, 5 µg crude venom was dried by vacuum-centrifugation and redissolved in 4 M urea 10% ACN 100 mM ammonium bicarbonate, pH 8. Cystines were then reduced by incubating with 5 mM dithiothreitol at 70 °C for 5 min and alkylated with 10 mM iodoacetamide at 37 °C for 90 min. The reduced and alkylated samples were then digested by incubating with 30 µg/µl trypsin overnight at 37 °C in 2 M urea 10% ACN 100 mM ammonium bicarbonate, pH 8, at a final substrate to enzyme ratio of approximately 100:1. The resulting tryptic peptides were desalted using a C18 ZipTip (ThermoFisher), dried using vacuum centrifugation, dissolved in 0.5% formic acid (FA), and 2 µg analyzed by liquid chromatography tandem mass spectrometry (LC-MS/MS) on an AB Sciex 5600 TripleTOF equipped with a Turbo-V source heated to 550 °C. The dissolved samples were fractionated on a Shimadzu (Kyoto, Japan) Nexera UHPLC with an Agilent Zorbax stable-bond C18 column (Agilent) (2.1 × 100 mm, 1.8 µm particle size, 300 Å pore size), using a flow rate of 180 µl/min and a gradient of 1–40% solvent B (90% acetonitrile [ACN], 0.1% FA) in 0.1% FA over 60 min. MS1 spectra were acquired at 300–1800 m/z with an accumulation time of 250 ms, and selecting the 20 most intense ions for MS2. Precursor ions with a charge of +2 to +5 and an intensity of at least 120 counts/s were selected, with a unit mass precursor ion inclusion window of ±0.7 Da, and isotopes within ±2 Da were excluded. MS2 scans were acquired at 80–1400 m/z, with an accumulation time of 100 ms, and optimized for high resolution.

The resulting MS/MS spectra were then searched against the respective species’ assembled transcriptomes translated to all 6 possible open reading frames longer than 40 amino acids using the Galaxy tool “Get open reading frames (ORFs) or coding sequences (CDSs)” (Cock et al. 2013) using Protein Pilot v4.5 (AB SCIEX). Although biological modifications were allowed, we did not allow for amino acid substitutions in an attempt to reduce the number of false positive identifications of any similar nontoxin homologs. False positives were identified using decoy-based false discovery rates (FDR) as estimated by Protein Pilot, and only protein identifications with a corresponding local FDR of <0.5% were considered significant. Identified contigs were filtered using CD-HIT v4.6 (Li and Godzik 2006) to remove identical sequences.

### Data Availability

All raw sequence data were submitted to the Sequence Read Archive (SRA) of the National Center for Biotechnology Information (NCBI) under BioProject ID PRJNA540703, with BioSample accessions SAMN11553657, SAMN11553658, SAMN11553659, SAMN11553660, SAMN11553661, and SAMN11553662. Transcriptome assemblies are available via the Natural History Museum’s Data Portal (https://data.nhm.ac.uk/dataset/evolution-of-centipede-venoms; last accessed July 23, 2019). All mass spectrometry and ProteinPilot data were submitted to the ProteomeXchange Consortium via the PRIDE (Vizcaíno et al. 2016) partner repository with the data set identifier PXD006253.

### Phylogenetic Analyses and Ancestral State Reconstruction of Venom Composition

To assess the retention of phylogenetic signal in the composition of the venoms two data sets were prepared that scored the absence/presence of all 93 protein families in the venom gland transcriptomes and venom proteomes of the species, respectively. We also added the Florida *S. viridis* to the proteome data set, based on the results described in Ward and Rokyta (2018). We reclassified the toxins they found in *S. viridis* venom according to the gene tree-based classification of toxin families used in the current paper, combining toxin families if they were part of monophyletic clades separate from other families. For example, we grouped the six β-PFTx families reported by Ward and Rokyta to be present in *S. viridis* into a single family because all centipede β-PFTxs are monophyletic and distinct from other centipede toxin families. This reduced the number of *S. viridis* toxin families from 39 to 16. PAUP* v4.0a, builds 164 and 165 for Macintosh (Swofford 2002), was used to perform phylogenetic analyses of these data sets, using parsimony as optimality criterion, and Branch and Bound as the search algorithm. A strict consensus tree was constructed to summarize the agreement between the equally parsimonious trees (supplementary figs. S1 and S2, Supplementary Material online). These data sets were also used in PAUP* to reconstruct protein family recruitments and losses along the accepted species tree and to infer ancestral venom cocktails using the parsimony character optimization algorithms ACCTRAN and DELTRAN. Because we lack a species tree with meaningful branch lengths, we did not use model-based ancestral state reconstructions. All recruitment trees can be found in supplementary material S7 (Supplementary Material online). ACCTRAN infers character transformations as close as possible to the root of the tree, whereas DELTRAN optimizes character transformation closer to the tips of the tree. For our absence/presence-coded data this means that ACCTRAN favors early recruitment of protein families followed by loss over convergent recruitments, and vice versa for DELTRAN. We adjusted the protein family recruitments and losses reconstructed using PAUP* with the information provided by the gene trees we constructed for the venom protein families, which include information about probable gene duplications and the distribution of transcripts found in the venom proteomes. Consequently, we adjusted the optimization of recruitment events for these families: GGT, M12A, S1, β-PFTx, SLPTX03, and SLPTX04.

### Species Tree Topology

The topology of the species tree used to interpret the evolutionary dynamics of venom composition follows our current understanding of the order-level relationships of centipedes (Fernández et al. 2014, 2016, 2018). However, the phylogenetic position of Craterostigmomorpha remains uncertain. They are either the sister group to Epimorpha in a clade called Phylactometria, or they are the sister group to the other pleurostigmophorans in a clade called Amalpighiata. Because the first hypothesis is in better agreement with available morphological evidence (Fernández et al. 2014) we have interpreted our data in the context of this hypothesis. For relationships within Scolopendromorpha we relied on the molecular phylogenetic results of Vahtera et al. (2013).

### Phylogenetic Analyses of Toxin Families

To obtain nontoxin homologs to use as outgroups, we conducted BLAST searches (BLAST+, BlastP, *E*-value cutoff 10^−6^) of all nonredundant contigs (removing identical amino acid sequences with CD-HIT) belonging to each of the 93 identified putative toxin gene families against the full UniProtKB protein database (accessed May 10, 2016) (Camacho et al. 2009; The UniProt 2017). We also searched a custom database of 16 translated de novo assembled myriapod transcriptomes obtained from the NCBI SRA that included eight millipede whole body and eight centipede whole body or trunk transcriptomes (supplementary material S9, Supplementary Material online). We examined and filtered false positives using CLC Main WorkBench v7 (Qiagen, Aarhus, Denmark) and Geneious version 11.1.5 (https://www.geneious.com; last accessed August 14, 2019), then aligned all significant hits using the local paired iterative alignment method (L-INS-i) in MAFFT v7.304b (Katoh and Standley 2013), and used a molecular phylogenetic approach to examine the evolutionary histories of all 70 of the 93 identified toxin gene families for which we found four or more representatives (all alignments are included in supplementary material S6 (Supplementary Material online) and all phylogenies in supplementary figures, Supplementary Material online). We determined the most appropriate evolutionary model using ModelFinder (Kalyaanamoorthy et al. 2017) and used IQ-Tree v1.5.5 (Nguyen et al. 2015) for reconstruction of molecular phylogenies by maximum likelihood, and estimated branch support values by ultrafast bootstrap using 10,000 replicates (Minh et al. 2013). Finally, we used Archaeopteryx v0.9921 (Han and Zmasek 2009) to map the presence of all sequences confirmed in the venom proteomes onto each gene family phylogeny in order to distinguish bona fide venom components from their nontoxin counterparts, and to distinguish single from multiple toxin recruitment events.

## Supplementary Material

Supplementary data are available at *Molecular Biology and Evolution* online.

## Supplementary Material

msz181_Supplementary_Data
