# Corrigendum to: Parallel Evolution of Complex Centipede Venoms Revealed by Comparative Proteotranscriptomic Analyses

**DOI:** 10.1093/molbev/msab180

**Published:** 2021-07-16

**Authors:** Ronald A Jenner, Bjoern M von Reumont, Lahcen I Campbell, Eivind A B Undheim

*Molecular Biology and Evolution*, Volume 36, Issue 12, December 2019, Pages 2748–2763, https://doi.org/10.1093/molbev/msz181

In the original article, the legend of Supplementary Figure S64 incorrectly refers to uncharacterised protein family 5. The legend should read: “Phylogenetic reconstruction of centipede interleukin-17 family domain-containing protein by ML under VT+G4 (chosen according to BIC) displayed as mid-point rooted tree. Sequences identified in venom proteomes are coloured red. Bootstrap support values are shown at each node. Note the poor support for the nesting of Cryptops within scolopendrids.”

These details have been corrected only in this corrigendum to preserve the published version of record.

