# Supplementary material for: Parallel Evolution of Complex Centipede Venoms Revealed by Comparative Proteotranscriptomic Analyses
Source: Mol Biol Evol. 2019 Aug 8;36(12):2748–63. doi: 10.1093/molbev/msz181 (PMC6878950; doi:10.1093/molbev/msz181)
Supplement: msz181_Supplementary_Data [file msz181_supplementary_data.zip › Supplementary_Figures.pdf]

## Supplementary Figure S1

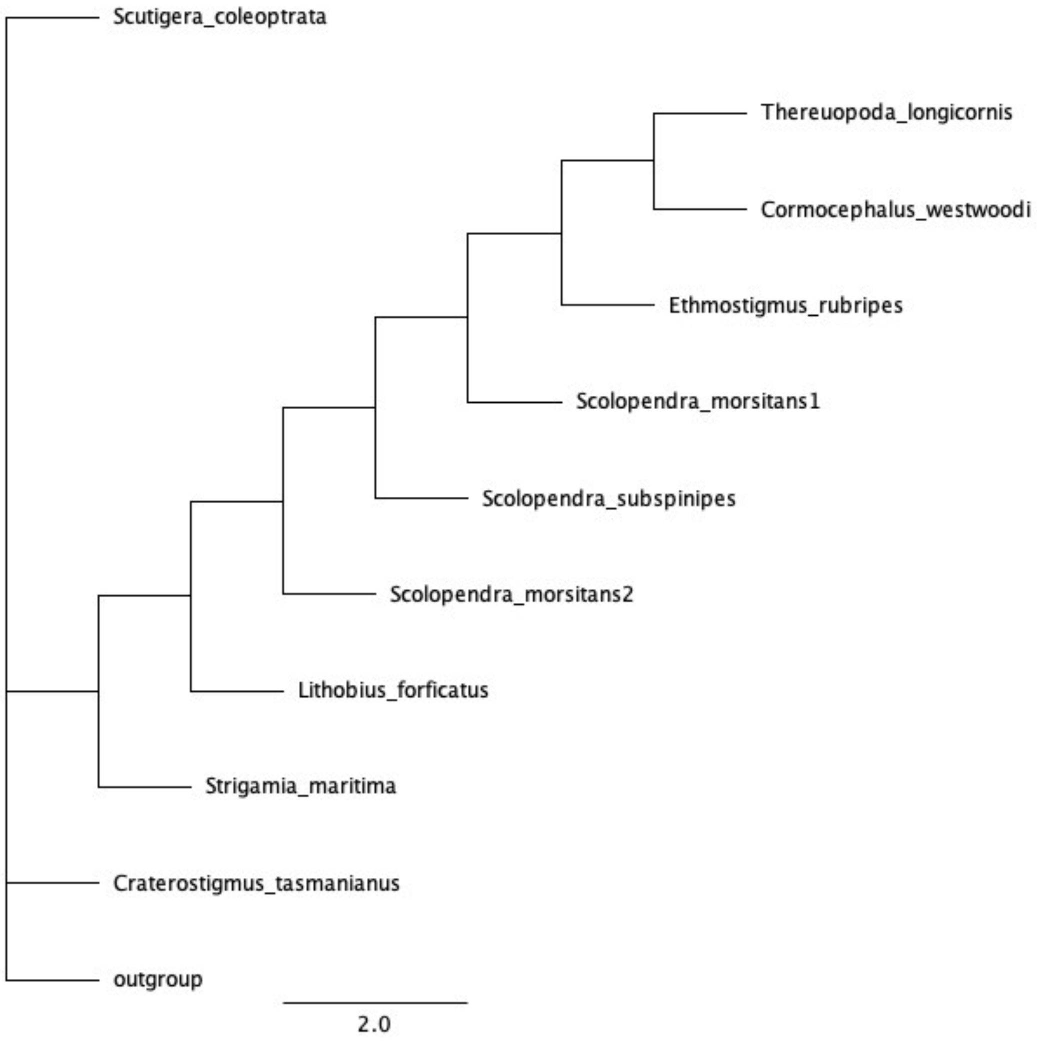


**Supplementary Figure S1**: Strict consensus tree summarizing the two most parsimonious trees resulting from a Branch and Bound parsimony analysis of the absence/presence dataset of toxin families identified in the venom gland transcriptomes (Supplementary Materials S2). Scolopendra_morsitans1 represents the transcriptome sequenced with Roche 454 technology, while Scolopendra_morsitans2 represents the transcriptome sequenced with Illumina NextSeq technology.

## Supplementary Figure S2

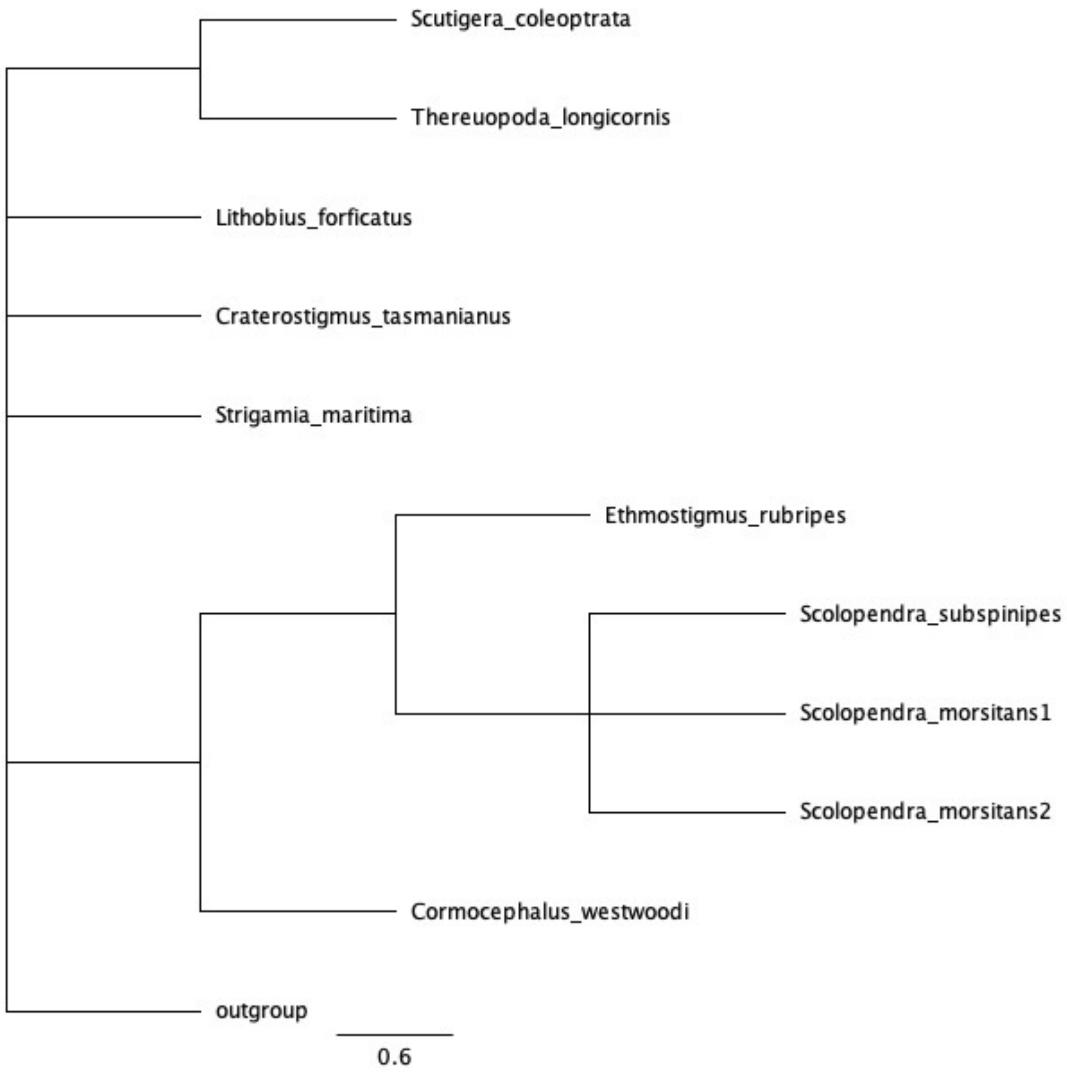


Figure S2: Strict consensus tree summarizing the nine most parsimonious trees resulting from a Branch and Bound parsimony analysis of the absence/presence dataset of toxin families identified in the venom proteomes (Supplementary Materials S5). Scolopendra_morsitans1 represents the transcriptome sequenced with Roche 454 technology, while Scolopendra_morsitans2 represents the transcriptome sequenced with Illumina NextSeq technology.

## Supplementary Figure S3

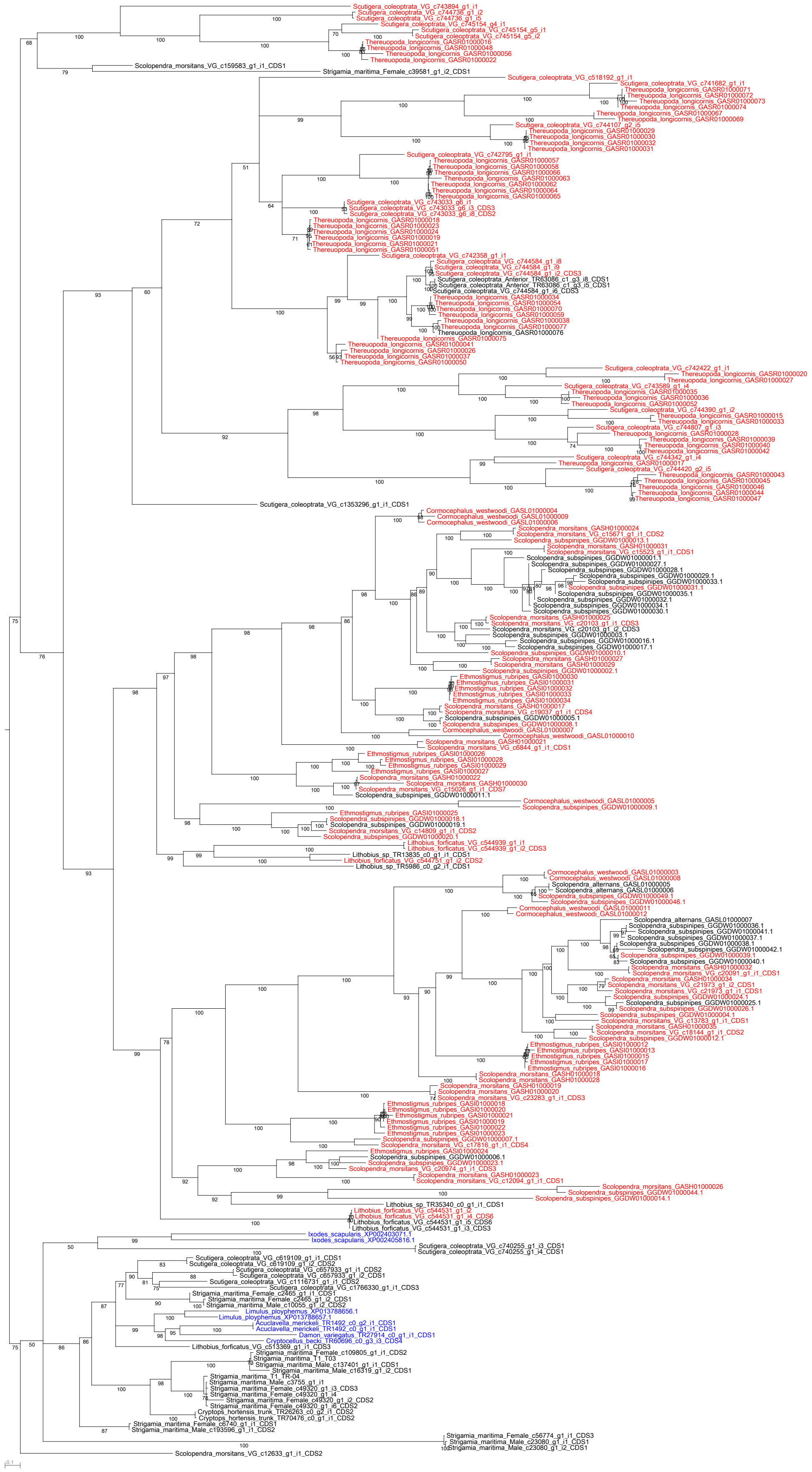

**Supplementary Figure S3:** ML phylogenetic reconstruction of the BPFTx family (under WAG+F+R5, chosen according to BIC) displayed as rooted with clade containing taxonomic outgroup sequences. Sequences identified in venom proteomes are coloured red, while non-chilopod sequences are coloured blue. Bootstrap support values are shown at each node, and nodes with support < 50 are collapsed.

## Supplementary Figure S4

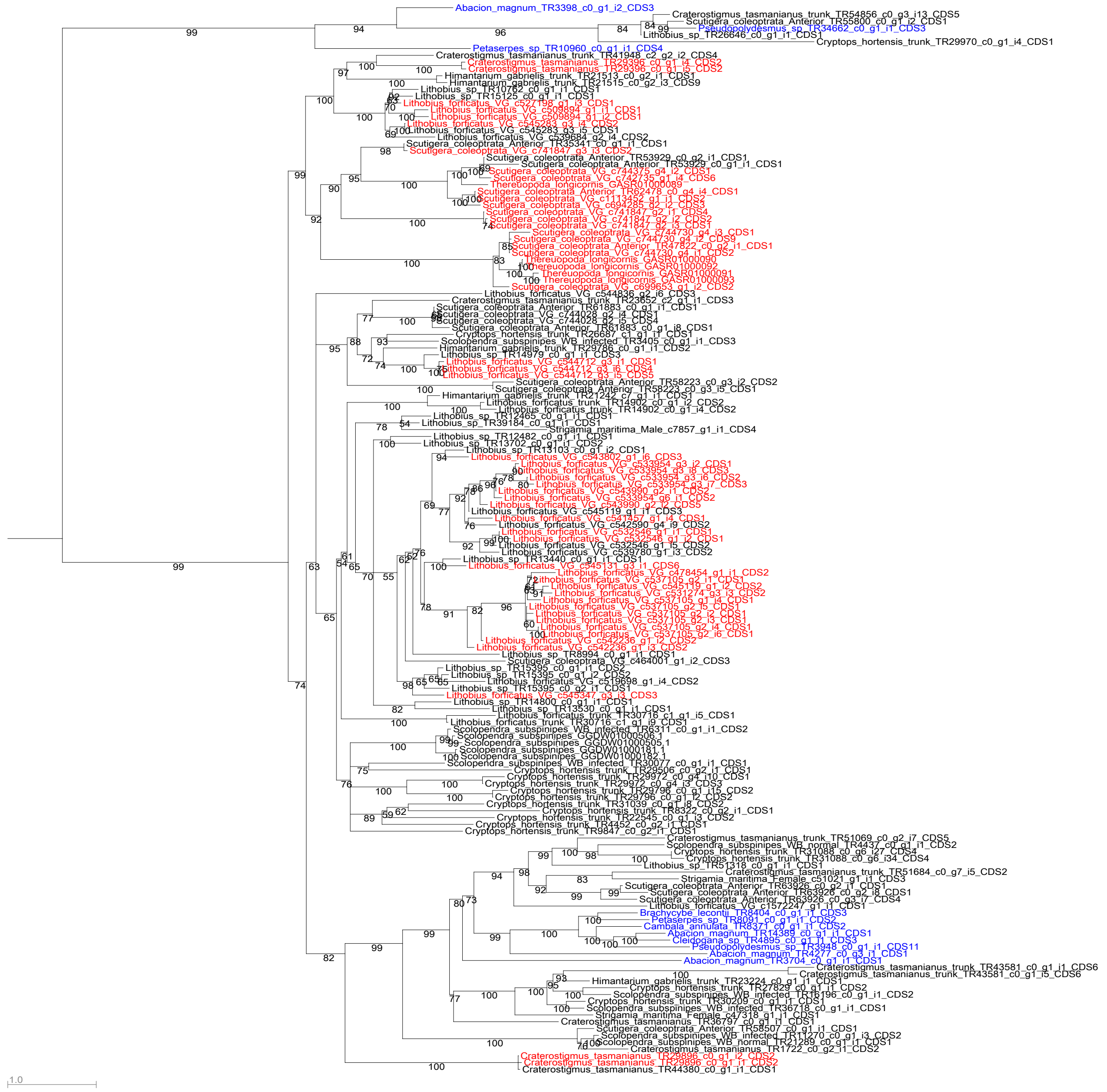

**Supplementary Figure S4:** Phylogenetic reconstruction of the myriapod M12A protein family by ML under WAG+F+R7 (chosen according to BIC) displayed as mid-point rooted tree. Sequences identified in venom proteomes are coloured red, while non-chilopod sequences are coloured blue. Bootstrap support values are shown at each node, and nodes with support < 50 are collapsed into multifurcations.

## Supplementary Figure S5

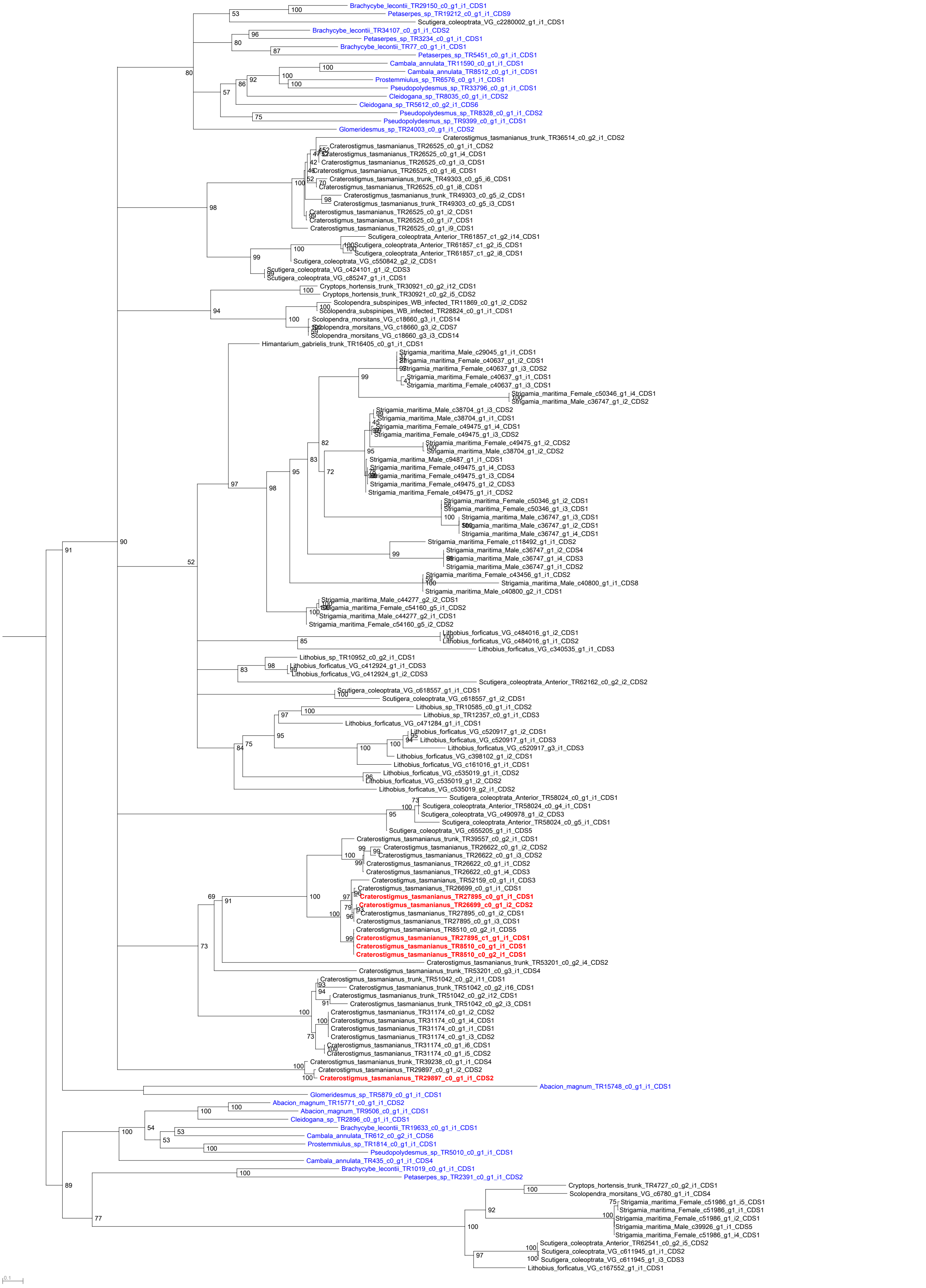


**Supplementary Figure S5:** Phylogenetic reconstruction of the myriapod acid phosphatase protein family by ML under WAG+F+R4 (chosen according to BIC) displayed as mid-point rooted tree. Sequences identified in venom proteomes are coloured red, while non-chilopod sequences are coloured blue. Bootstrap support values are shown at each node, and nodes with support < 50 are collapsed into multifurcations.

## Supplementary Figure S6

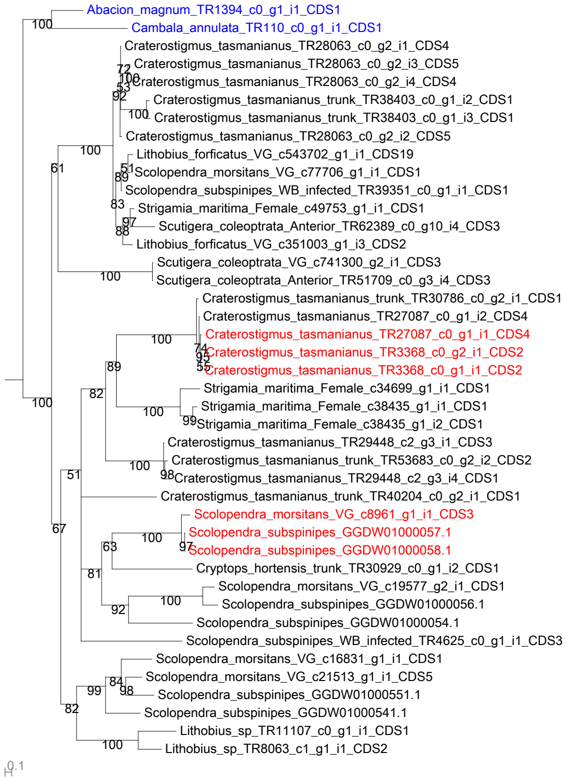


**Supplementary Figure S6:** Phylogenetic reconstruction of the myriapod C-type lectin protein family by ML under VT+R3 (chosen according to BIC) displayed as mid-point rooted tree. Sequences identified in venom proteomes are coloured red, while non-chilopod sequences are coloured blue. Bootstrap support values are shown at each node, and nodes with support < 50 are collapsed into multifurcations.

## Supplementary Figure S7

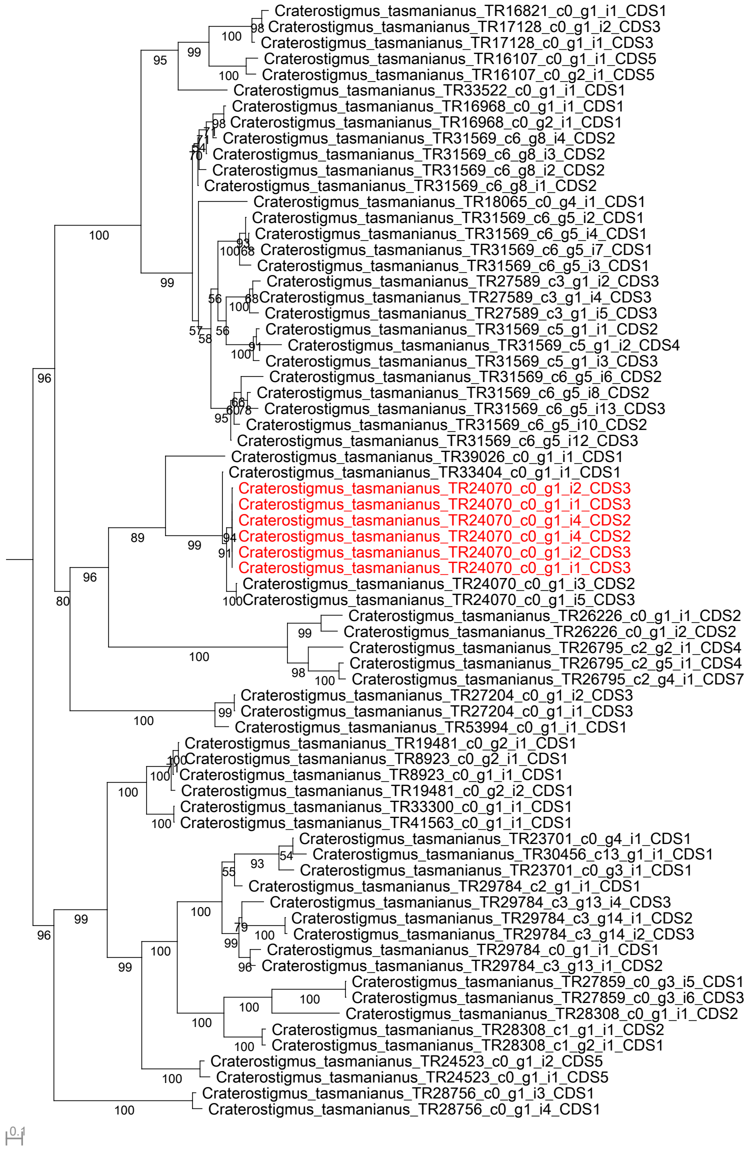


**Supplementary Figure S7:** Phylogenetic reconstruction of the myriapod calycin/lipocalin protein family by ML under WAG+I+G4 (chosen according to BIC) displayed as mid-point rooted tree. Sequences identified in venom proteomes are coloured red. Bootstrap support values are shown at each node, and nodes with support < 50 are collapsed into multifurcations.

## Supplementary Figure S8

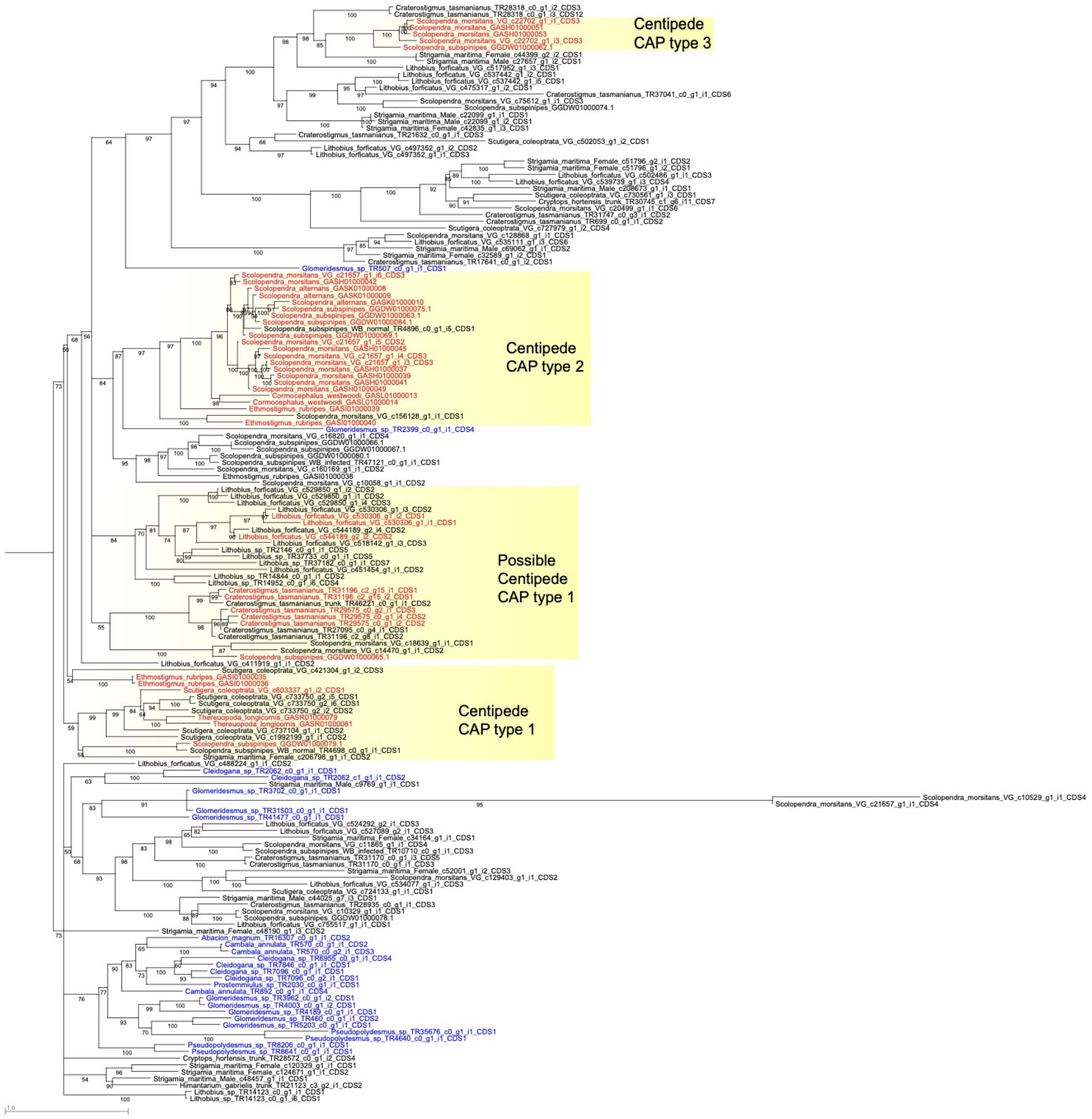


**Supplementary Figure S8:** Phylogenetic reconstruction of the myriapod CAP protein family by ML under WAG+R5 (chosen according to BIC) displayed as mid-point rooted tree. Sequences identified in venom proteomes are coloured red, while non-chilopod sequences are coloured blue. Clades belonging to one of the three centipede venom types are labelled and highlighted in yellow. Bootstrap support values are shown at each node, and nodes with support < 50 are collapsed into multifurcations.

## Supplementary Figure S9

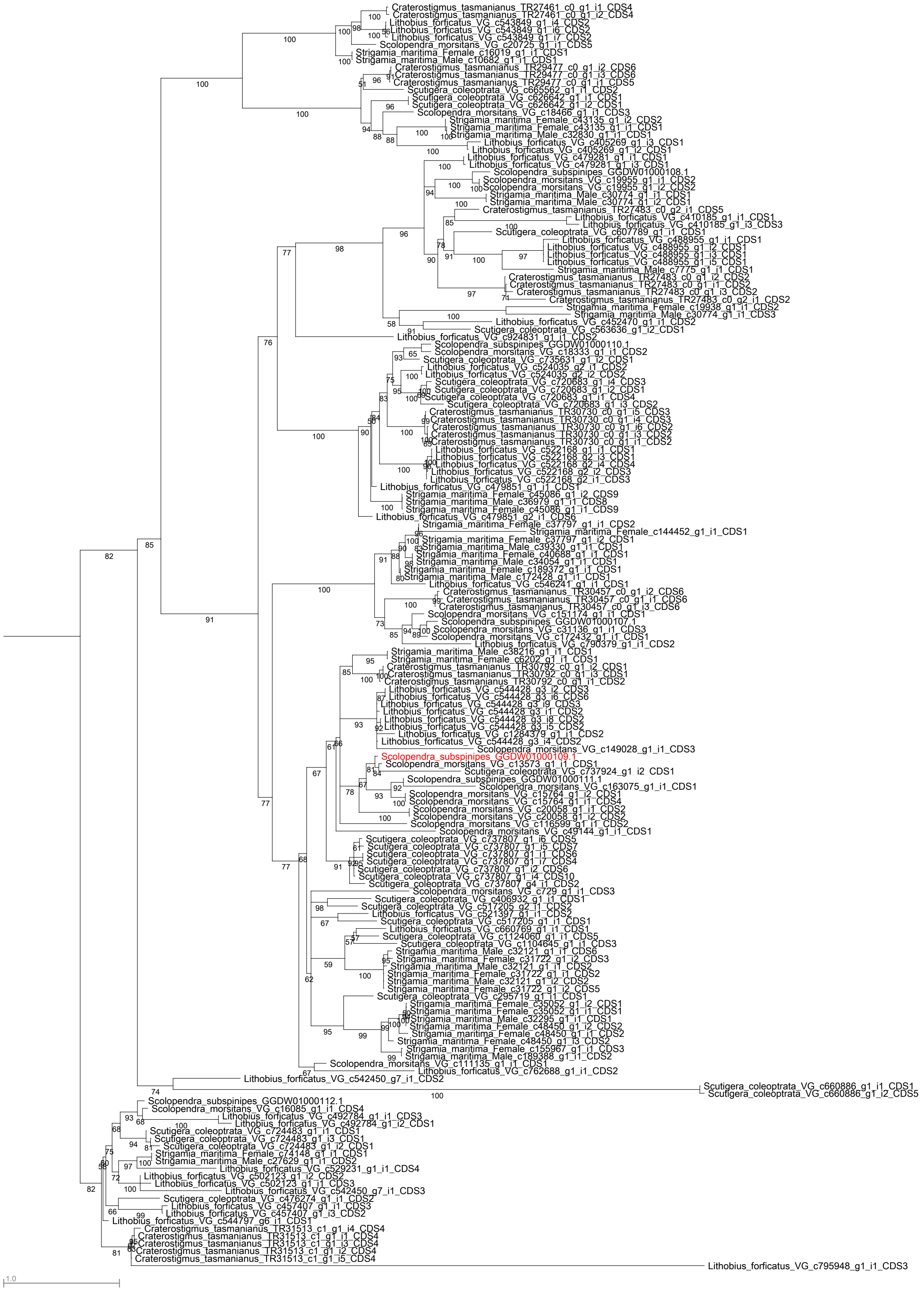

**Supplementary Figure S9:** Phylogenetic reconstruction of the centipede cathepsin protein family by ML under WAG+R4 (chosen according to BIC) displayed as mid-point rooted tree. Sequences identified in venom proteomes are coloured red. Bootstrap support values are shown at each node, and nodes with support < 50 are collapsed into multifurcations.

## Supplementary Figure S10

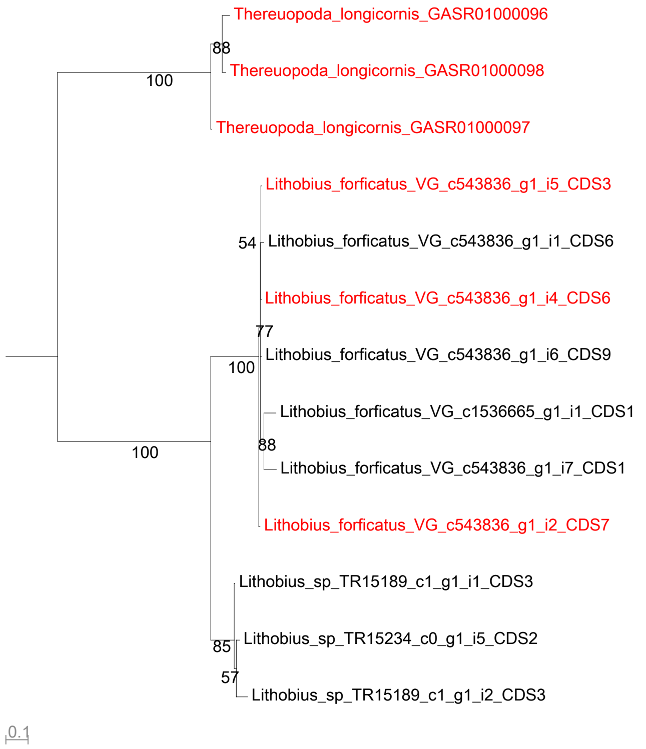


**Supplementary Figure S10:** Phylogenetic reconstruction of the centipede peptidyl arginine deiminase protein family by ML under WAG (chosen according to BIC) displayed as mid-point rooted tree. Sequences identified in venom proteomes are coloured red. Bootstrap support values are shown at each node, and nodes with support < 50 are collapsed into multifurcations.

## Supplementary Figure S11

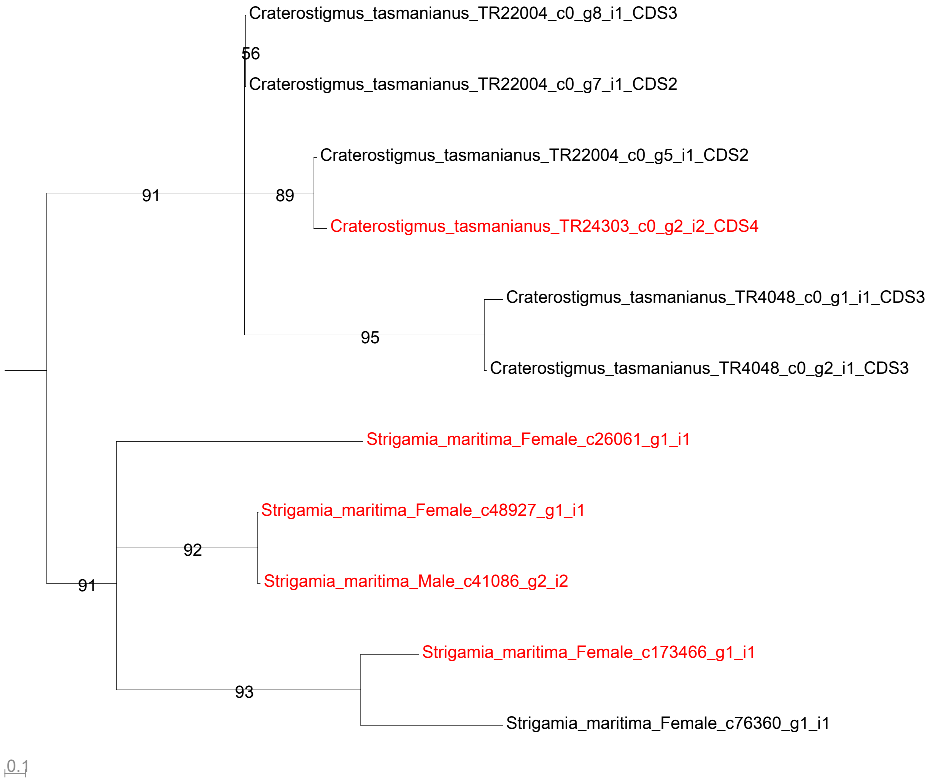


**Supplementary Figure S11:** Phylogenetic reconstruction of the chilotoxin 01 peptide family by ML under PMB+G4 (chosen according to BIC) displayed as mid-point rooted tree. Sequences identified in venom proteomes are coloured red. Bootstrap support values are shown at each node, and nodes with support < 50 are collapsed into multifurcations.

## Supplementary Figure S12

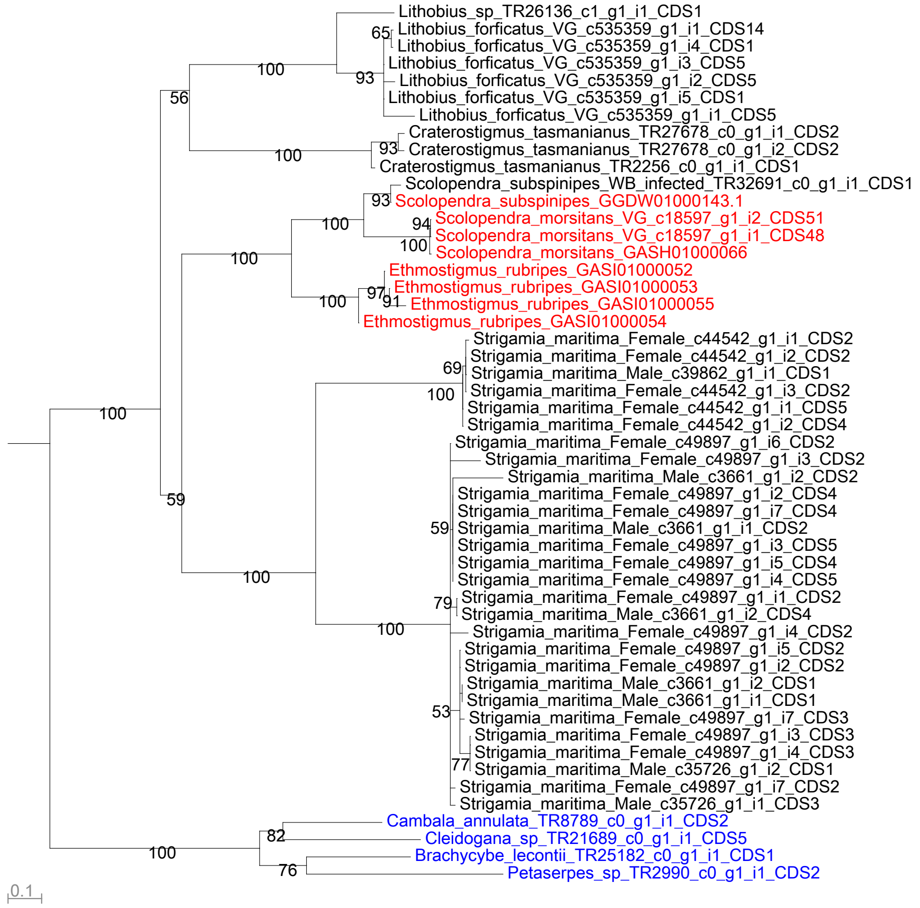


**Supplementary Figure S12:** Phylogenetic reconstruction of the myriapod chondroitinase family by ML under VT+G4 (chosen according to BIC) displayed as mid-point rooted tree. Sequences identified in venom proteomes are coloured red, while non-chilopod sequences are coloured blue. Bootstrap support values are shown at each node, and nodes with support < 50 are collapsed into multifurcations.

## Supplementary Figure S13

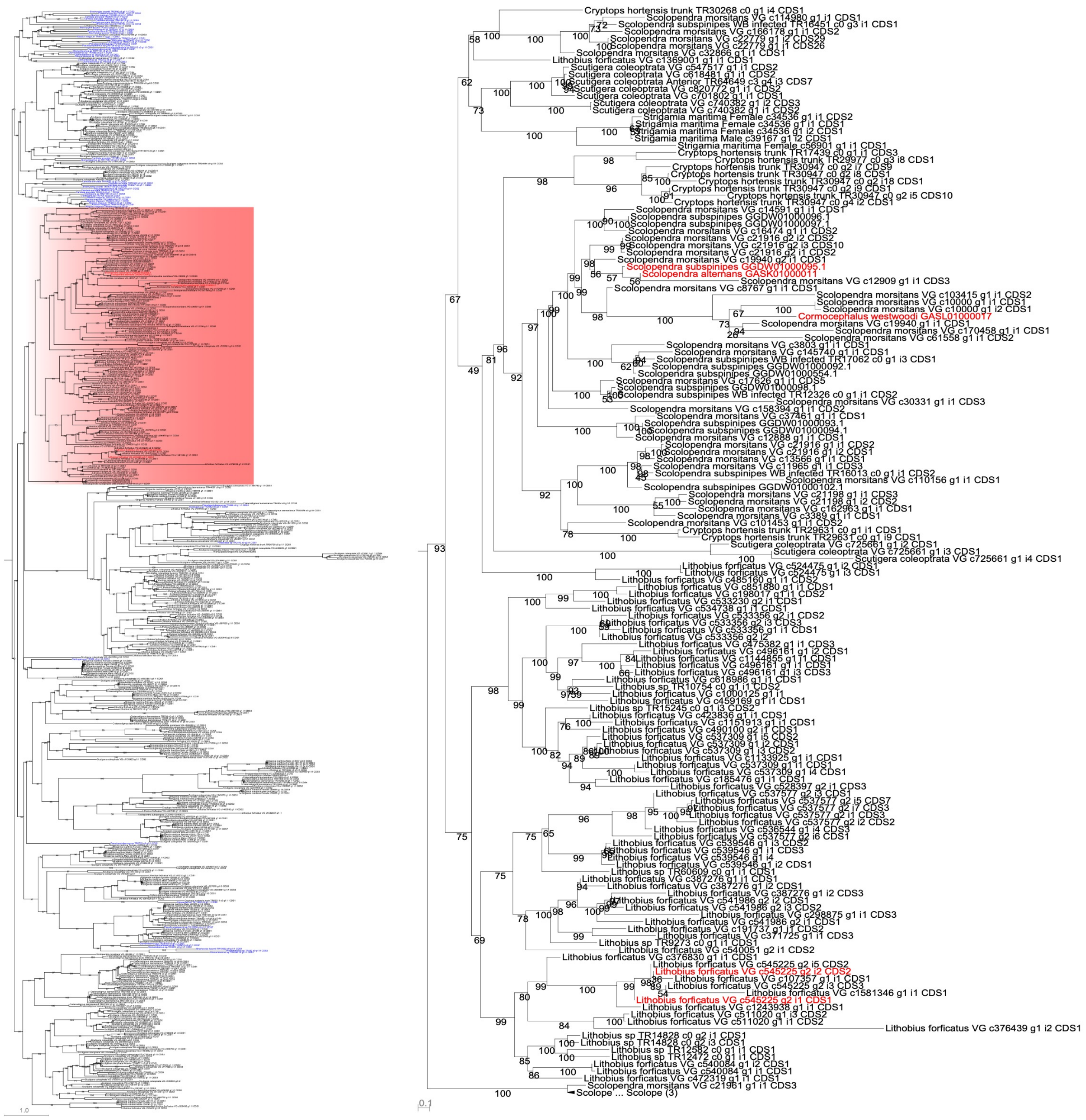

**Supplementary Figure S13:** Phylogenetic reconstruction of the myriapod carboxylesterase type B family by ML under WAG+F+R8 (chosen according to BIC) displayed as mid-point rooted tree, showing full tree (left) and subtree highlighted in red containing venom forms (right). Sequences identified in venom proteomes are coloured red, while non-chilopod sequences are coloured blue. Bootstrap support values are shown at each node, and nodes with support < 50 are collapsed into multifurcations.

## Supplementary Figure S14

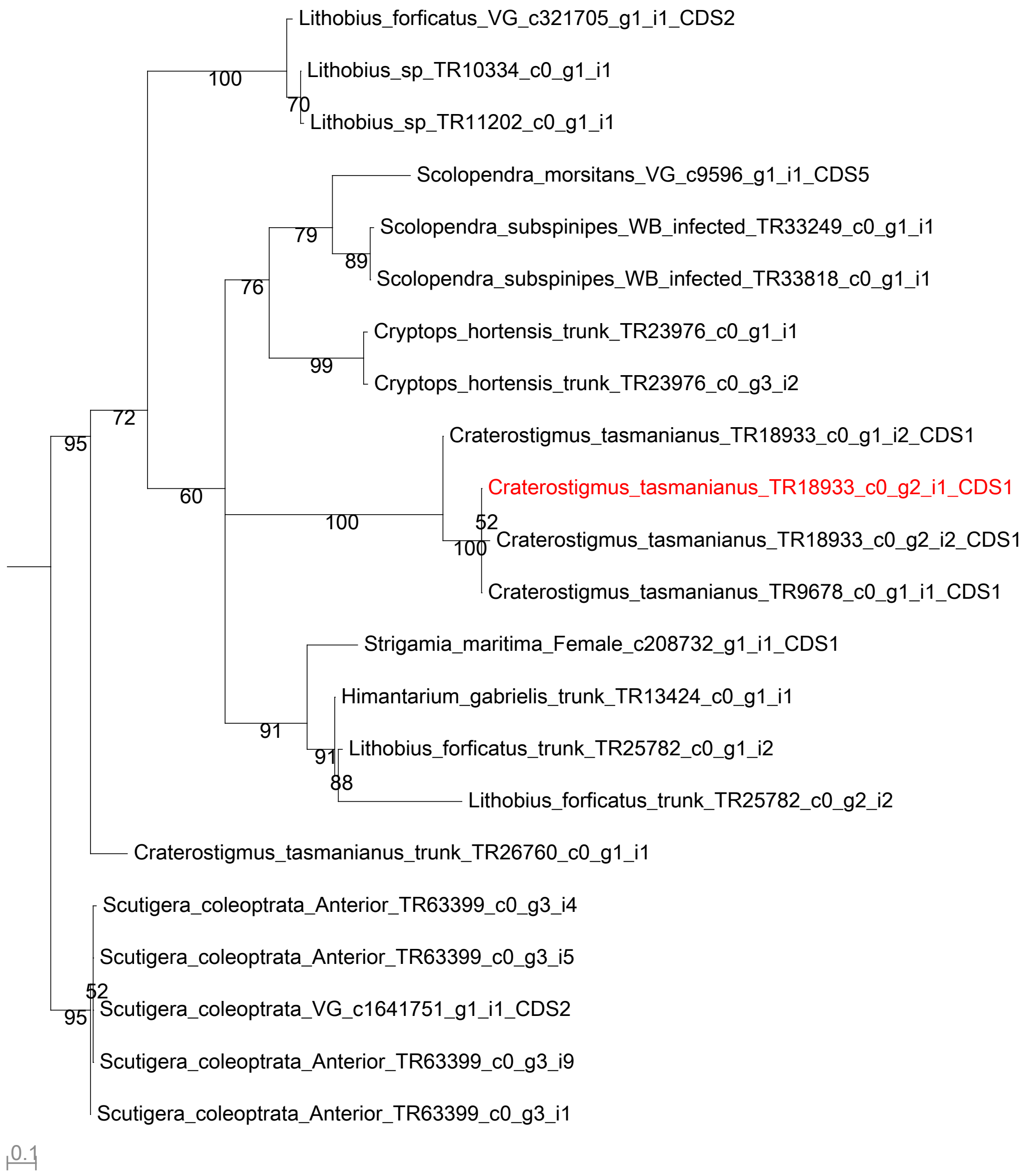


**Supplementary Figure S14:** Phylogenetic reconstruction of the CTRTX02 family by ML under PMB+G4 (chosen according to BIC) displayed as mid-point rooted tree. Sequences identified in venom proteomes are coloured red. Bootstrap support values are shown at each node, and nodes with support < 50 are collapsed into multifurcations.

## Supplementary Figure S15

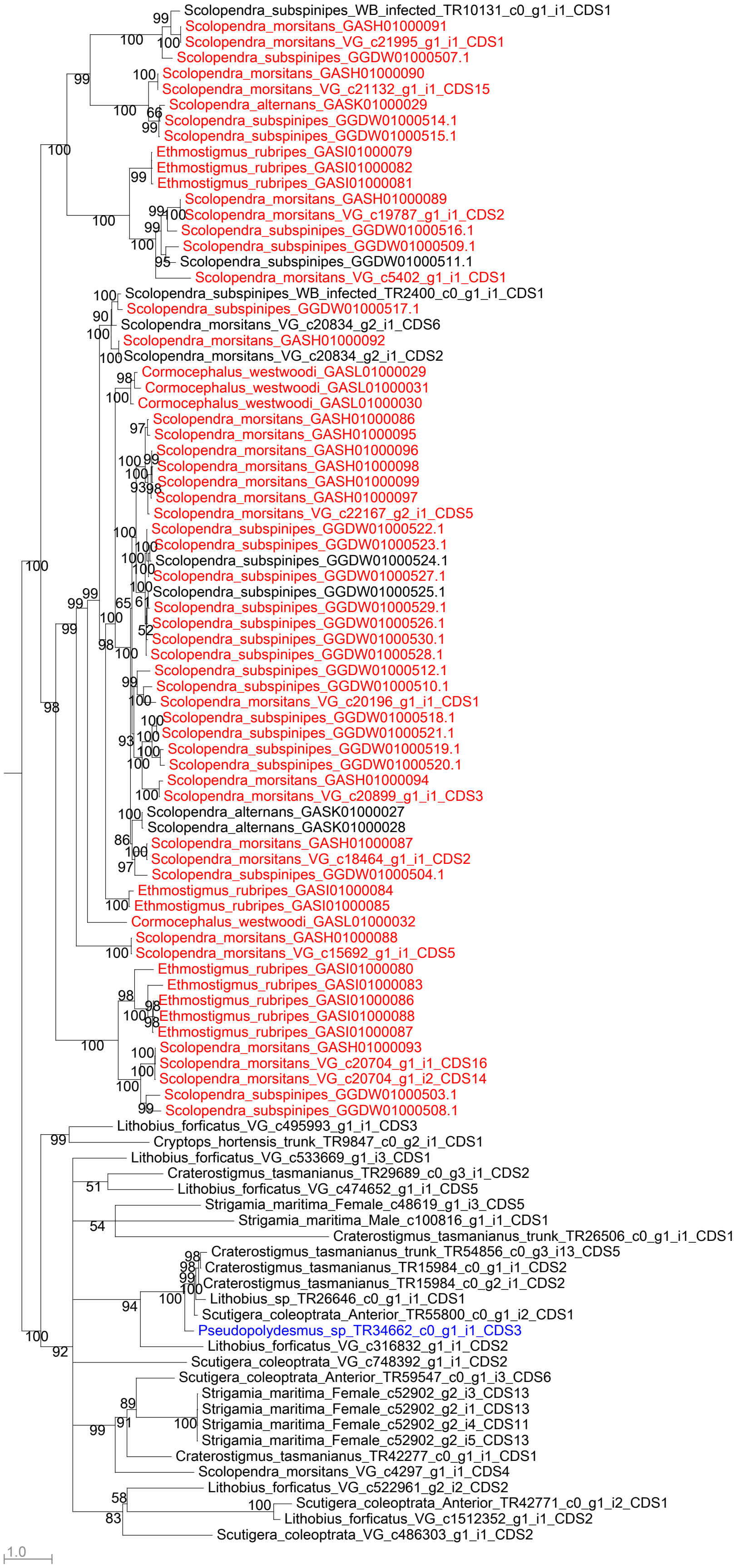


**Supplementary Figure S15:** Phylogenetic reconstruction of the myriapod CUB-domain protein family by ML under WAG+F+R4 (chosen according to BIC) displayed as mid-point rooted tree. Sequences identified in venom proteomes are coloured red, while non-chilopod sequences are coloured blue. Bootstrap support values are shown at each node, and nodes with support < 50 are collapsed into multifurcations.

## Supplementary Figure S16

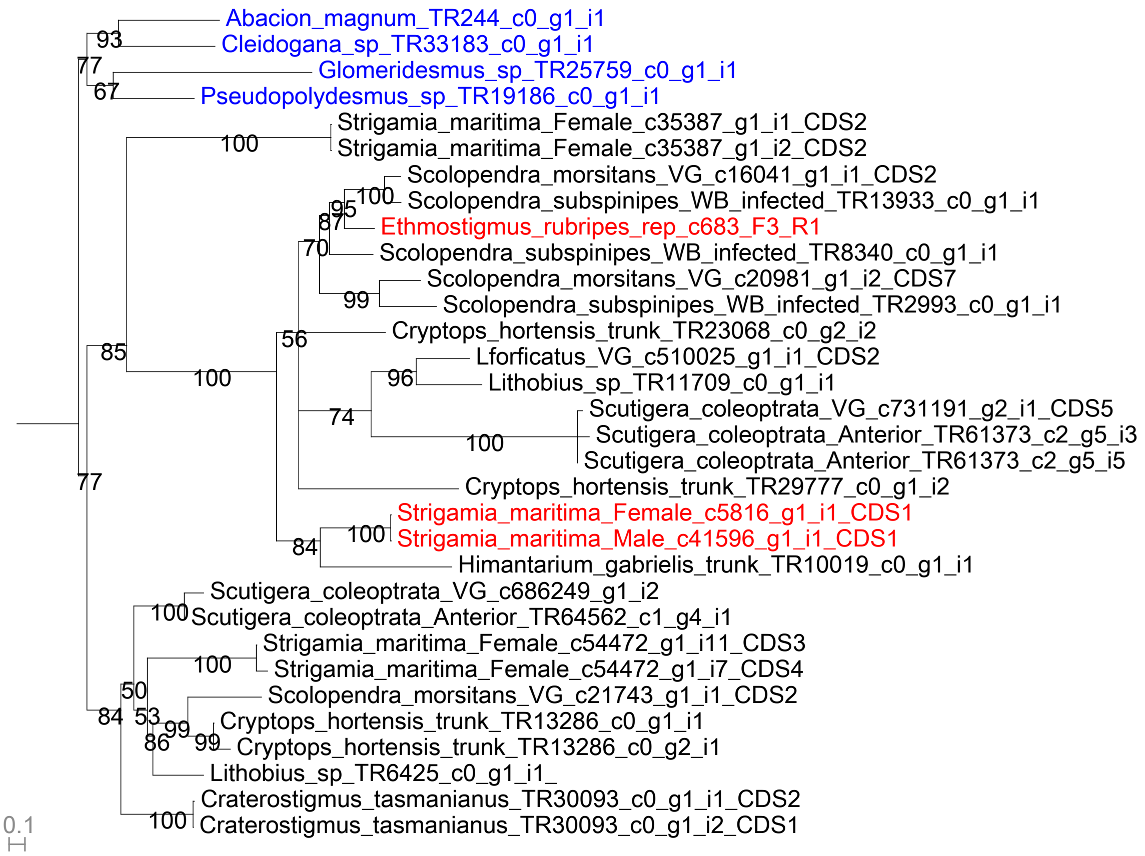


**Supplementary Figure S16:** Phylogenetic reconstruction of the myriapod cystatin family by ML under WAG+R4 (chosen according to BIC) displayed as mid-point rooted tree. Sequences identified in venom proteomes are coloured red, while non-chilopod sequences are coloured blue. Bootstrap support values are shown at each node, and nodes with support < 50 are collapsed into multifurcations.

## Supplementary Figure S17

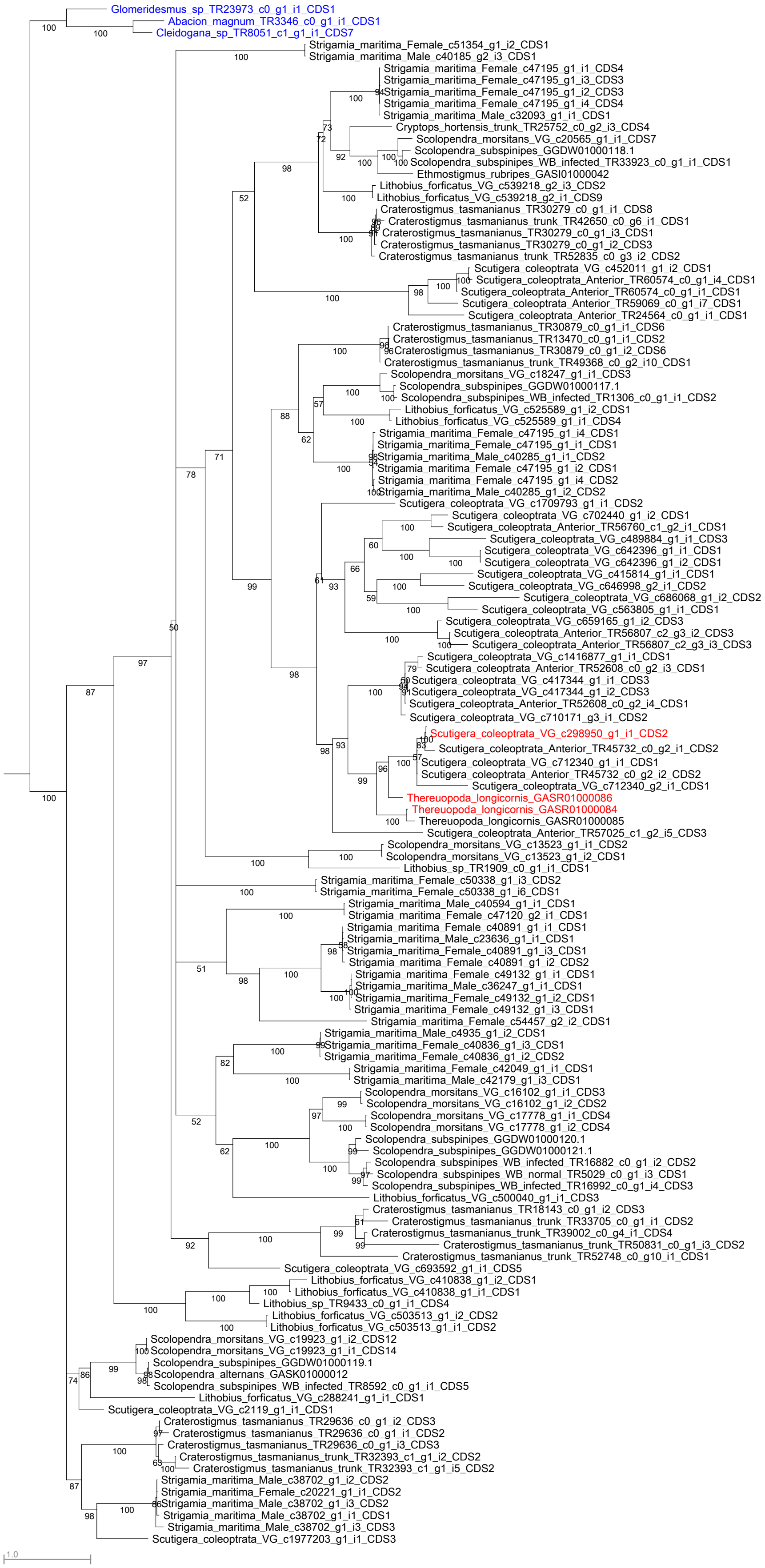

**Supplementary Figure S17:** Phylogenetic reconstruction of the myriapod DUF1397 protein family by ML under VT+R3 (chosen according to BIC) displayed as mid-point rooted tree. Sequences identified in venom proteomes are coloured red, while non-chilopod sequences are coloured blue. Bootstrap support values are shown at each node, and nodes with support < 50 are collapsed into multifurcations.

# Supplementary Figure S18

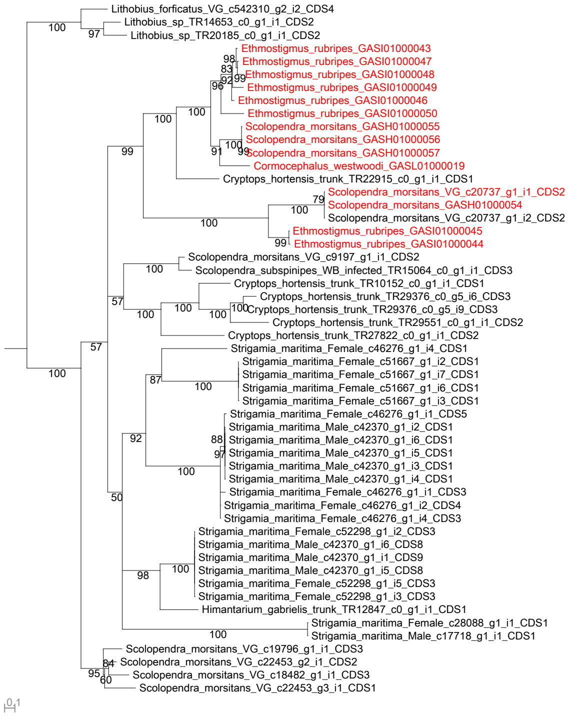


**Supplementary Figure S18:** Phylogenetic reconstruction of the myriapod DUF3472 protein family by ML under VT+G4 (chosen according to BIC) displayed as mid-point rooted tree. Sequences identified in venom proteomes are coloured red. Bootstrap support values are shown at each node, and nodes with support < 50 are collapsed into multifurcations.

# Supplementary Figure S19

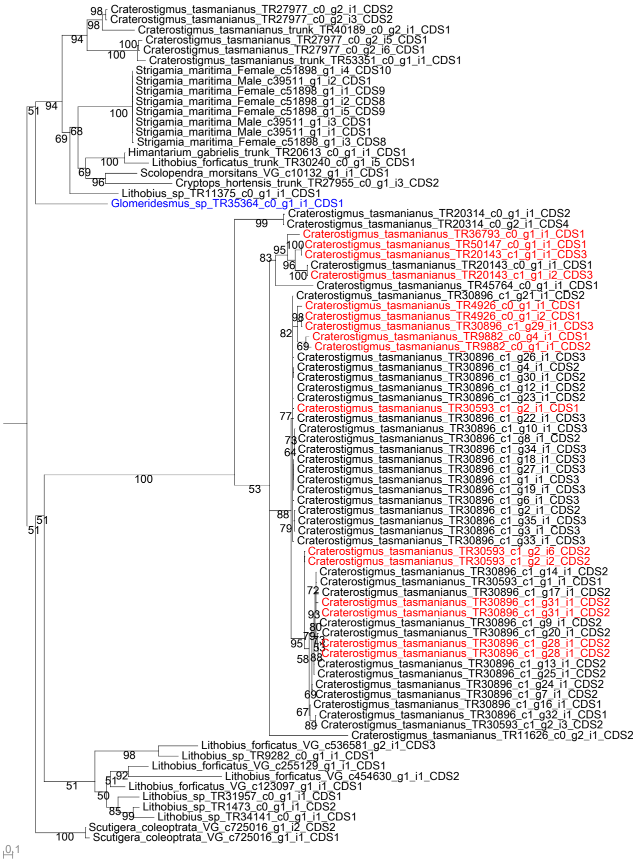

**Supplementary Figure S19:** Phylogenetic reconstruction of the myriapod DUF4773 protein family by ML under VT+I+G4 (chosen according to BIC) displayed as mid-point rooted tree. Sequences identified in venom proteomes are coloured red, while non-chilopod sequences are coloured blue. Bootstrap support values are shown at each node, and nodes with support < 50 are collapsed into multifurcations.

# Supplementary Figure S20

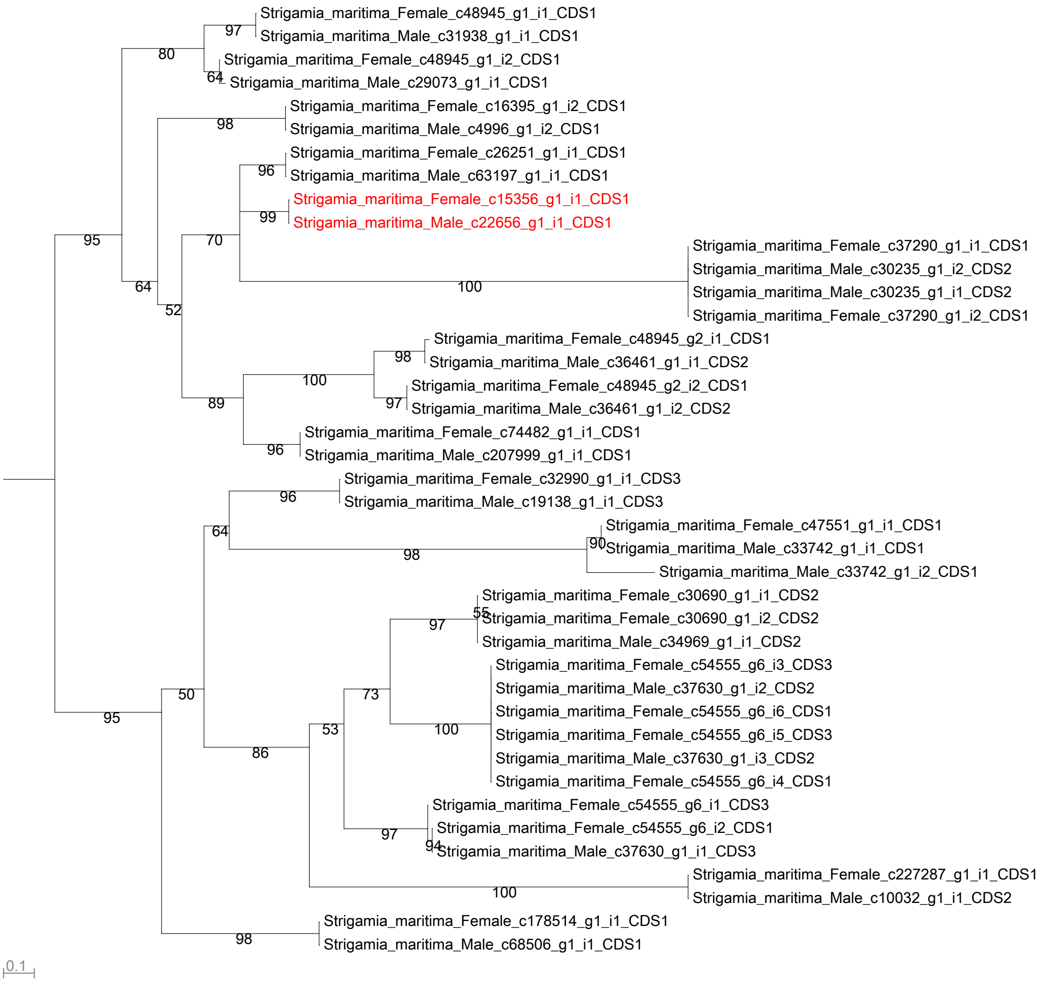


**Supplementary Figure S20:** Phylogenetic reconstruction of the GEOTX02 peptide family by ML under Blosum62+I+G4 (chosen according to BIC) displayed as mid-point rooted tree. Sequences identified in venom proteomes are coloured red. Bootstrap support values are shown at each node, and nodes with support < 50 are collapsed into multifurcations.

## Supplementary Figure S21

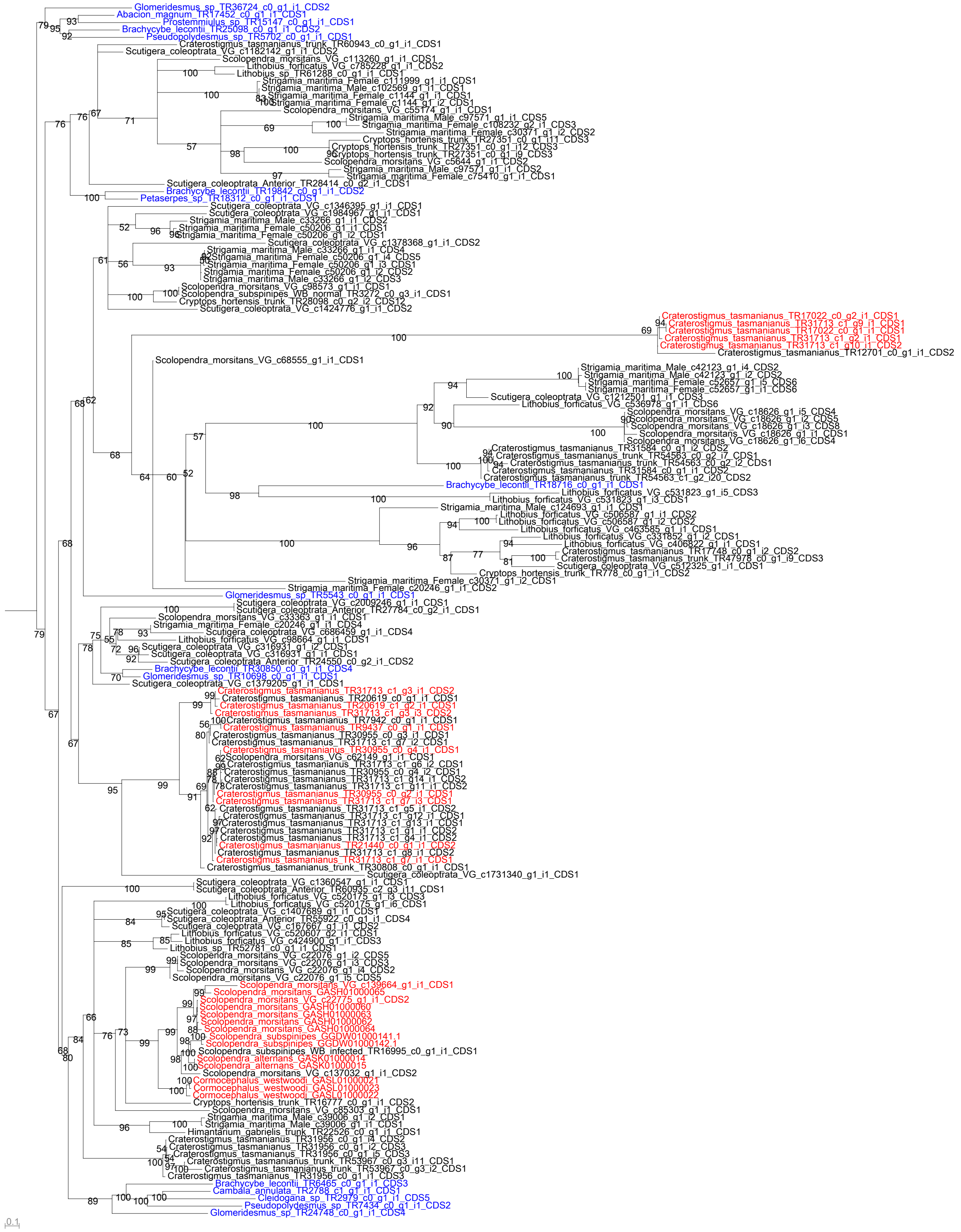

**Supplementary Figure S21:** Phylogenetic reconstruction of the myriapod gamma-glutamyl transpeptidase family by ML under VT+R4 (chosen according to BIC) displayed as mid-point rooted tree. Sequences identified in venom proteomes are coloured red, while non-chilopod sequences are coloured blue. Bootstrap support values are shown at each node, and nodes with support < 50 are collapsed into multifurcations.

## Supplementary Figure S22

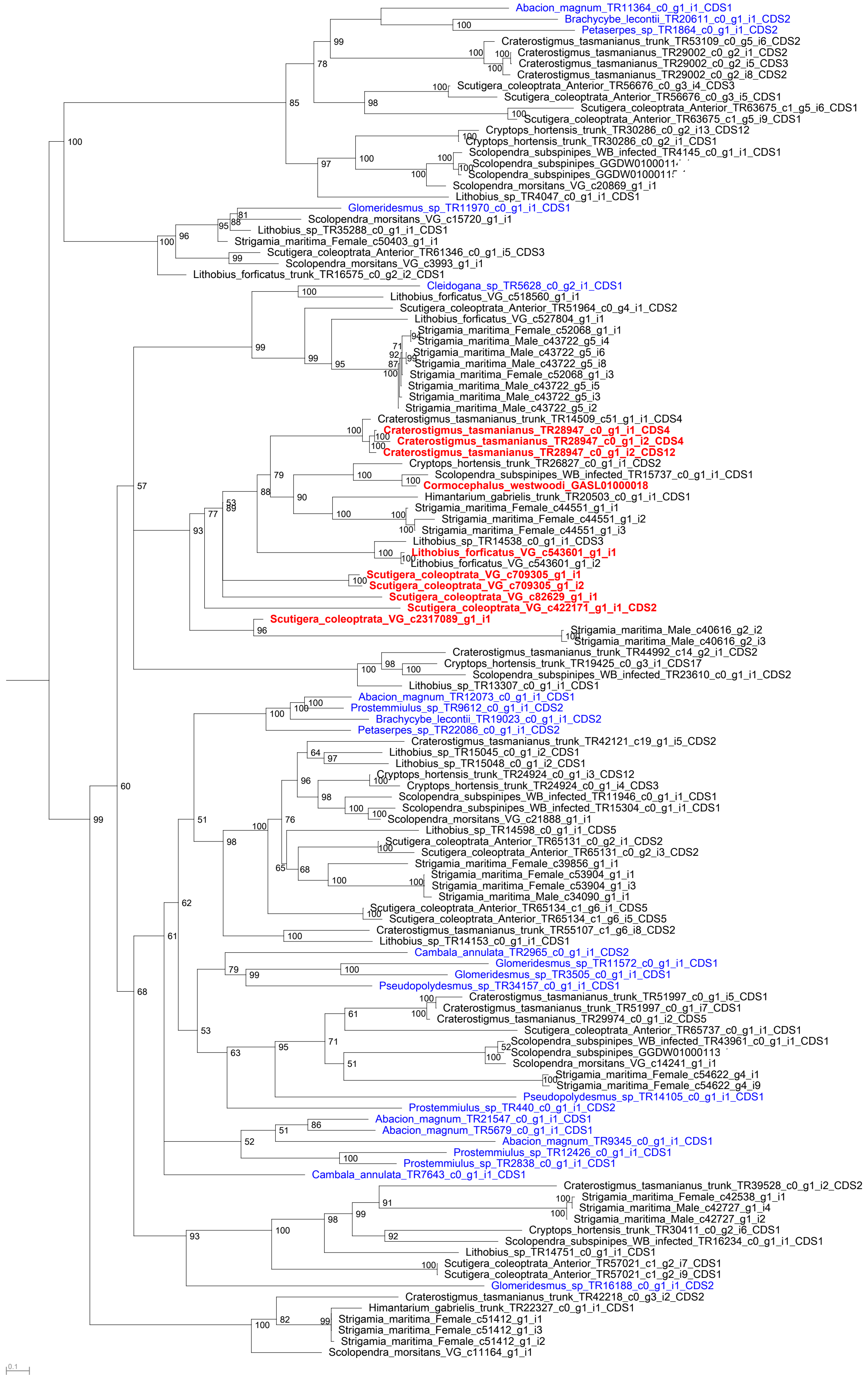

**Supplementary Figure S22:** Phylogenetic reconstruction of the myriapod glycoside hydrolase family 18 by ML under WAG+R4 (chosen according to BIC) displayed as mid-point rooted tree. Sequences identified in venom proteomes are coloured red, while non-chilopod sequences are coloured blue. Bootstrap support values are shown at each node, and nodes with support < 50 are collapsed into multifurcations.

## Supplementary Figure S23

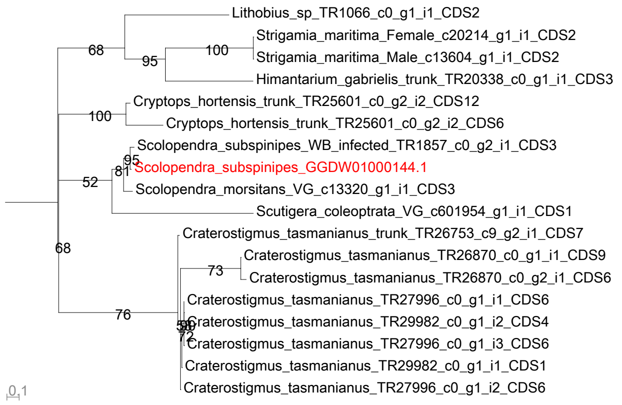


**Supplementary Figure S23:** Phylogenetic reconstruction of the centipede icarapin-like family by ML under VT+I+G4 (chosen according to BIC) displayed as mid-point rooted tree. Sequences identified in venom proteomes are coloured red. Bootstrap support values are shown at each node, and nodes with support < 50 are collapsed into multifurcations.

## Supplementary Figure S24

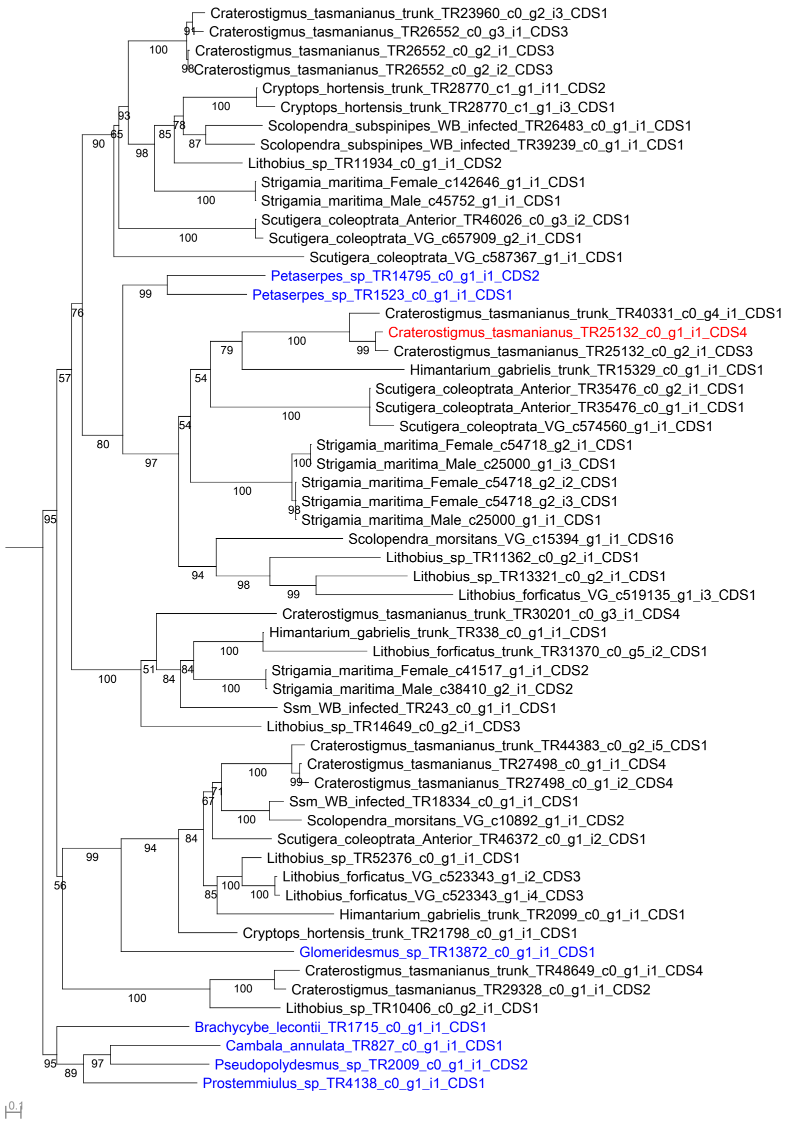


**Supplementary Figure S24:** Phylogenetic reconstruction of the myriapod IgE-ESP-like family by ML under WAG+F+I+G4 (chosen according to BIC) displayed as mid-point rooted tree. Sequences identified in venom proteomes are coloured red, while non-chilopod sequences are coloured blue. Bootstrap support values are shown at each node, and nodes with support < 50 are collapsed into multifurcations.

## Supplementary Figure S25

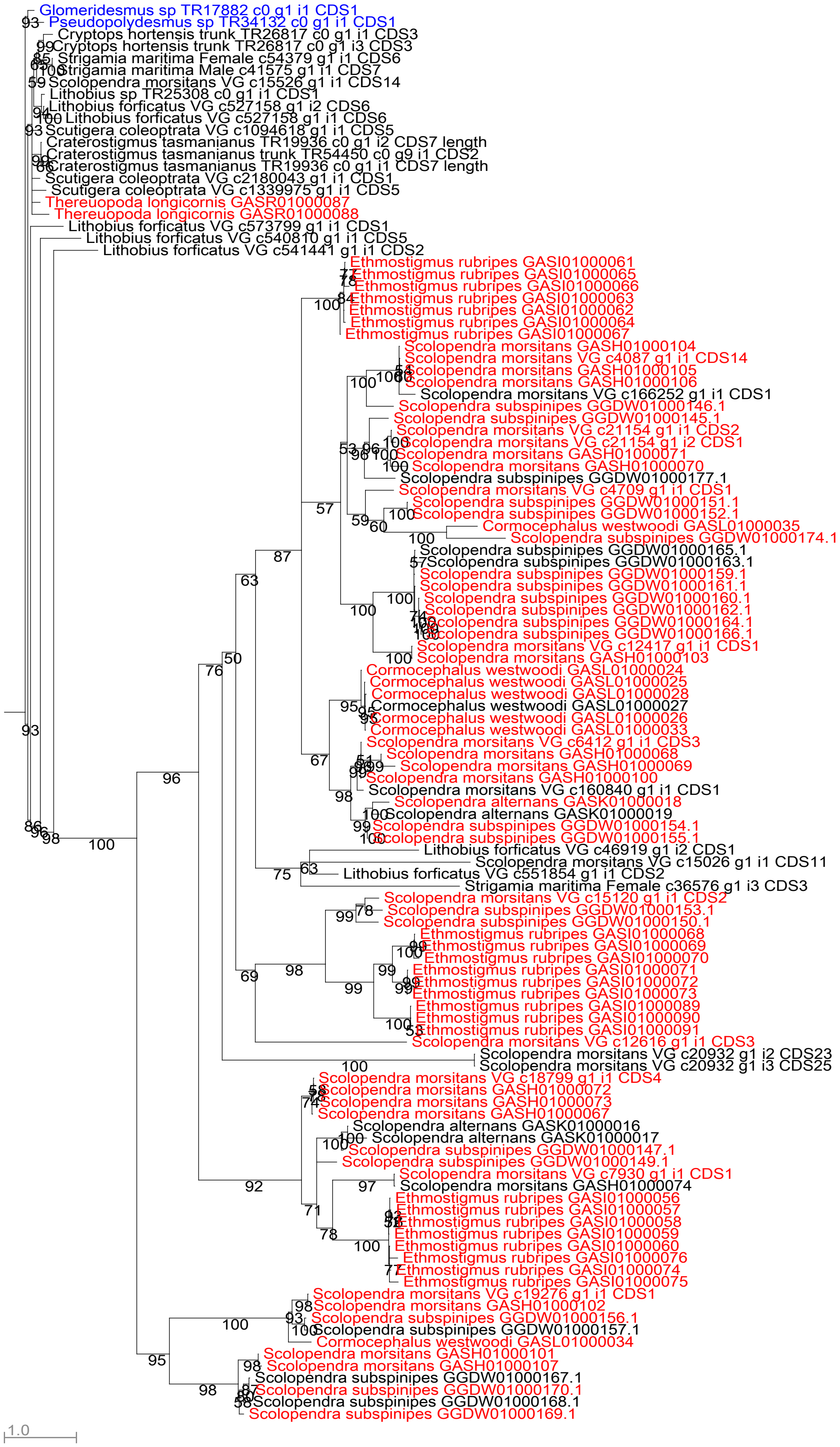

**Supplementary Figure S25:** Phylogenetic reconstruction of the myriapod LDLA-domain containing family by ML under VT+R4 (chosen according to BIC) displayed as mid-point rooted tree. Sequences identified in venom proteomes are coloured red, while non-chilopod sequences are coloured blue. Bootstrap support values are shown at each node, and nodes with support < 50 are collapsed into multifurcations.

## Supplementary Figure S26

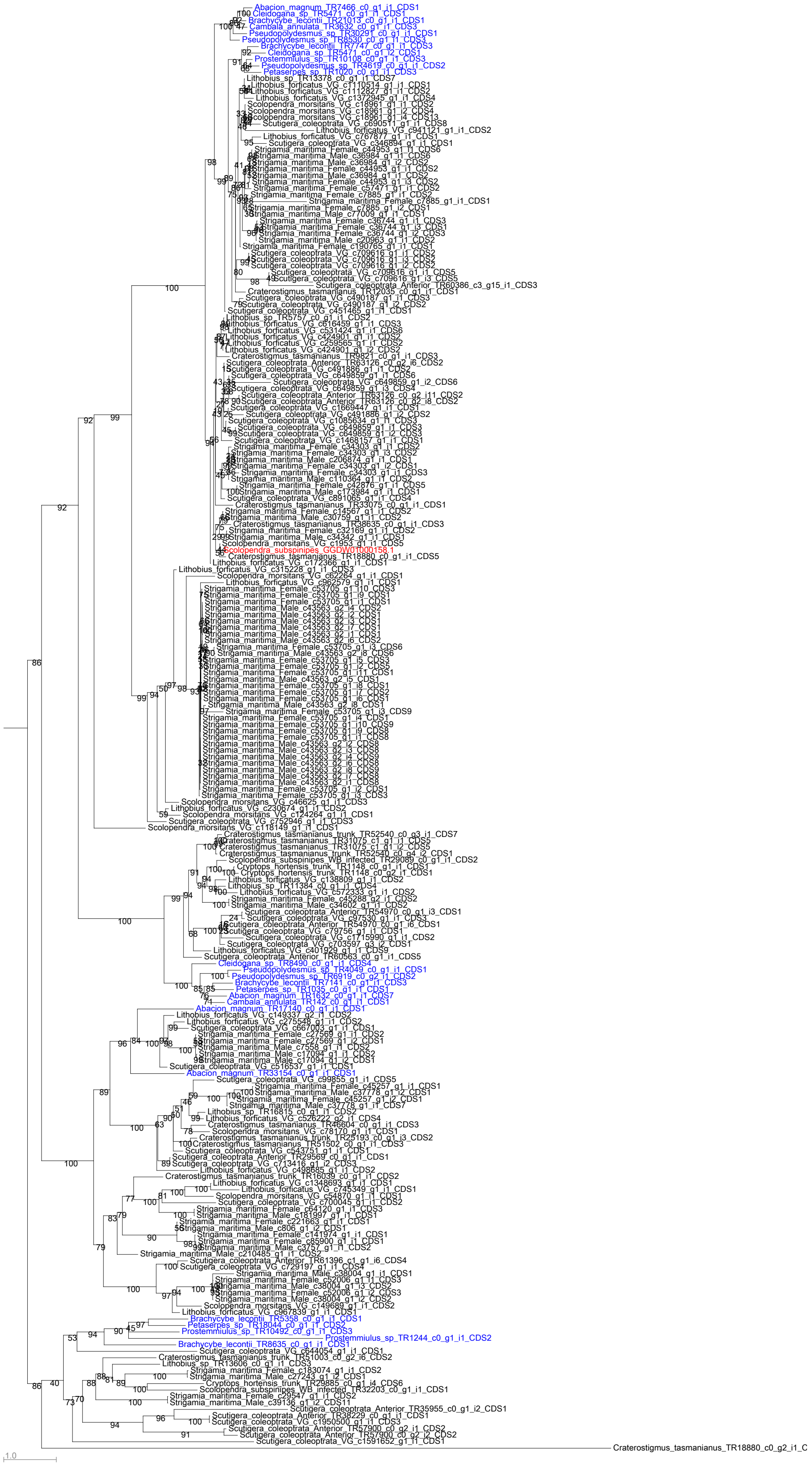

**Supplementary Figure S26:** Phylogenetic reconstruction of the myriapod LDLA-chitinase family by ML under VT+R5 (chosen according to BIC) displayed as mid-point rooted tree. Sequences identified in venom proteomes are coloured red, while non-chilopod sequences are coloured blue. Bootstrap support values are shown at each node, and nodes with support < 50 are collapsed into multifurcations.

## Supplementary Figure S27

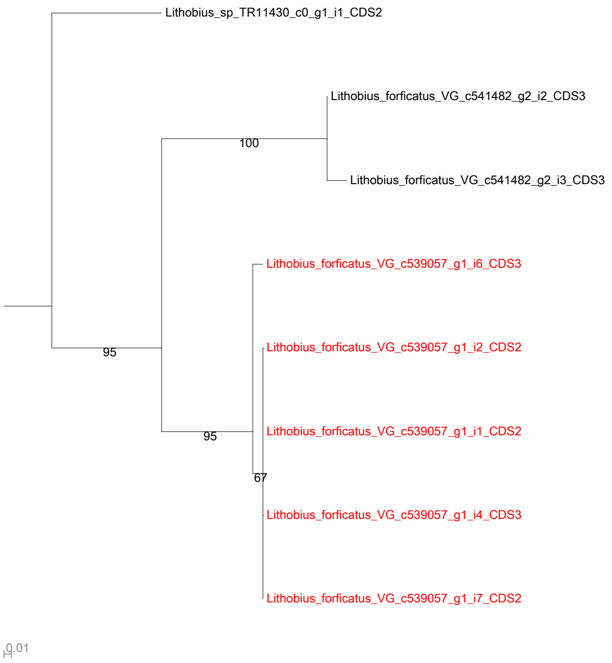


**Supplementary Figure S27:** Phylogenetic reconstruction of the LTHTX01 peptide family by ML under PMB (chosen according to BIC) displayed as mid-point rooted tree. Sequences identified in venom proteomes are coloured red. Bootstrap support values are shown at each node, and nodes with support < 50 are collapsed into multifurcations.

## Supplementary Figure S28

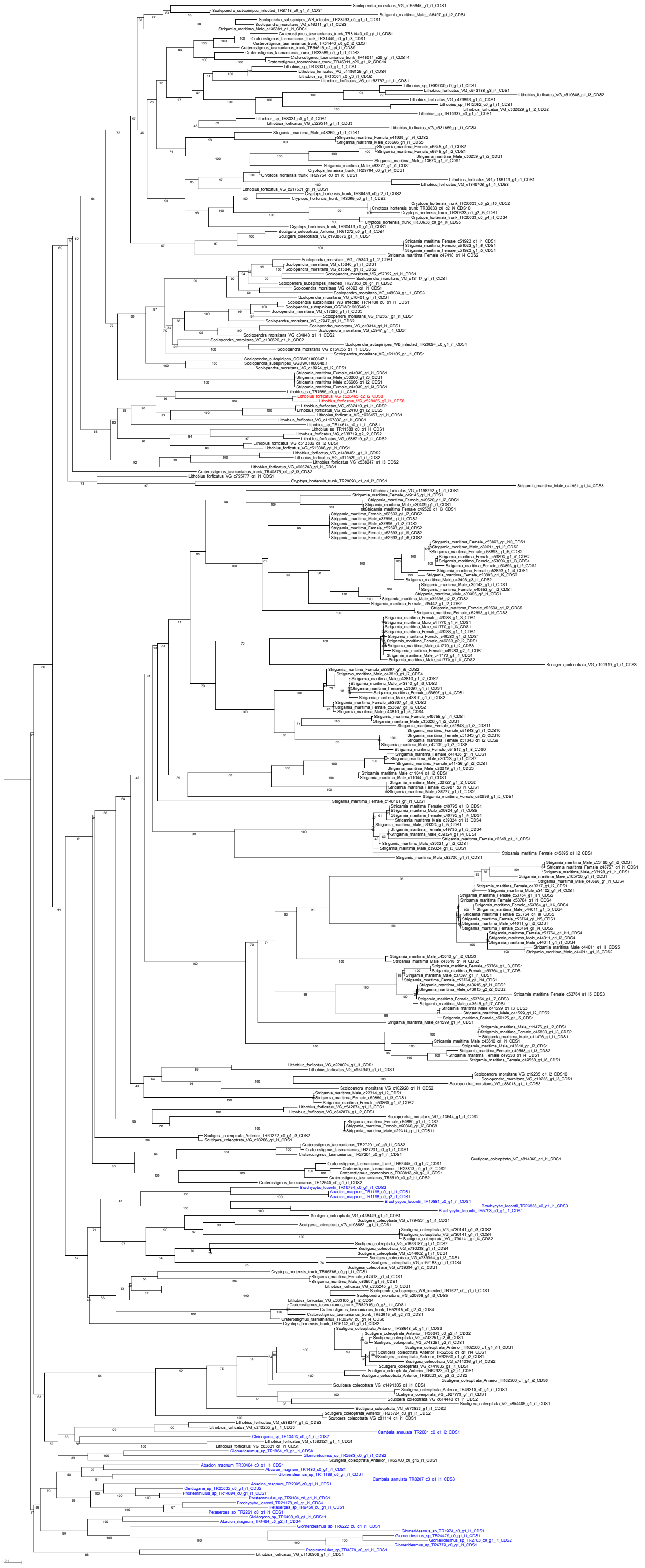

**Supplementary Figure S28:** Phylogenetic reconstruction of the myriapod lysosomal lipase family by ML under VT+F+R7 (chosen according to BIC) displayed as mid-point rooted tree. Sequences identified in venom proteomes are coloured red, while non-chilopod sequences are coloured blue. Bootstrap support values are shown at each node, and nodes with support < 50 are collapsed into multifurcations.

## Supplementary Figure S29

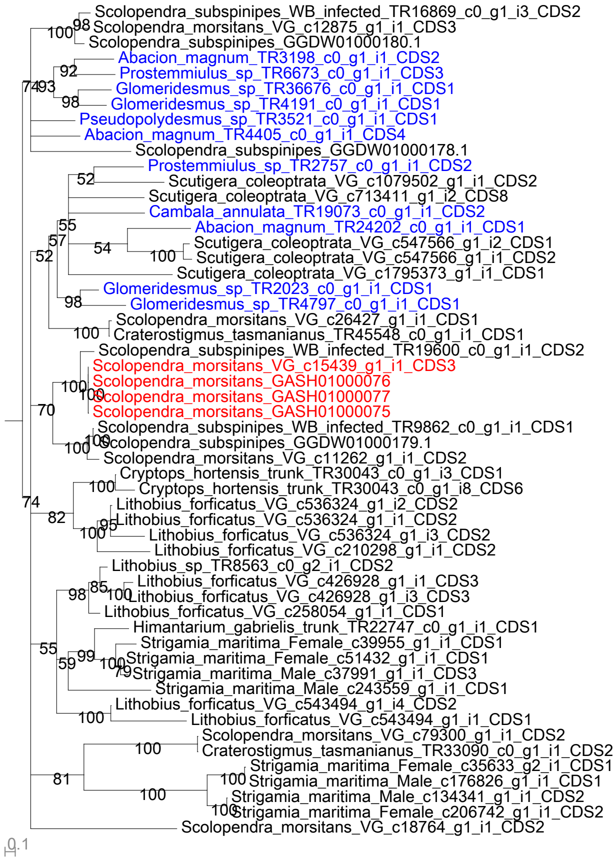


**Supplementary Figure S29:** Phylogenetic reconstruction of the myriapod lysozyme C-like family by ML under Blosum62+I+G4 (chosen according to BIC) displayed as mid-point rooted tree. Sequences identified in venom proteomes are coloured red, while non-chilopod sequences are coloured blue. Bootstrap support values are shown at each node, and nodes with support < 50 are collapsed into multifurcations.

## Supplementary Figure S30

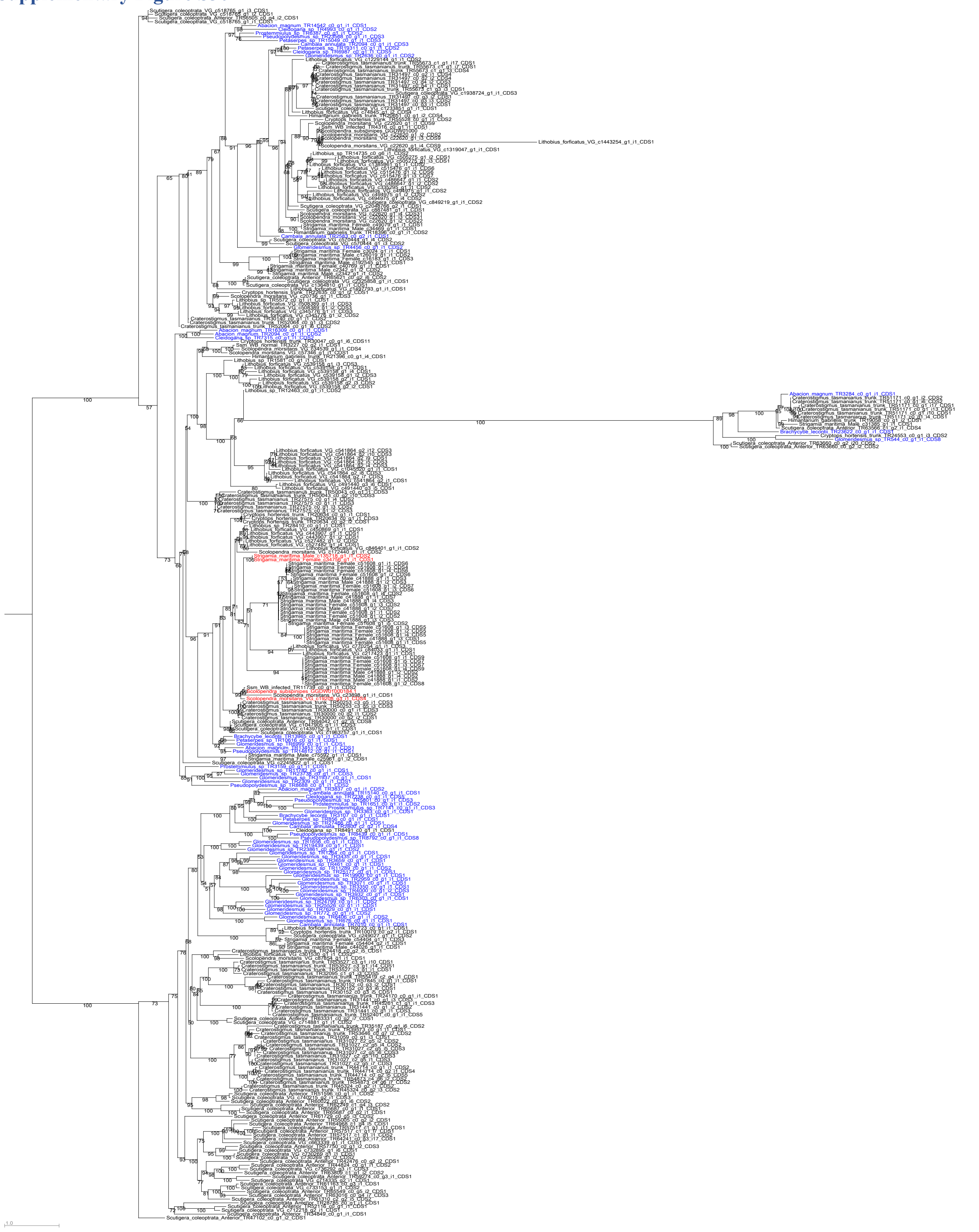

**Supplementary Figure S30:** Phylogenetic reconstruction of the myriapod PAM-like family by ML under WAG+F+R6 (chosen according to BIC) displayed as mid-point rooted tree. Sequences identified in venom proteomes are coloured red, while non-chilopod sequences are coloured blue. Bootstrap support values are shown at each node, and nodes with support < 50 are collapsed into multifurcations.

## Supplementary Figure S31

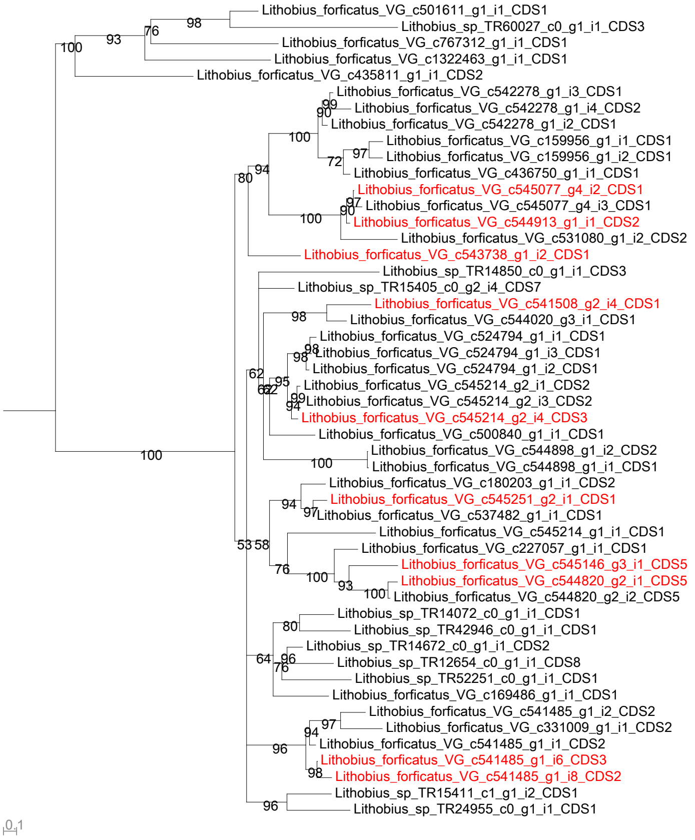


**Supplementary Figure S31:** Phylogenetic reconstruction of the myriapod PCPDP-like protein family by ML under WAG+F+I+G4 (chosen according to BIC) displayed as mid-point rooted tree. Sequences identified in venom proteomes are coloured red. Bootstrap support values are shown at each node, and nodes with support < 50 are collapsed into multifurcations.

## Supplementary Figure S32

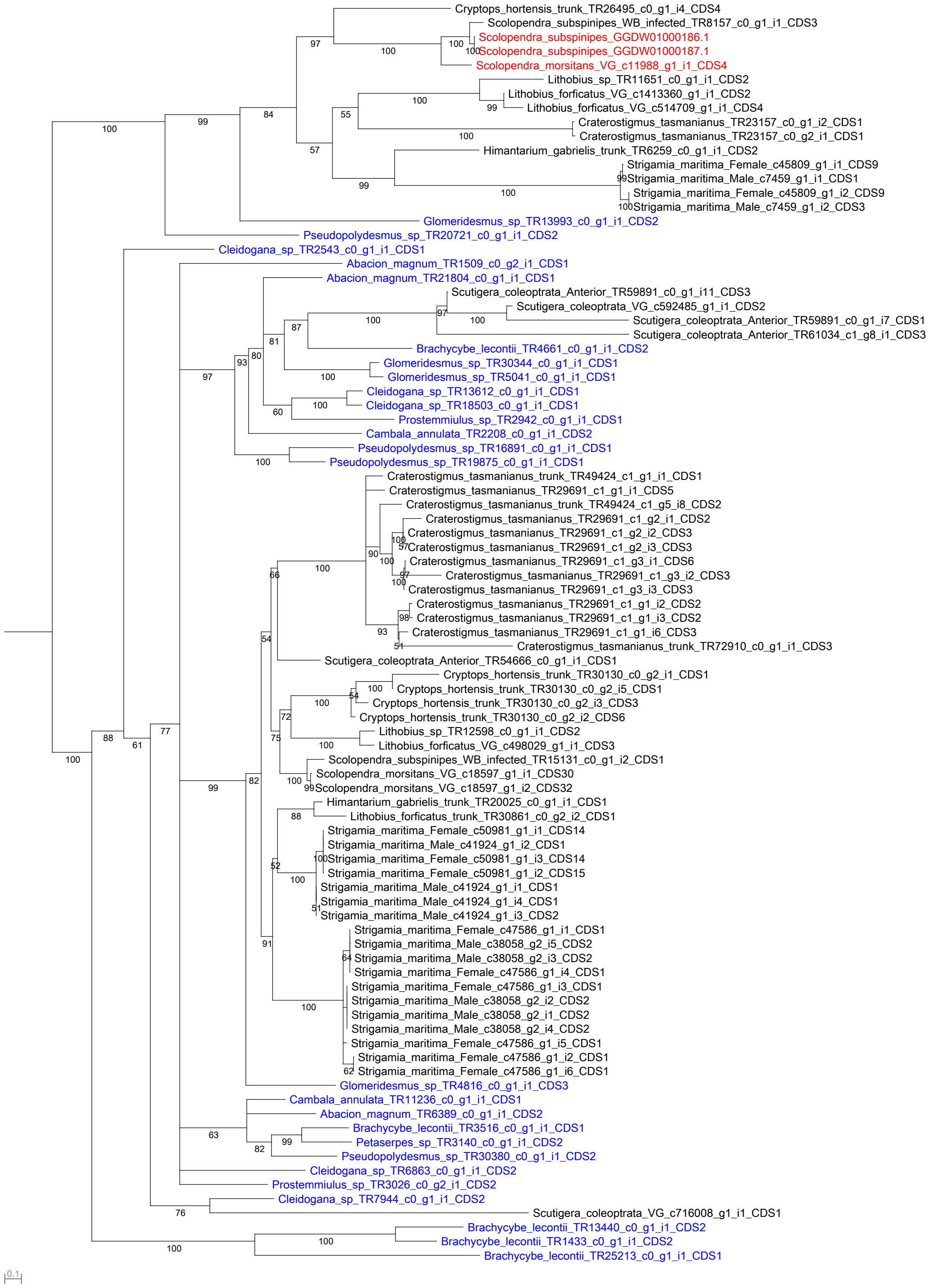


**Supplementary Figure S32:** Phylogenetic reconstruction of the myriapod PEBP-like protein family by ML under WAG+G4 (chosen according to BIC) displayed as mid-point rooted tree. Sequences identified in venom proteomes are coloured red, while non-chilopod sequences are coloured blue. Bootstrap support values are shown at each node, and nodes with support < 50 are collapsed into multifurcations.

## Supplementary Figure S33

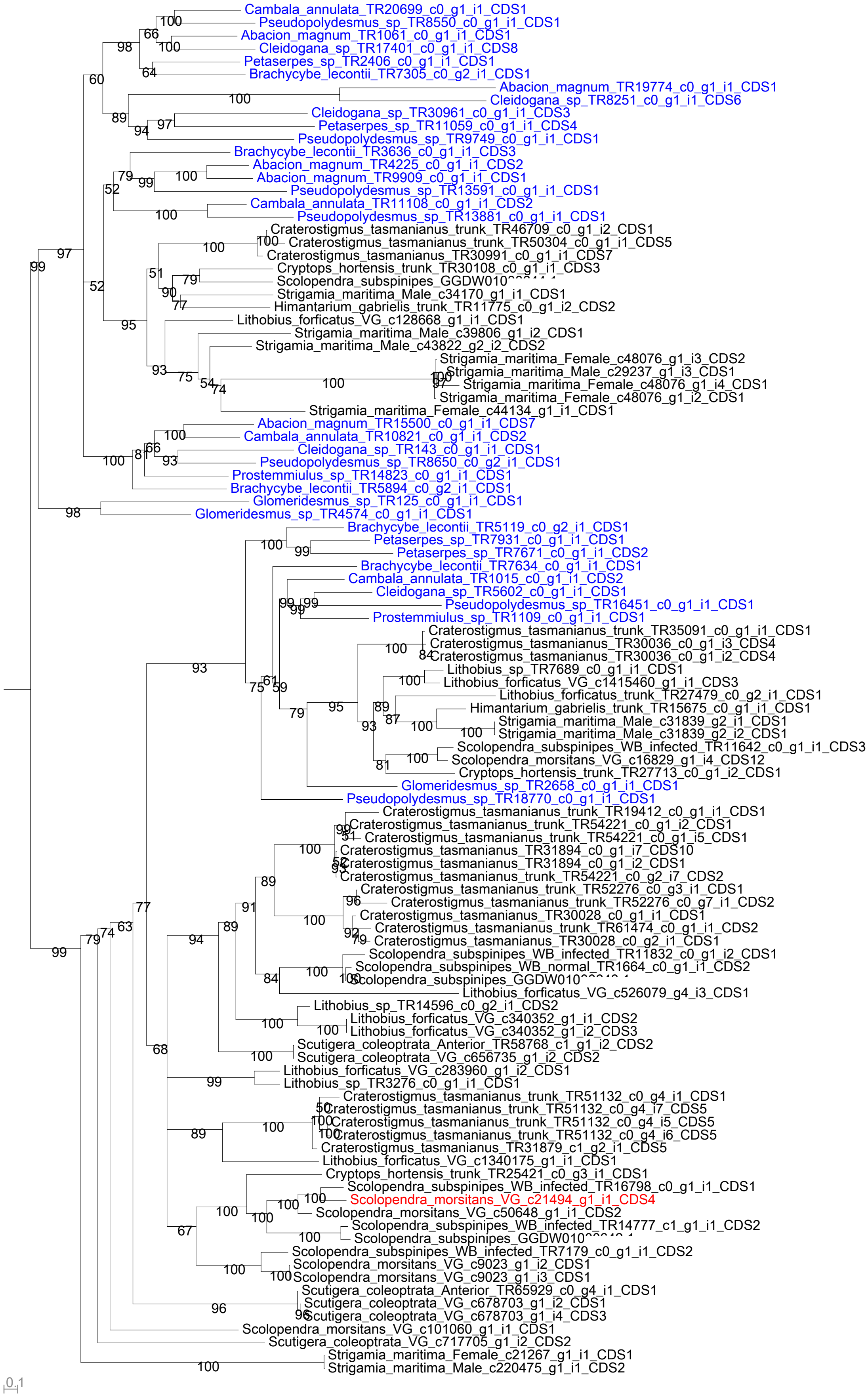

**Supplementary Figure S33:** Phylogenetic reconstruction of the myriapod phosphodiesterase 2 family by ML under LG+R5 (chosen according to BIC) displayed as mid-point rooted tree. Sequences identified in venom proteomes are coloured red, while non-chilopod sequences are coloured blue. Bootstrap support values are shown at each node, and nodes with support < 50 are collapsed into multifurcations.

# Supplementary Figure S34

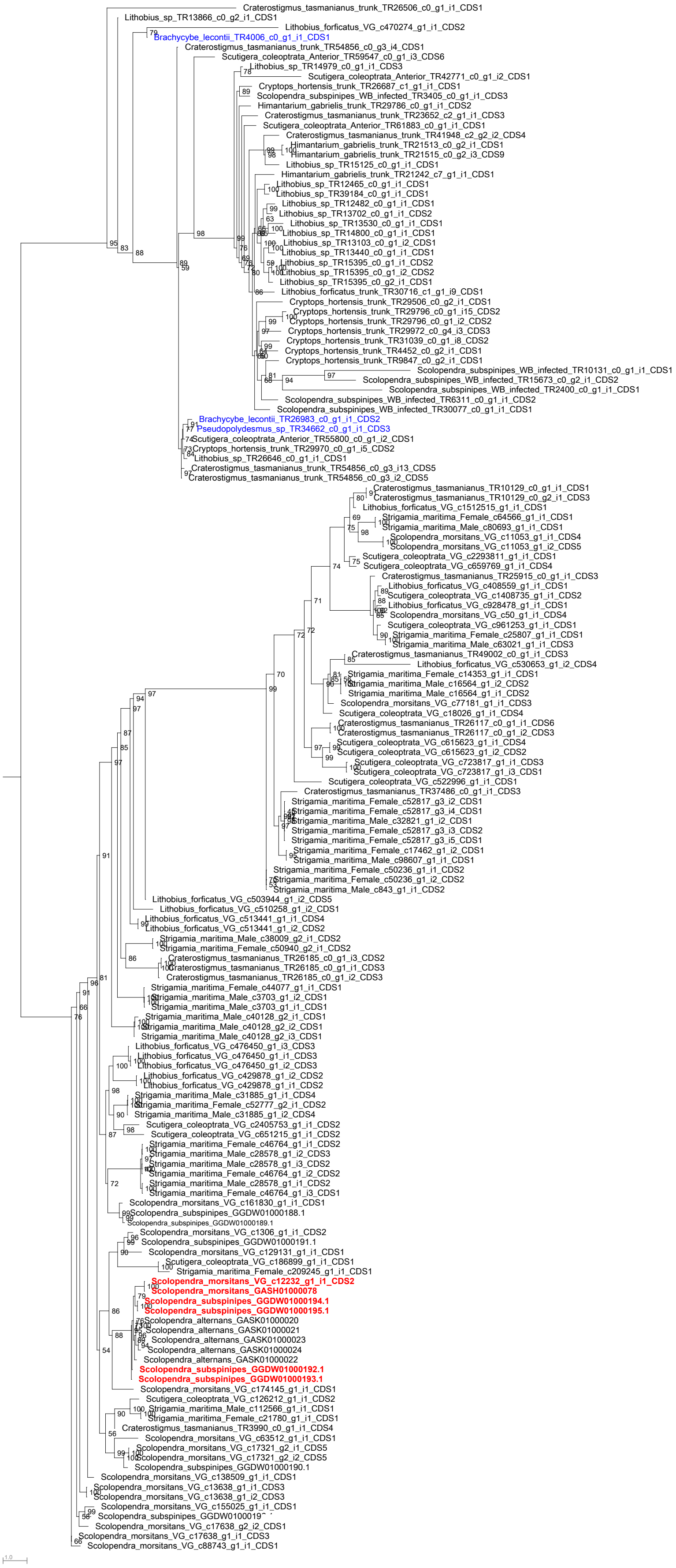


**Supplementary Figure S34:** Phylogenetic reconstruction of the myriapod $PLA_2$ family by ML under VT+R5 (chosen according to BIC) displayed as mid-point rooted tree. Sequences identified in venom proteomes are coloured red, while non-chilopod sequences are coloured blue. Bootstrap support values are shown at each node, and nodes with support < 50 are collapsed into multifurcations.

## Supplementary Figure S35

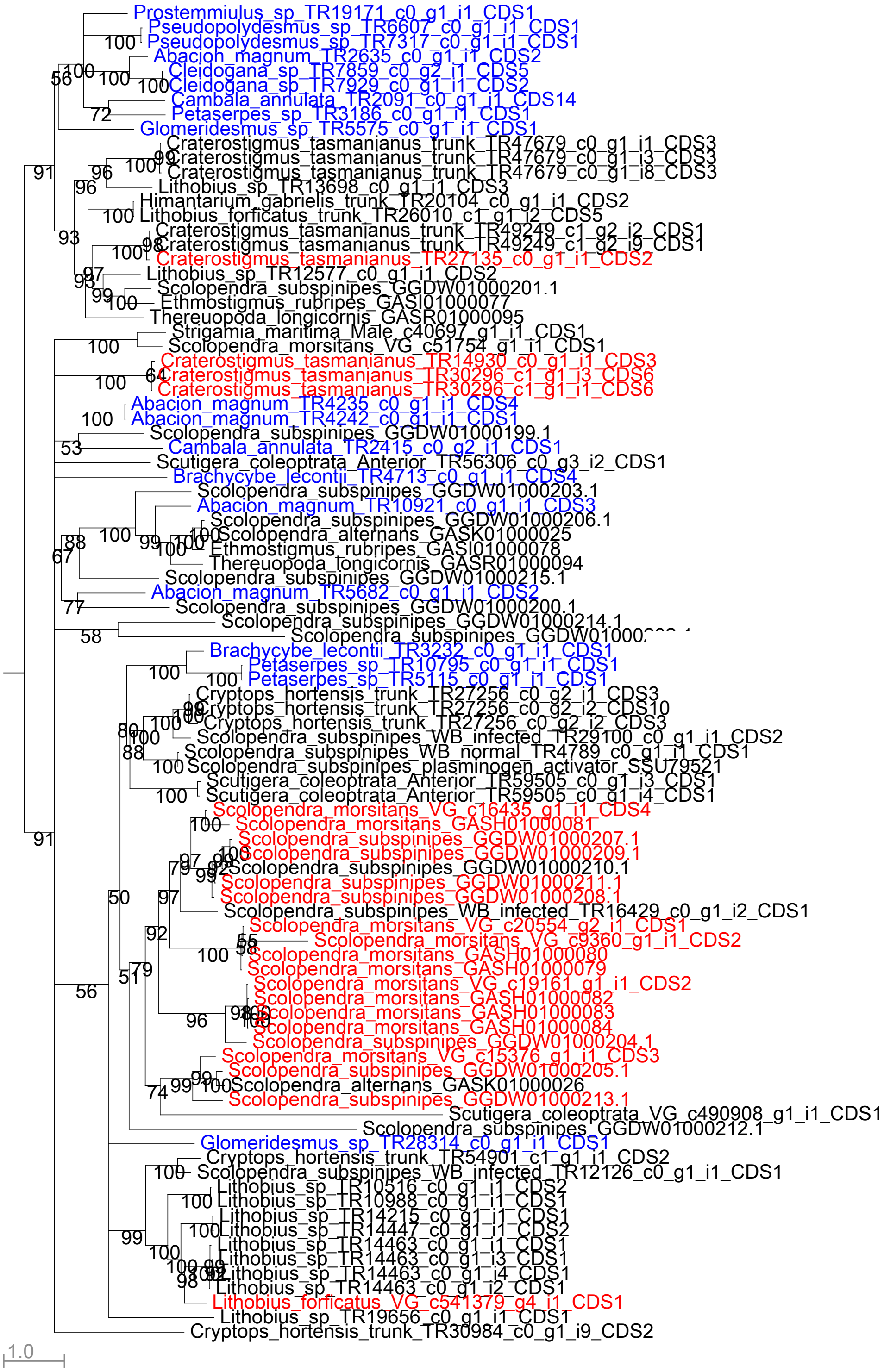

**Supplementary Figure S35:** Phylogenetic reconstruction of the myriapod S1 peptidase family by ML under VT+R5 (chosen according to BIC) displayed as mid-point rooted tree. Sequences identified in venom proteomes are coloured red, while non-chilopod sequences are coloured blue. Bootstrap support values are shown at each node, and nodes with support < 50 are collapsed into multifurcations.

## Supplementary Figure S36

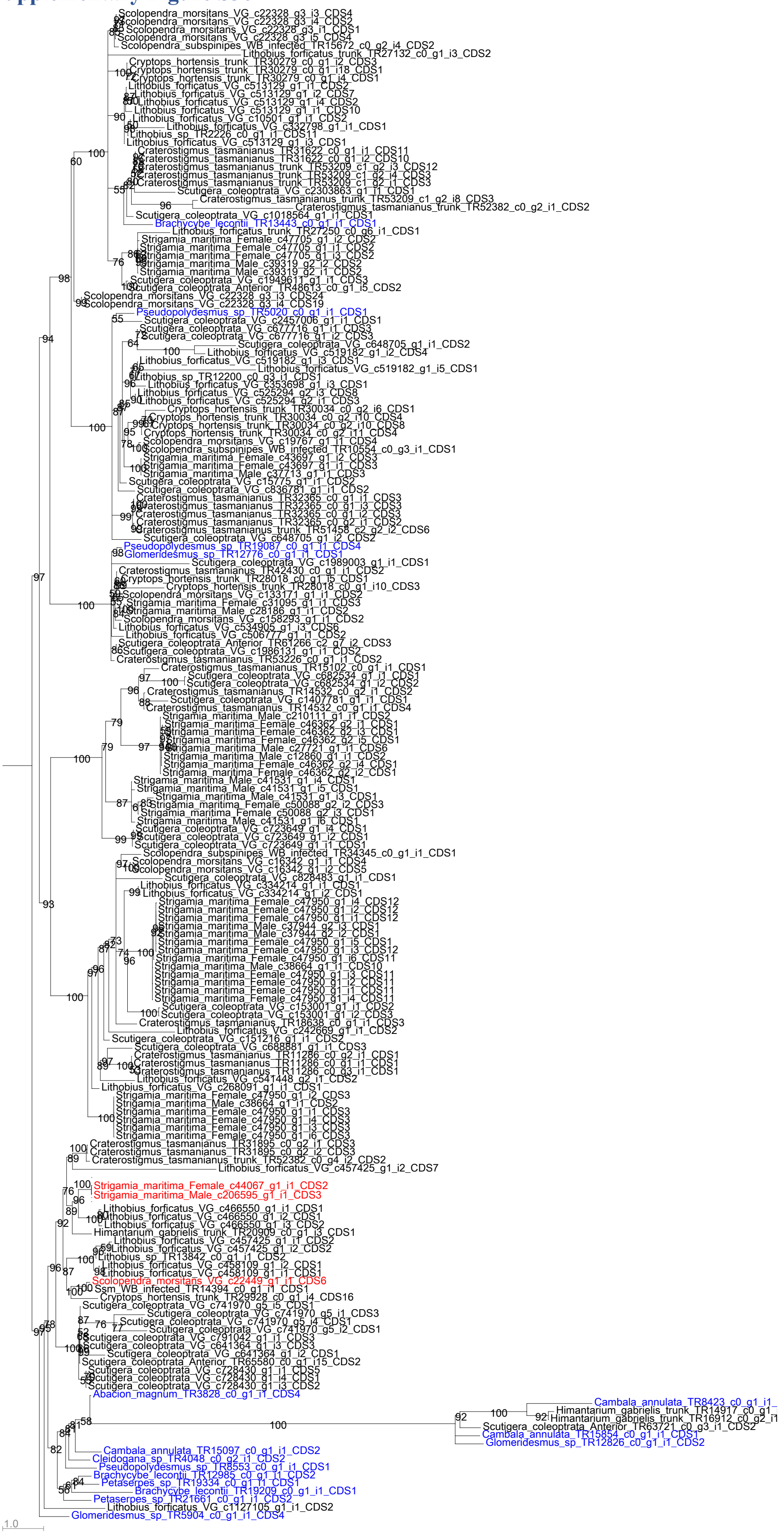

**Supplementary Figure S36:** Phylogenetic reconstruction of the myriapod S8 peptidase family by ML under PMB+R5 (chosen according to BIC) displayed as mid-point rooted tree. Sequences identified in venom proteomes are coloured red, while non-chilopod sequences are coloured blue. Bootstrap support values are shown at each node, and nodes with support < 50 are collapsed into multifurcations.

## Supplementary Figure S37

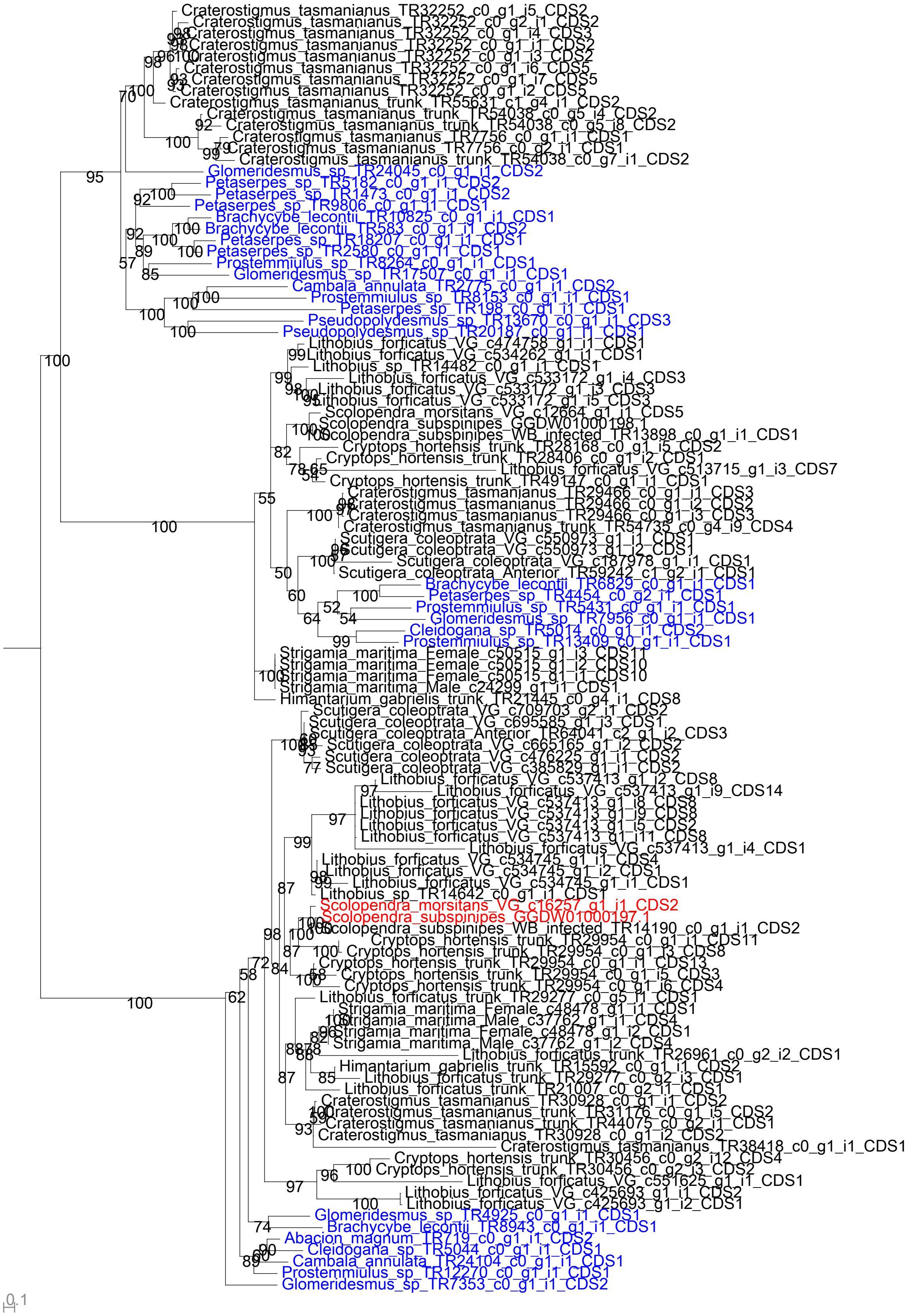

**Supplementary Figure S37:** Phylogenetic reconstruction of myriapod S10 peptidases by ML under WAG+F+G4 (chosen according to BIC) displayed as mid-point rooted tree. Sequences identified in venom proteomes are coloured red, while non-chilopod sequences are coloured blue. Bootstrap support values are shown at each node, and nodes with support < 50 are collapsed into multifurcations.

## Supplementary Figure S38

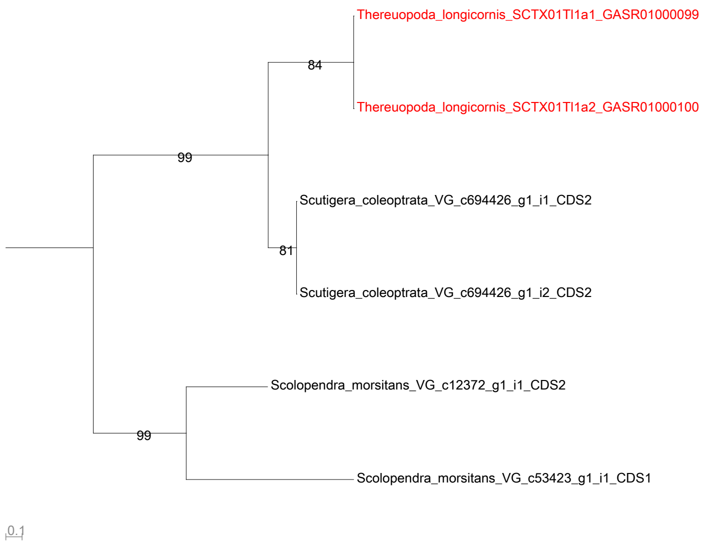


**Supplementary Figure S38:** Phylogenetic reconstruction of the myriapod SCTX01 peptide family by ML under JTT+I (chosen according to BIC) displayed as mid-point rooted tree. Sequences identified in venom proteomes are coloured red. Bootstrap support values are shown at each node, and nodes with support < 50 are collapsed into multifurcations.

## Supplementary Figure S39

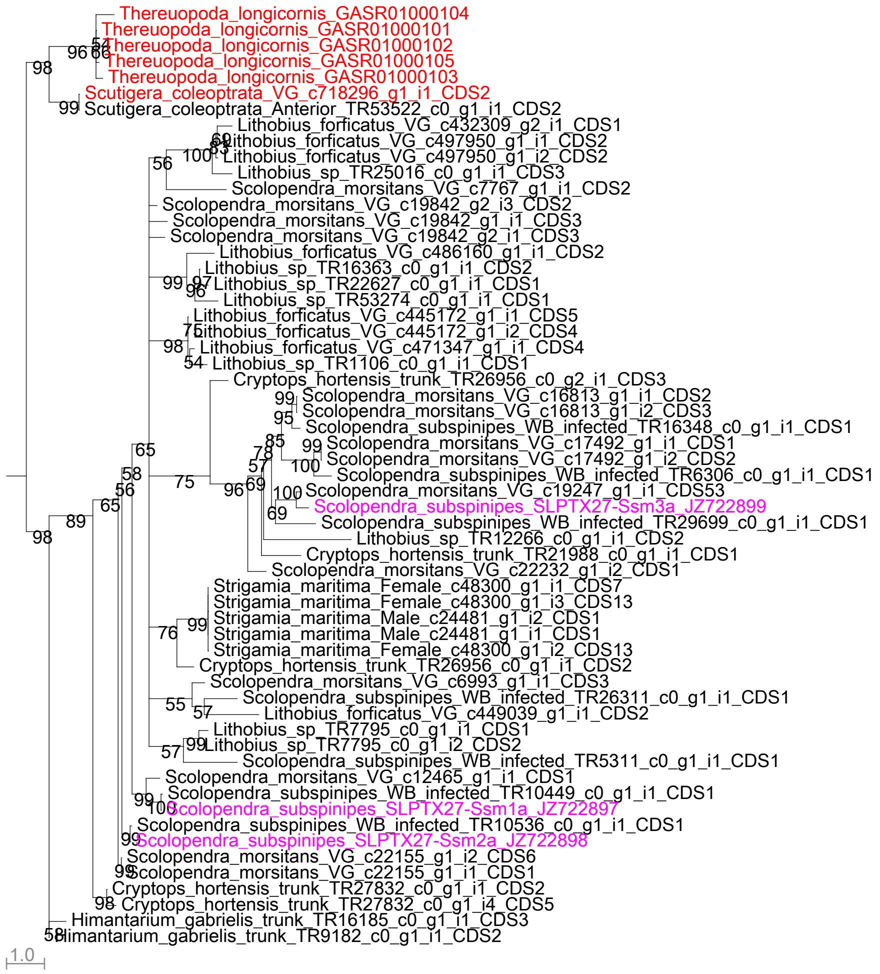


**Supplementary Figure S39:** Phylogenetic reconstruction of the SCTX02 peptide family by ML under VT+R4 (chosen according to BIC) displayed as mid-point rooted tree. Sequences identified in venom proteomes are coloured red, while sequences previously reported by Rong et al. (2015) but which lack proteomic evidence are coloured purple. Bootstrap support values are shown at each node, and nodes with support < 50 are collapsed into multifurcations.

## Supplementary Figure S40

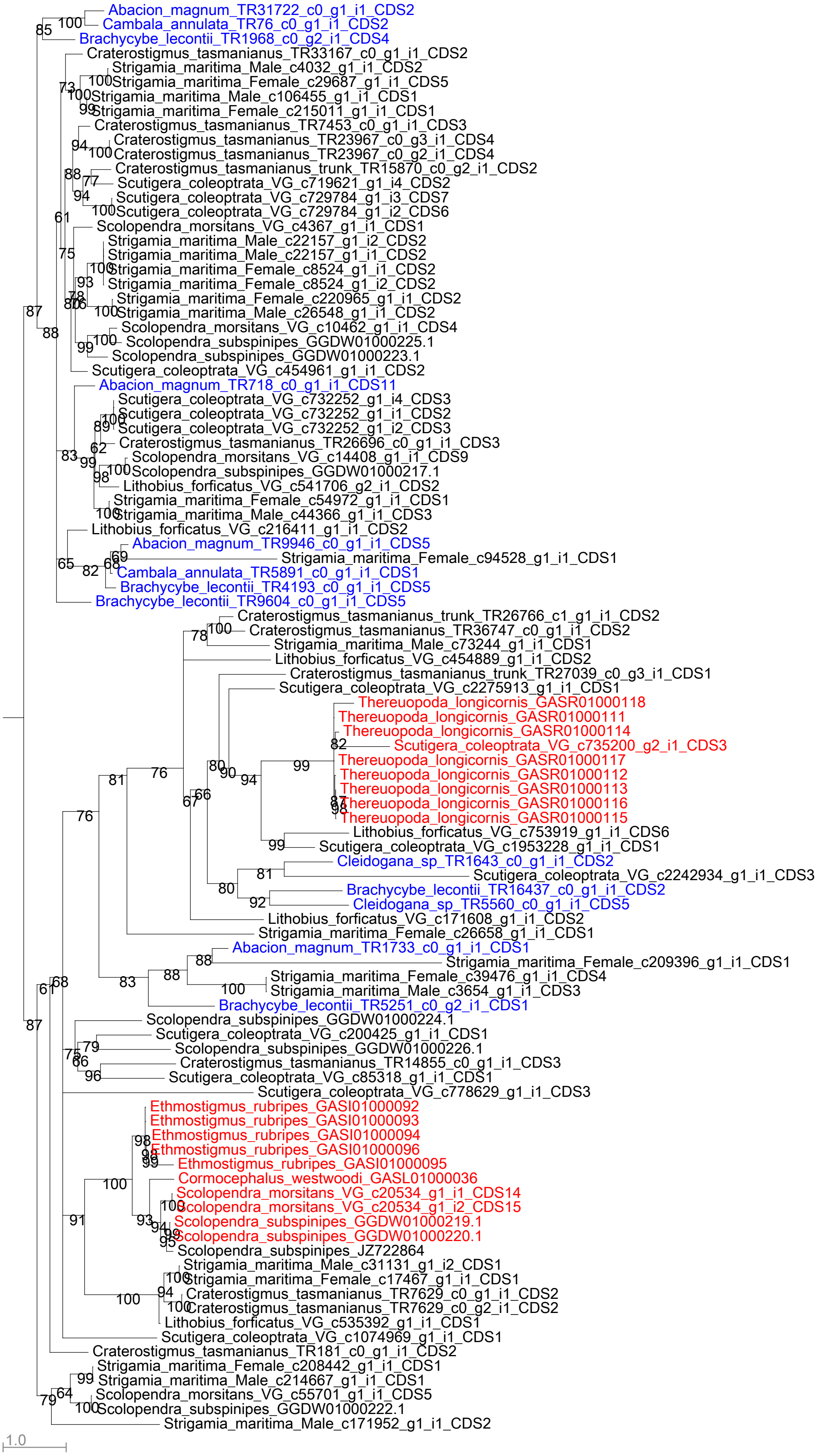


**Supplementary Figure S40:** Phylogenetic reconstruction of the myriapod SLPTX01-like family by ML under VT+R5 (chosen according to BIC) displayed as mid-point rooted tree. Sequences identified in venom proteomes are coloured red, while non-chilopod sequences are coloured blue. Bootstrap support values are shown at each node, and nodes with support < 50 are collapsed into multifurcations.

## Supplementary Figure S41

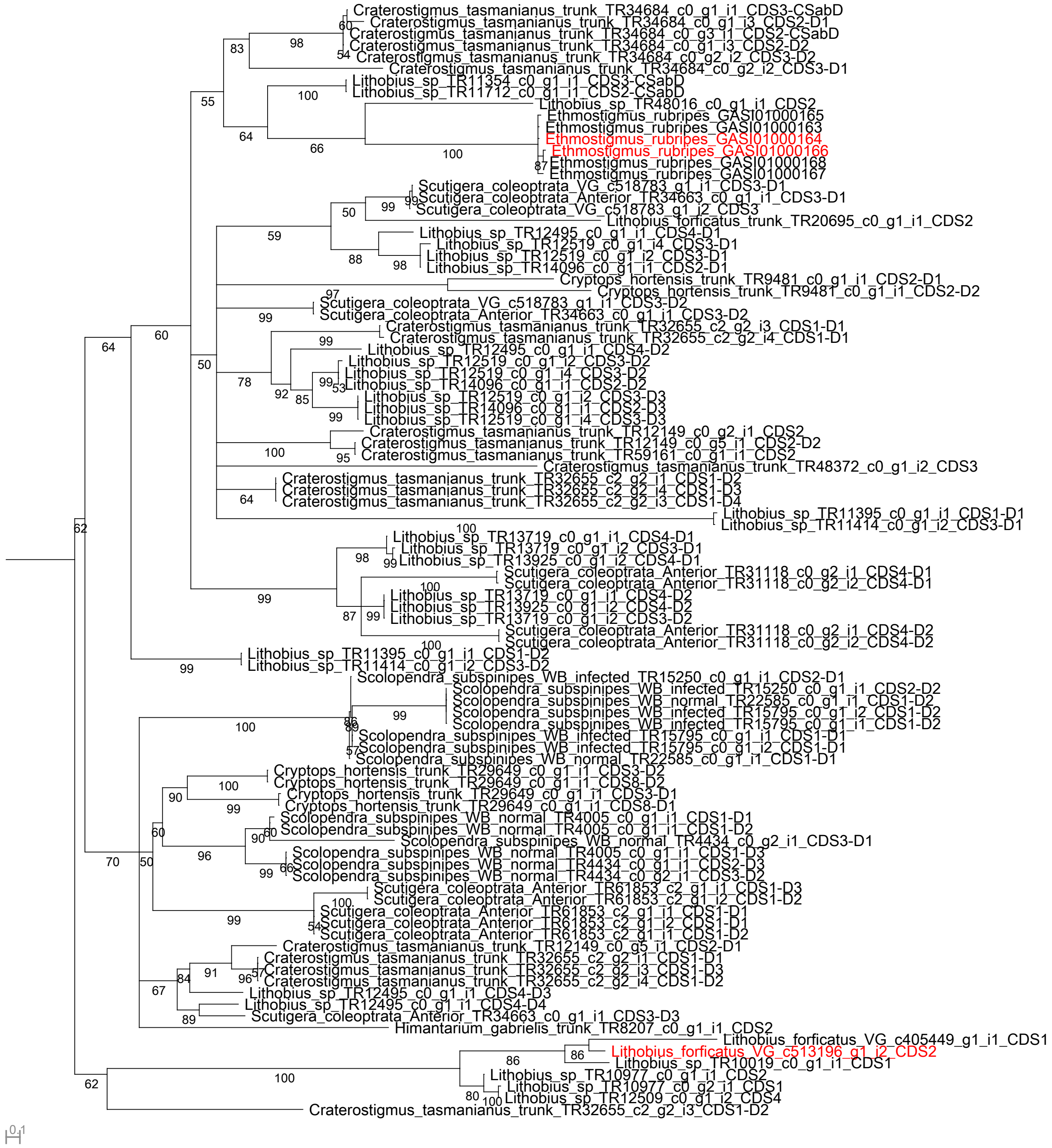

**Supplementary Figure S41:** Phylogenetic reconstruction of the SLPTX02 peptide family by ML under WAG+I+G4 (chosen according to BIC) displayed as mid-point rooted tree. Sequences identified in venom proteomes are coloured red. Bootstrap support values are shown at each node, and nodes with support < 50 are collapsed into multifurcations.

## Supplementary Figure S42

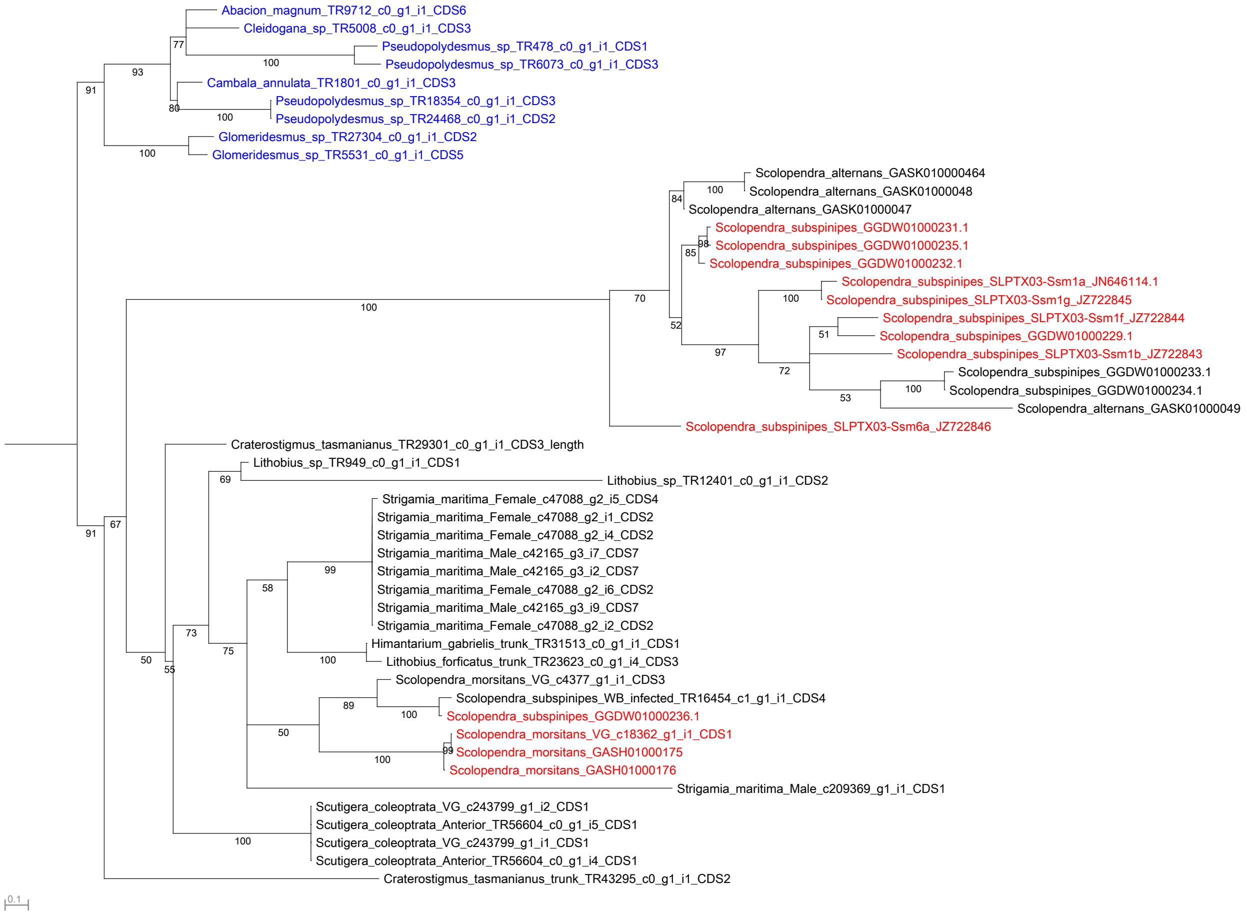


**Supplementary Figure S42:** Phylogenetic reconstruction of the myriapod CHH/ITP/SLPTX03 peptide family by ML under VT+I+G4 (chosen according to BIC) displayed as mid-point rooted tree. Sequences identified in venom proteomes are coloured red, while non-chilopod sequences are coloured blue. Bootstrap support values are shown at each node, and nodes with support < 50 are collapsed into multifurcations.

## Supplementary Figure S43

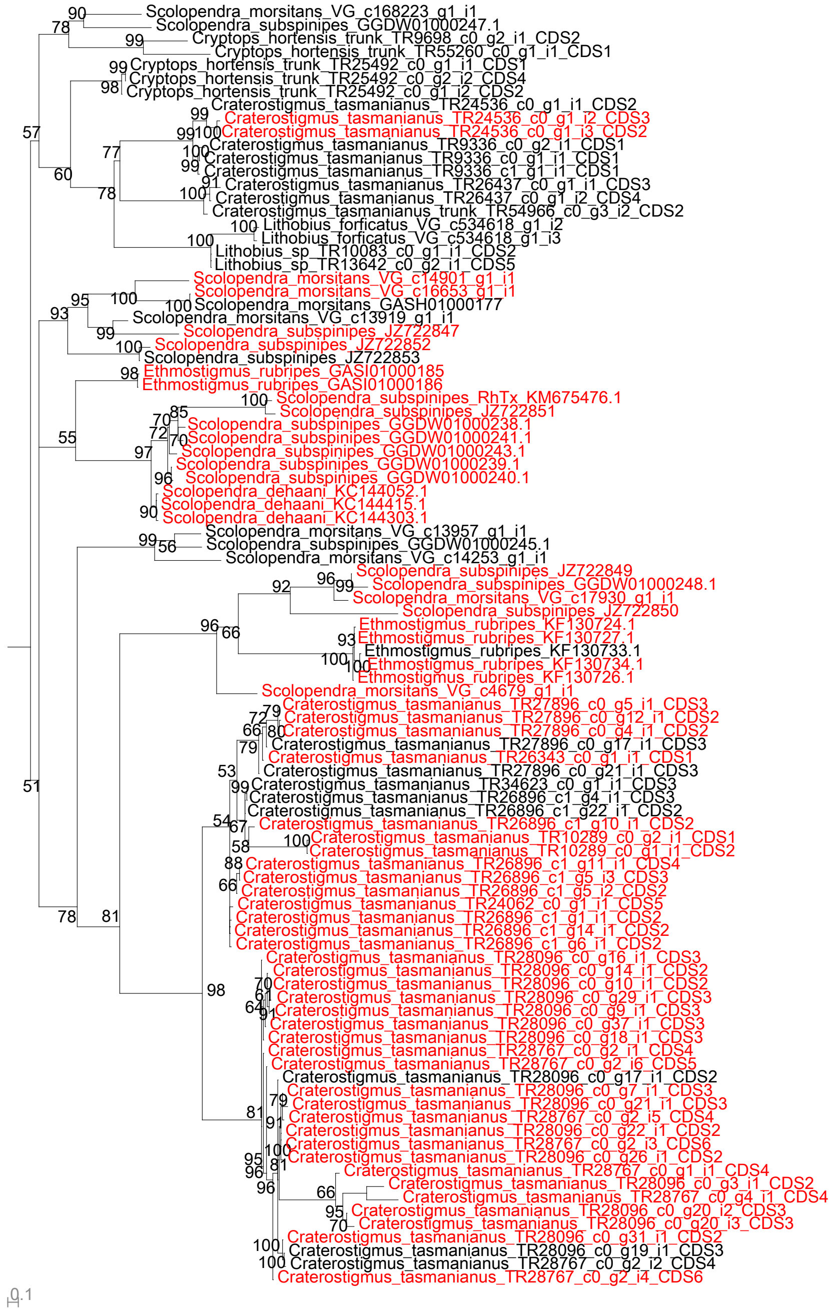

**Supplementary Figure S43:** Phylogenetic reconstruction of the SLPTX04 peptide family by ML under VT+I+G4 (chosen according to BIC) displayed as mid-point rooted tree. Sequences identified in venom proteomes are coloured red. Bootstrap support values are shown at each node, and nodes with support < 50 are collapsed into multifurcations.

## Supplementary Figure S44

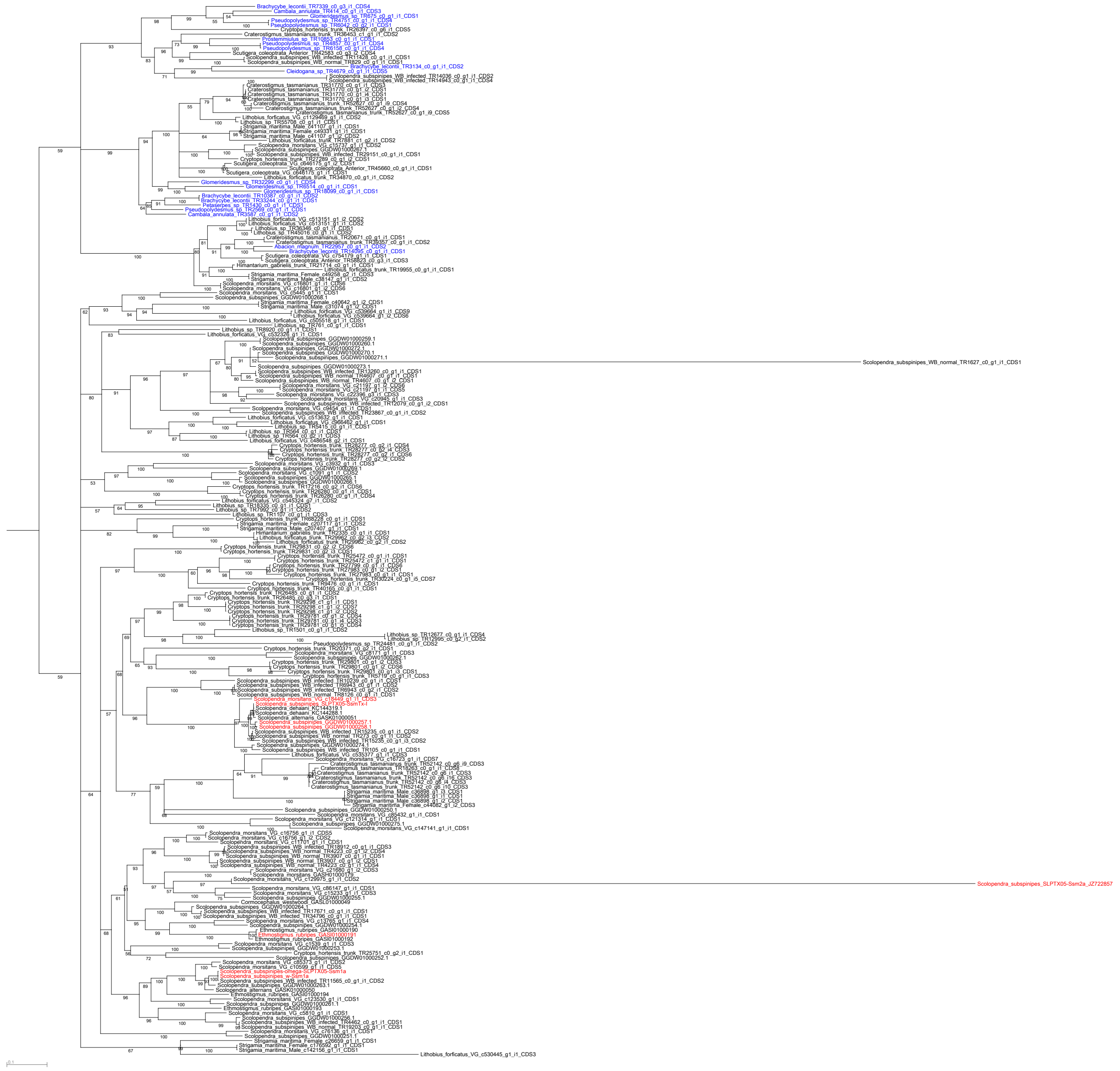

**Supplementary Figure S44:** Phylogenetic reconstruction of the SLPTX05 peptide family by ML under VT+R6 (chosen according to BIC) displayed as mid-point rooted tree. Sequences identified in venom proteomes are coloured red, while non-chilopod sequences are coloured blue. Bootstrap support values are shown at each node, and nodes with support < 50 are collapsed into multifurcations.

## Supplementary Figure S45

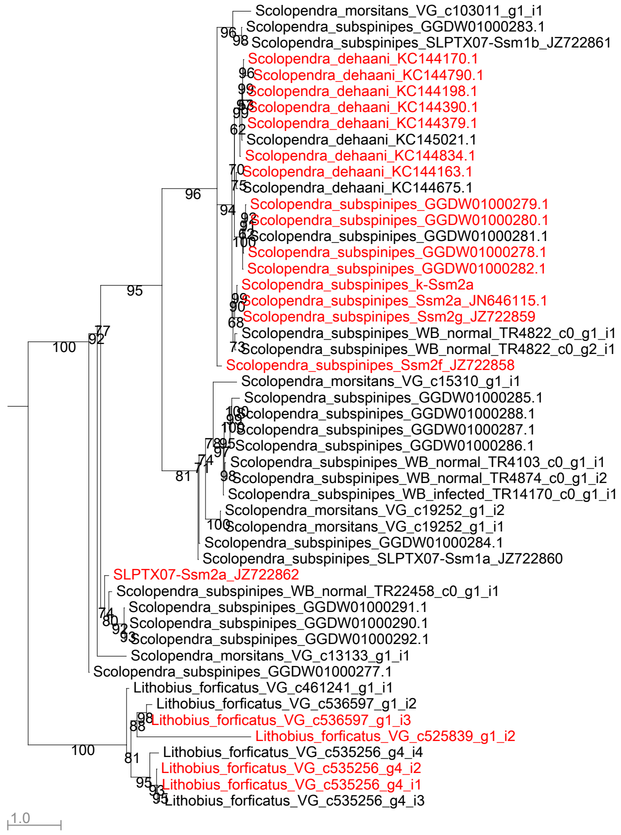


**Supplementary Figure S45:** Phylogenetic reconstruction of the SLPTX07 peptide family by ML under JTT+I+G4 (chosen according to BIC) displayed as mid-point rooted tree. Sequences identified in venom proteomes are coloured red. Bootstrap support values are shown at each node, and nodes with support < 50 are collapsed into multifurcations.

## Supplementary Figure S46

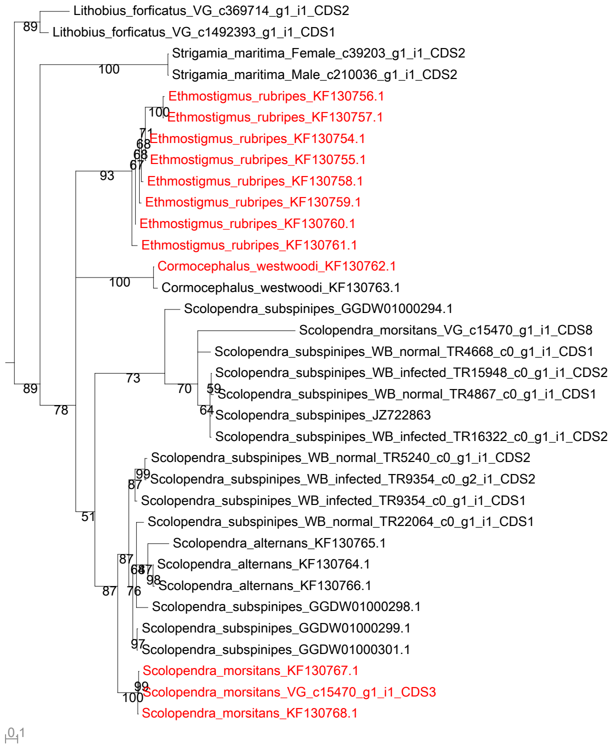


**Supplementary Figure S46:** Phylogenetic reconstruction of the SLPTX08 peptide family by ML under JTT+G4 (chosen according to BIC) displayed as mid-point rooted tree. Sequences identified in venom proteomes are coloured red. Bootstrap support values are shown at each node, and nodes with support < 50 are collapsed into multifurcations.

## Supplementary Figure S47

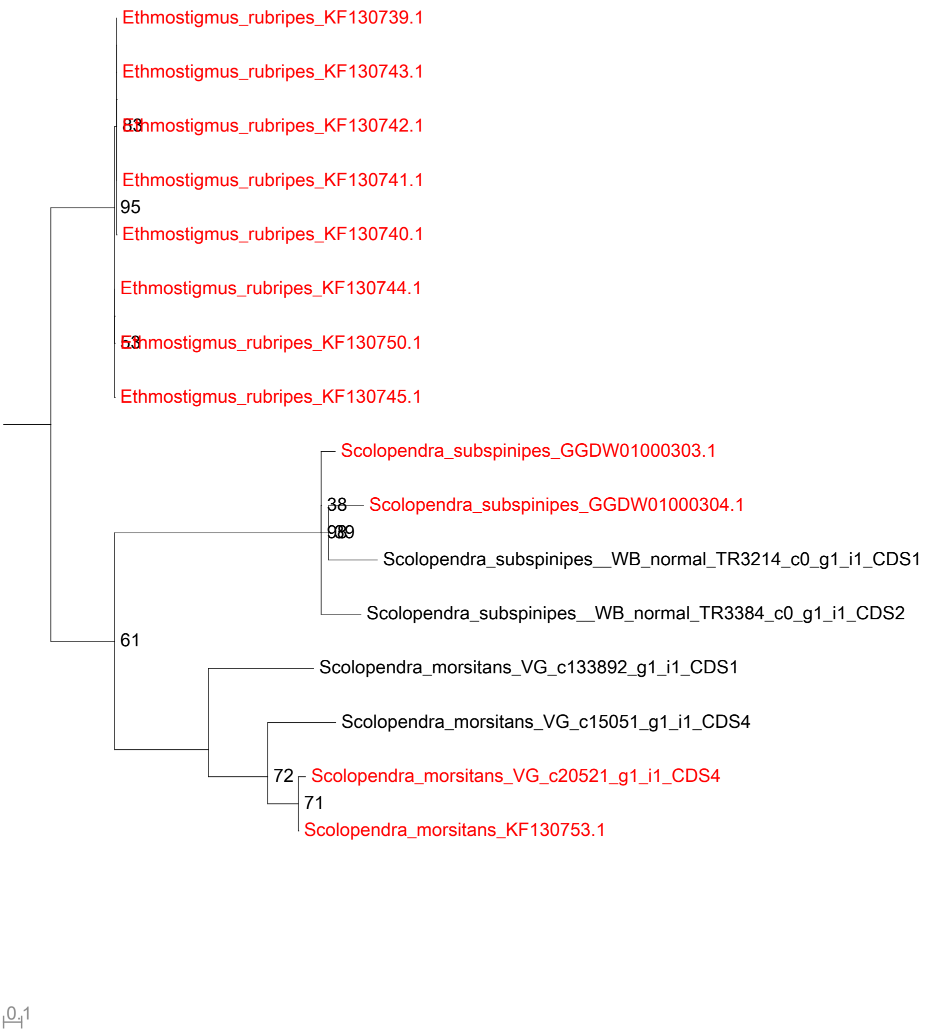


**Supplementary Figure S47:** Phylogenetic reconstruction of SLPTX09 peptide family by ML under DCMut+G4 (chosen according to BIC) displayed as mid-point rooted tree. Sequences identified in venom proteomes are coloured red. Bootstrap support values are shown at each node, and nodes with support < 50 are collapsed into multifurcations.

## Supplementary Figure S48

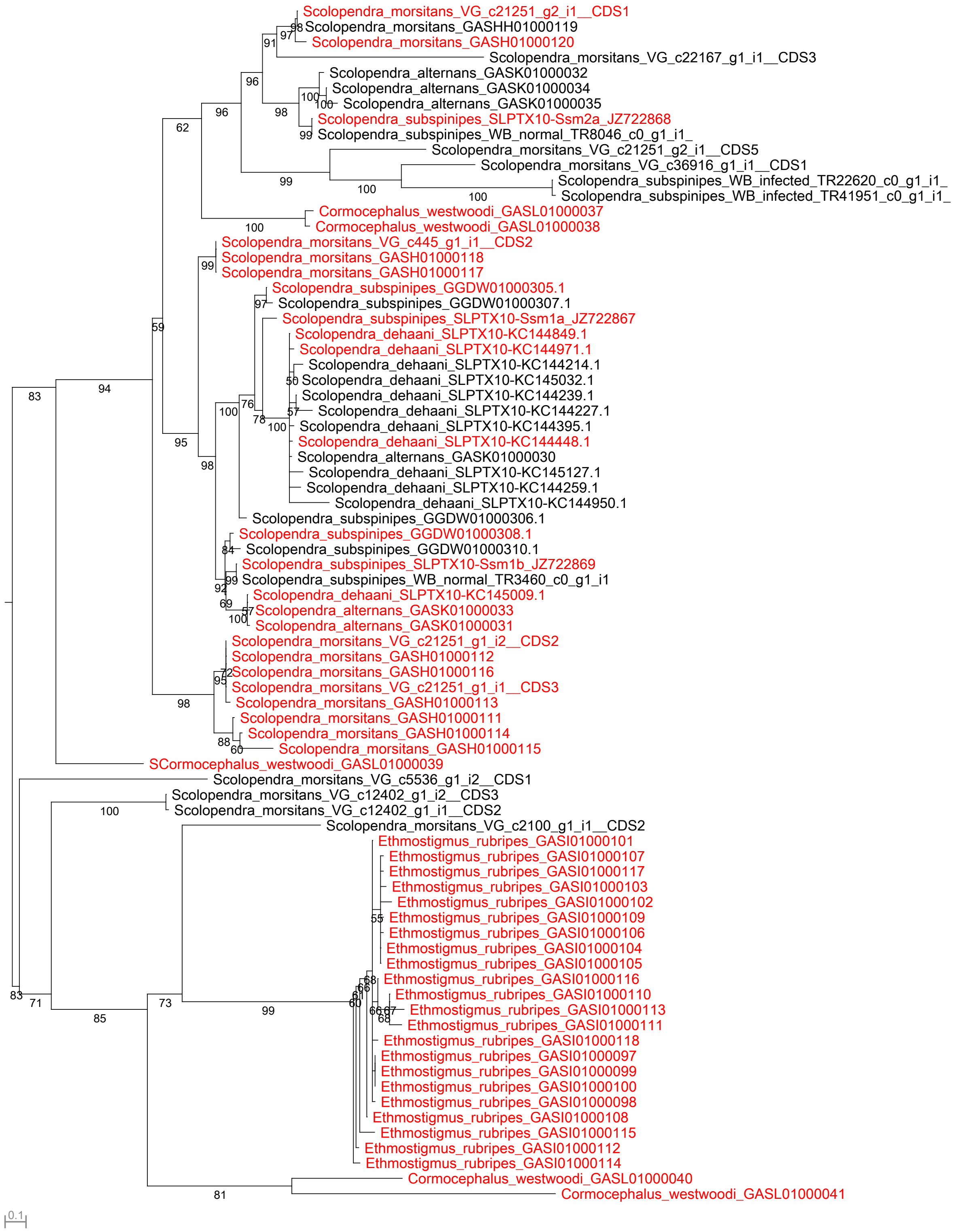


**Supplementary Figure S48:** Phylogenetic reconstruction of the SLPTX10 peptide family by ML under VT+I+G4 (chosen according to BIC) displayed as mid-point rooted tree. Sequences identified in venom proteomes are coloured red. Bootstrap support values are shown at each node, and nodes with support < 50 are collapsed into multifurcations.

## Supplementary Figure S49

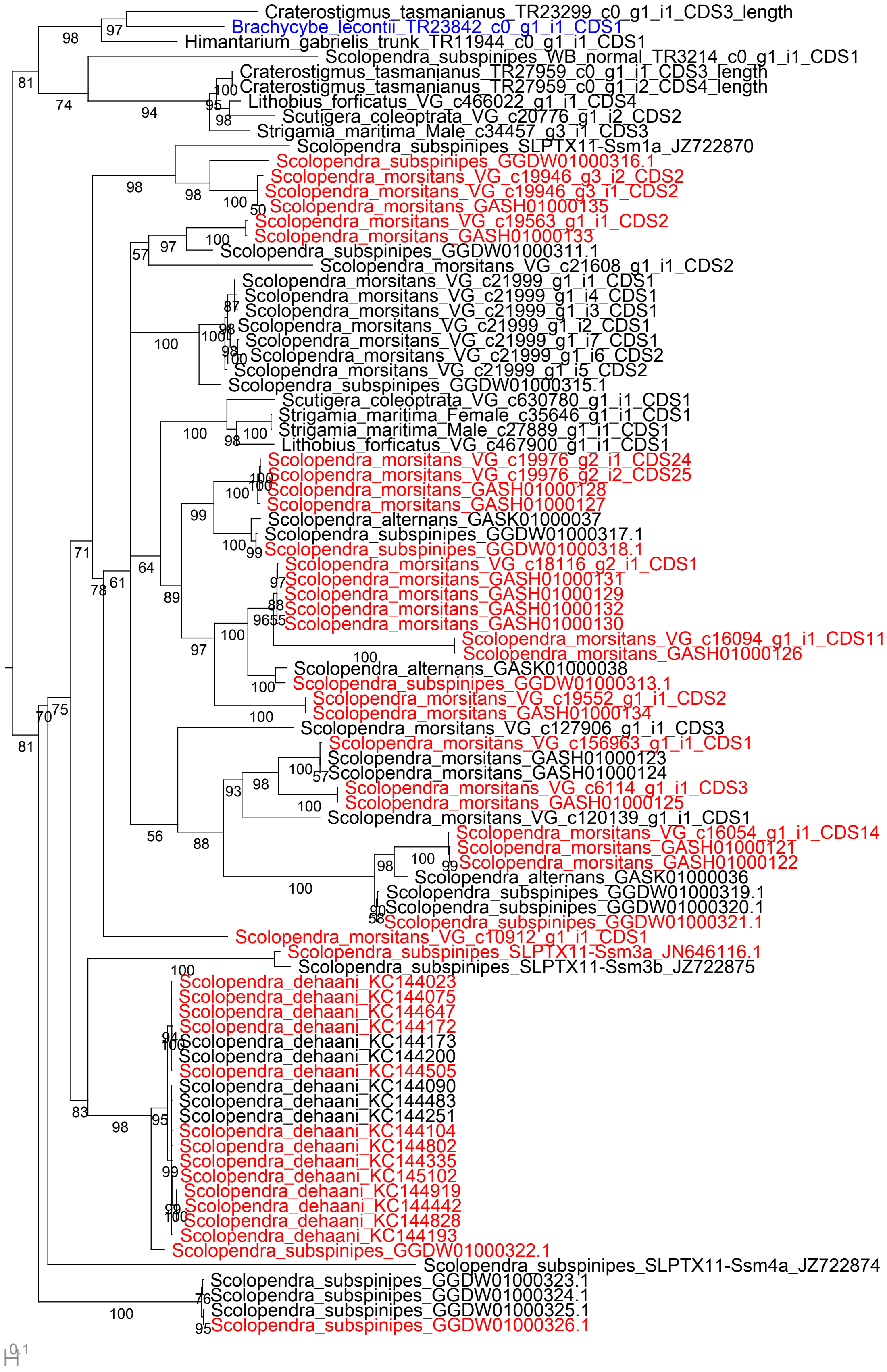


**Supplementary Figure S49:** Phylogenetic reconstruction of the SLPTX11 peptide family by ML under VT+R4 (chosen according to BIC) displayed as mid-point rooted tree. Sequences identified in venom proteomes are coloured red, while non-chilopod sequences are coloured blue. Bootstrap support values are shown at each node, and nodes with support < 50 are collapsed into multifurcations.

## Supplementary Figure S50

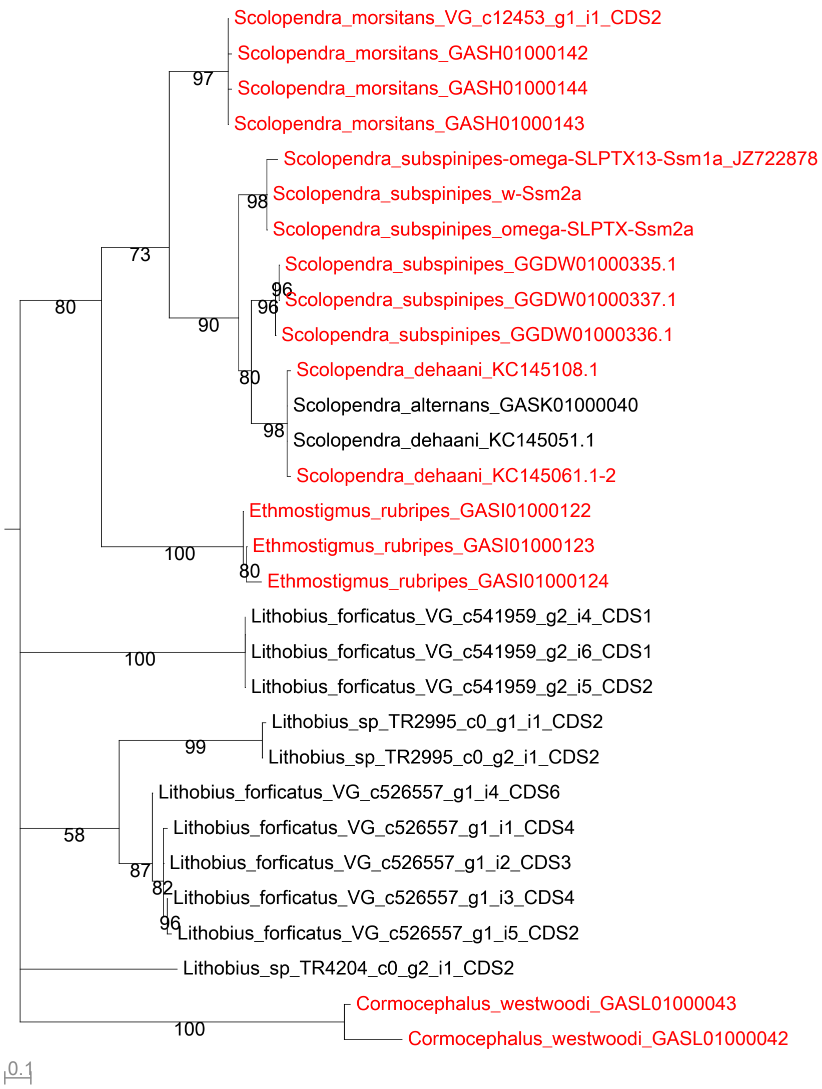


**Supplementary Figure S50:** Phylogenetic reconstruction of the SLPTX13 peptide family by ML under WAG+I+G4 (chosen according to BIC) displayed as mid-point rooted tree. Sequences identified in venom proteomes are coloured red. Bootstrap support values are shown at each node, and nodes with support < 50 are collapsed into multifurcations.

## Supplementary Figure S51

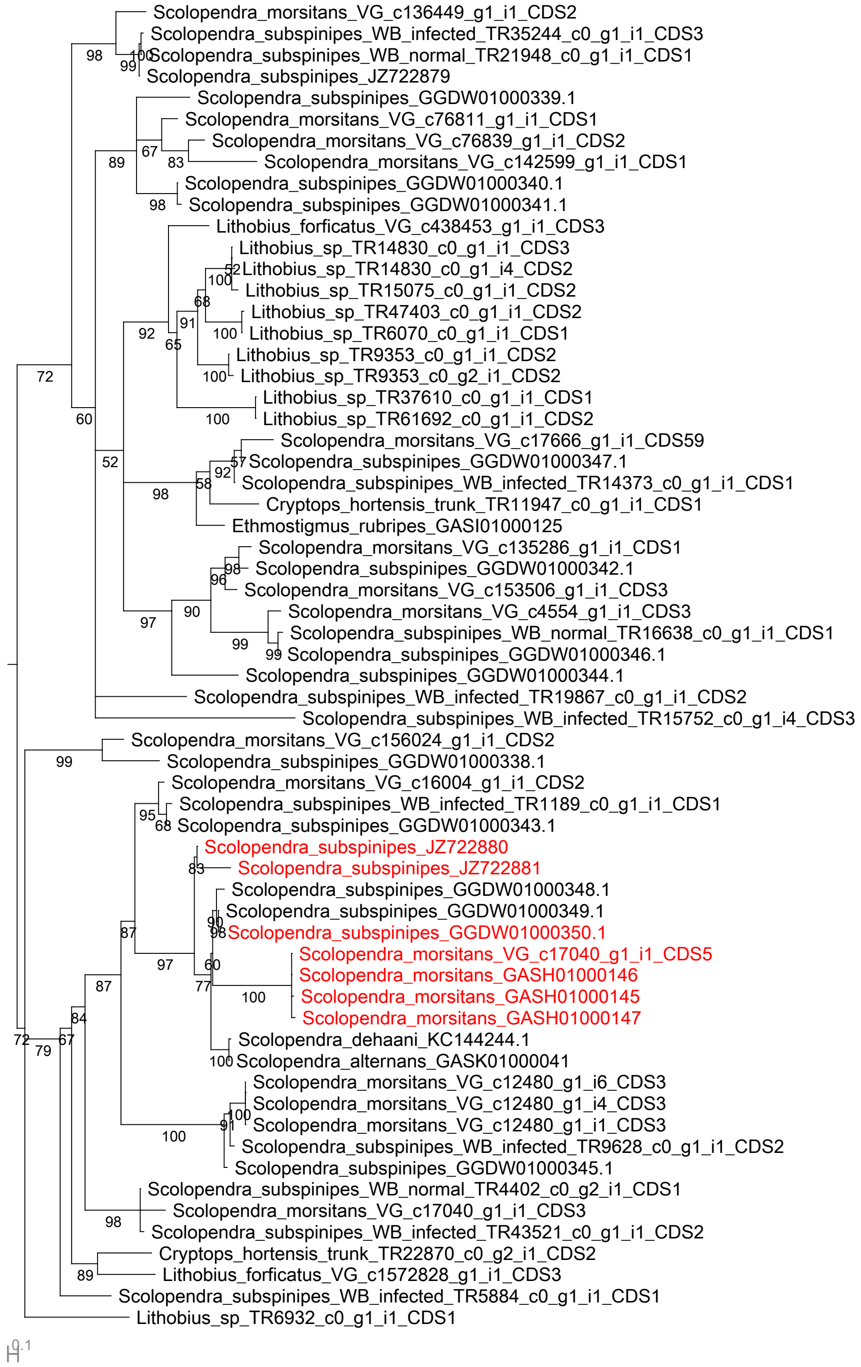


**Supplementary Figure S51:** Phylogenetic reconstruction of the SLPTX14 peptide family by ML under WAG+F+R6 (chosen according to BIC) displayed as mid-point rooted tree. Sequences identified in venom proteomes are coloured red. Bootstrap support values are shown at each node, and nodes with support < 50 are collapsed into multifurcations.

## Supplementary Figure S52

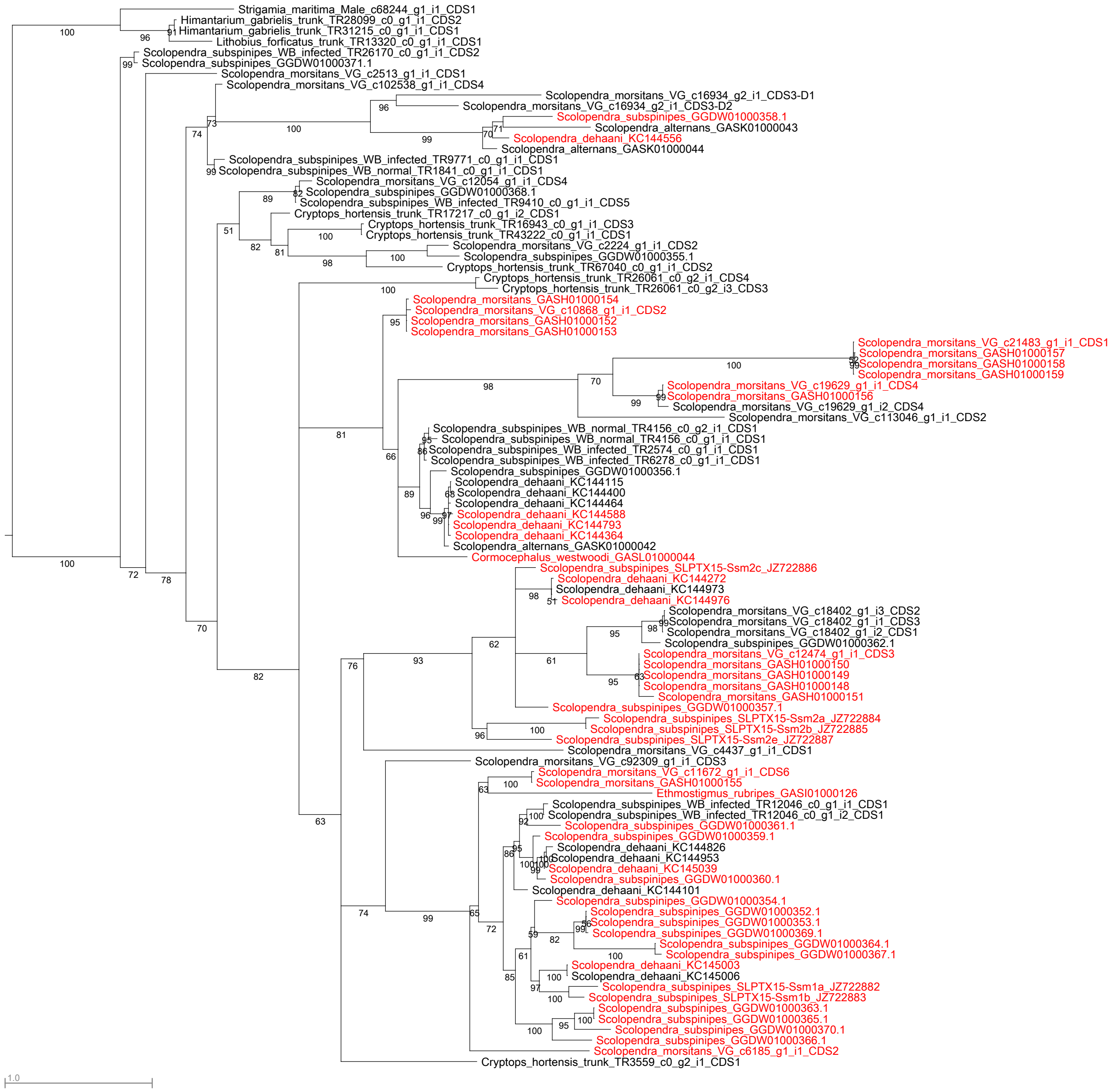

**Supplementary Figure S52:** Phylogenetic reconstruction of the SLPTX15 peptide family by ML under VT+R4 (chosen according to BIC) displayed as mid-point rooted tree. Sequences identified in venom proteomes are coloured red. Bootstrap support values are shown at each node, and nodes with support < 50 are collapsed into multifurcations.

## Supplementary Figure S53

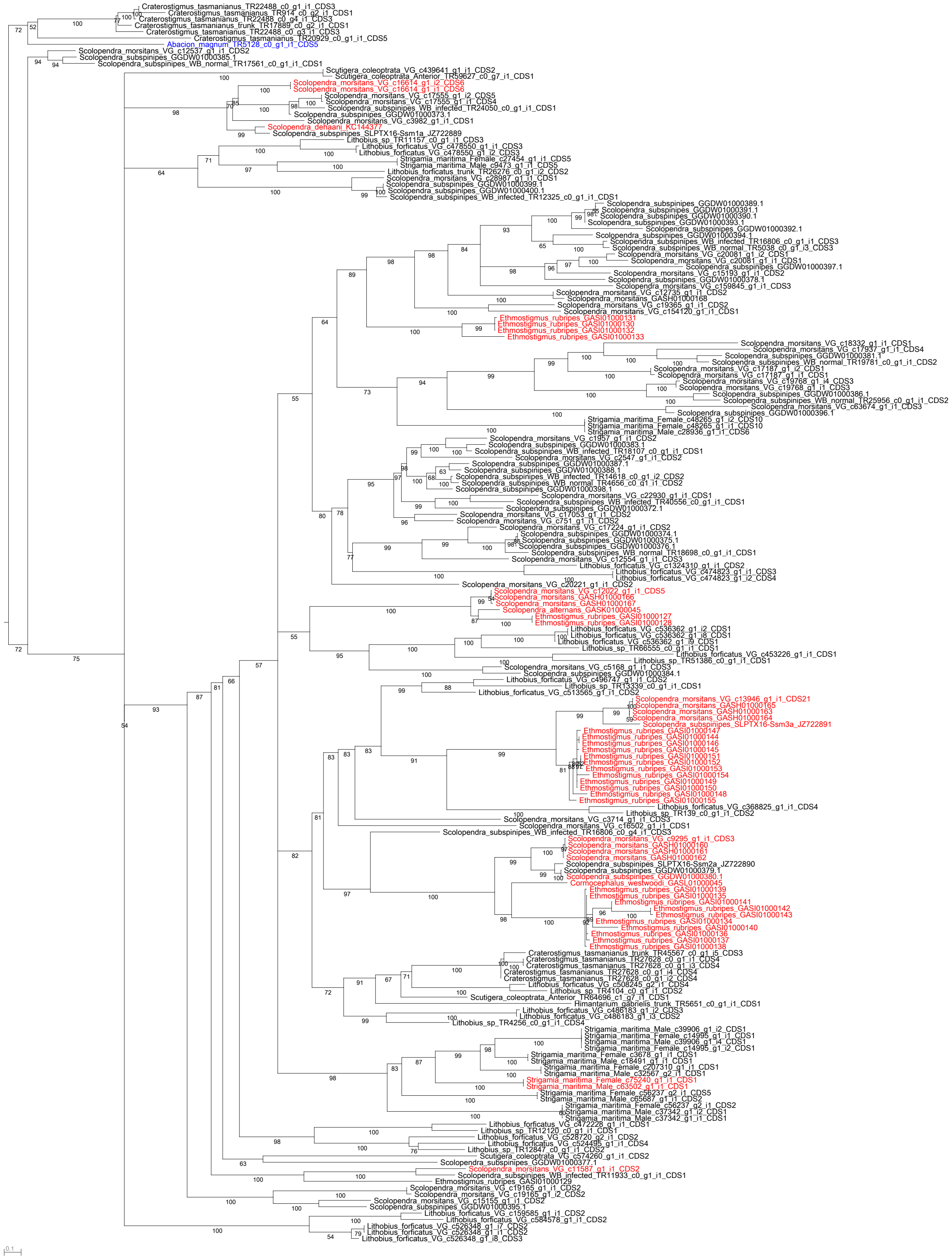

**Supplementary Figure S53:** Phylogenetic reconstruction of the SLPTX16 peptide family by ML under VT+R4 (chosen according to BIC) displayed as mid-point rooted tree. Sequences identified in venom proteomes are coloured red, while non-chilopod sequences are coloured blue. Bootstrap support values are shown at each node, and nodes with support < 50 are collapsed into multifurcations.

## Supplementary Figure S54

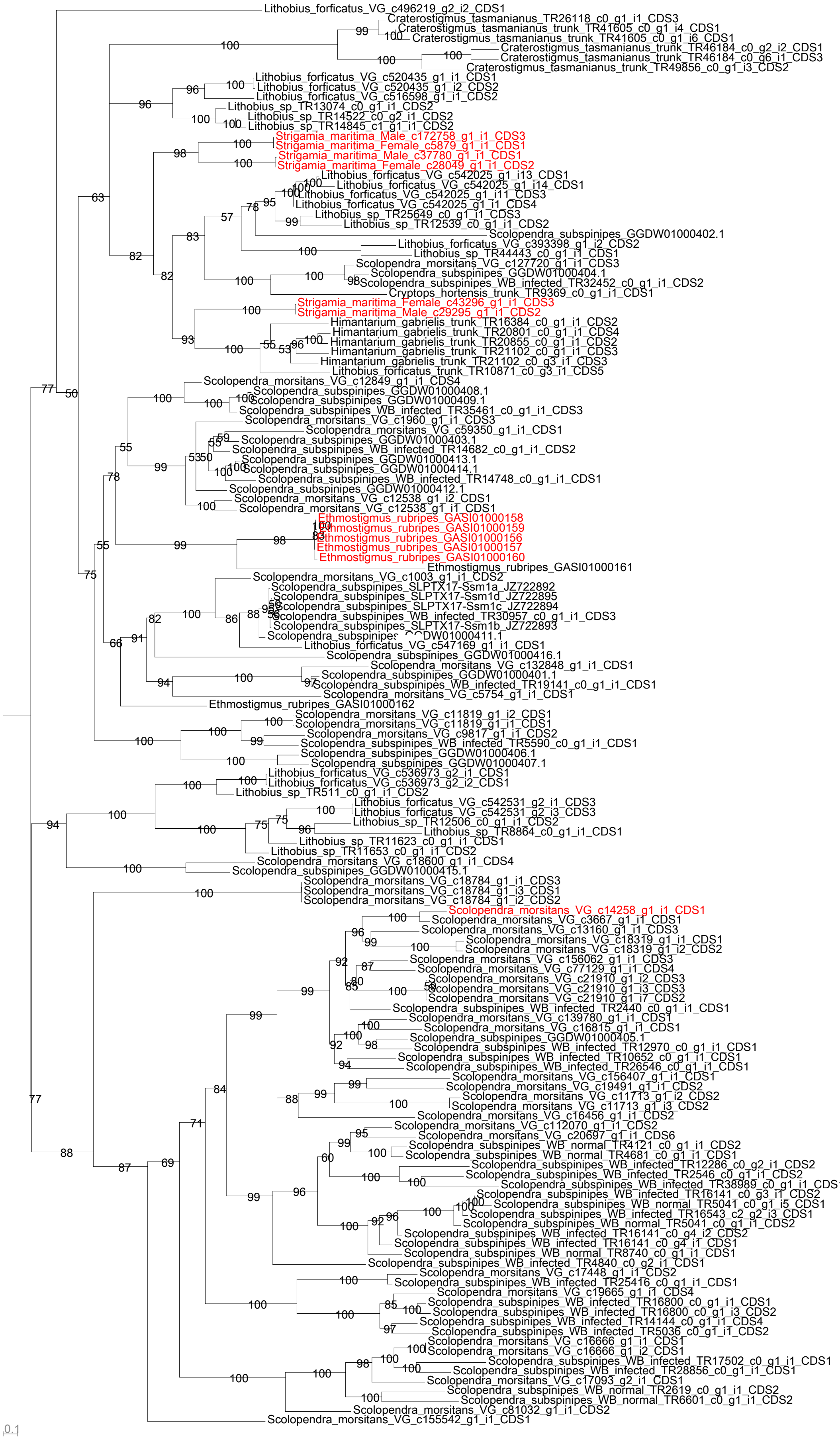

**Supplementary Figure S54:** Phylogenetic reconstruction of the SLPTX17 peptide family by ML under WAG+F+R3 (chosen according to BIC) displayed as mid-point rooted tree. Sequences identified in venom proteomes are coloured red. Bootstrap support values are shown at each node, and nodes with support < 50 are collapsed into multifurcations.

## Supplementary Figure S55

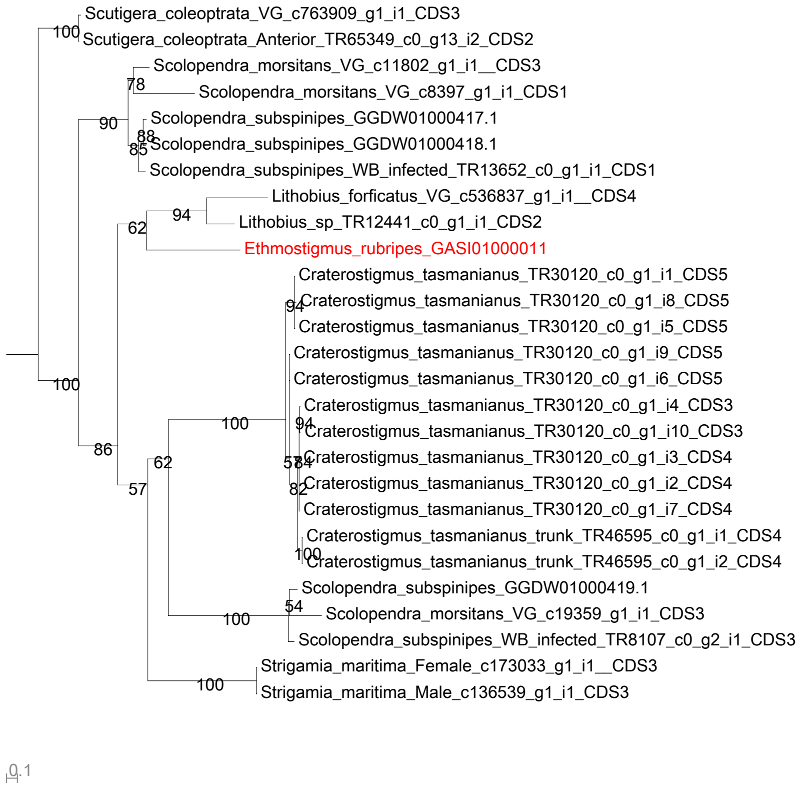


**Supplementary Figure S55:** Phylogenetic reconstruction of the SLPTX18 peptide family by ML under JTTDCMut+R2 (chosen according to BIC) displayed as mid-point rooted tree. Sequences identified in venom proteomes are coloured red. Bootstrap support values are shown at each node, and nodes with support < 50 are collapsed into multifurcations.

## Supplementary Figure S56

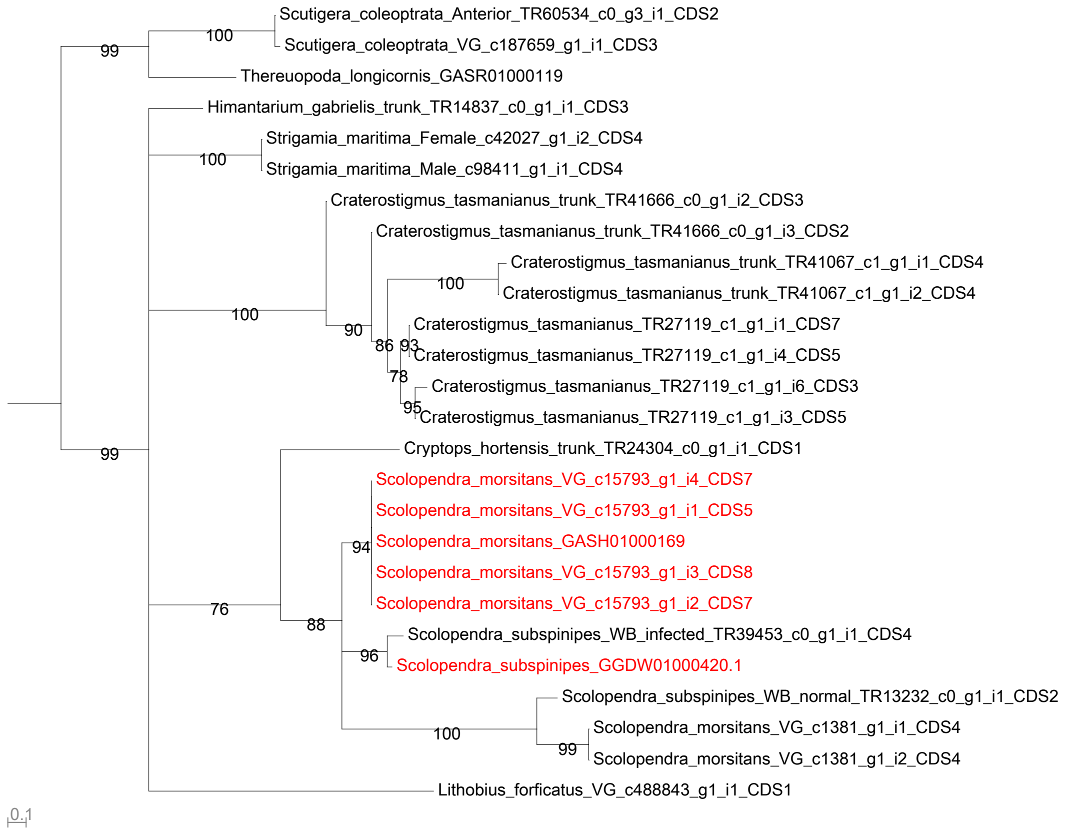


**Supplementary Figure S56:** Phylogenetic reconstruction of the SLPTX19 peptide family by ML under PMB+I+G4 (chosen according to BIC) displayed as mid-point rooted tree. Sequences identified in venom proteomes are coloured red. Bootstrap support values are shown at each node, and nodes with support < 50 are collapsed into multifurcations.

## Supplementary Figure S57

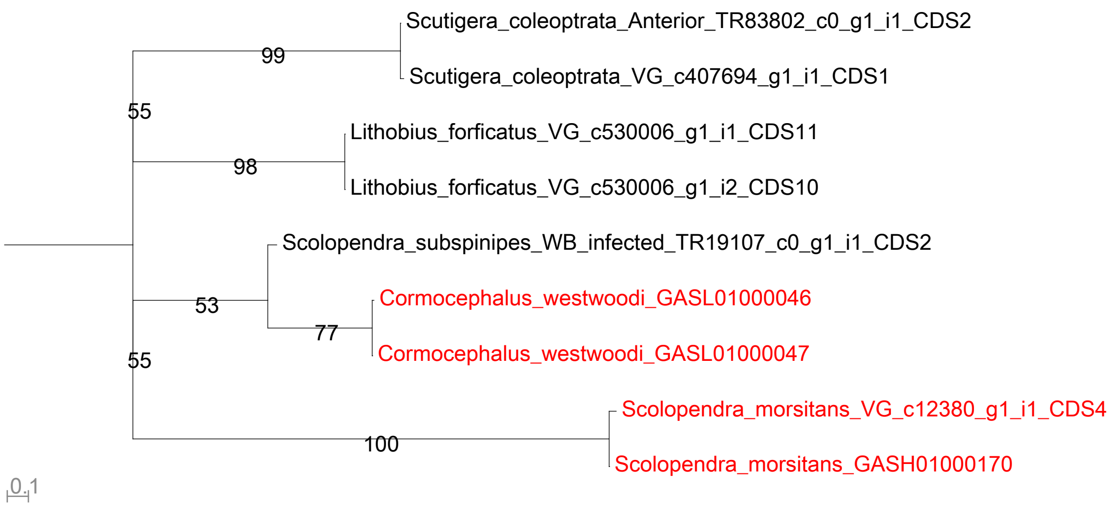


**Supplementary Figure S57:** Phylogenetic reconstruction of the SLPTX20 peptide family by ML under FLU+G4 (chosen according to BIC) displayed as mid-point rooted tree. Sequences identified in venom proteomes are coloured red. Bootstrap support values are shown at each node, and nodes with support < 50 are collapsed into multifurcations.

## Supplementary Figure S58

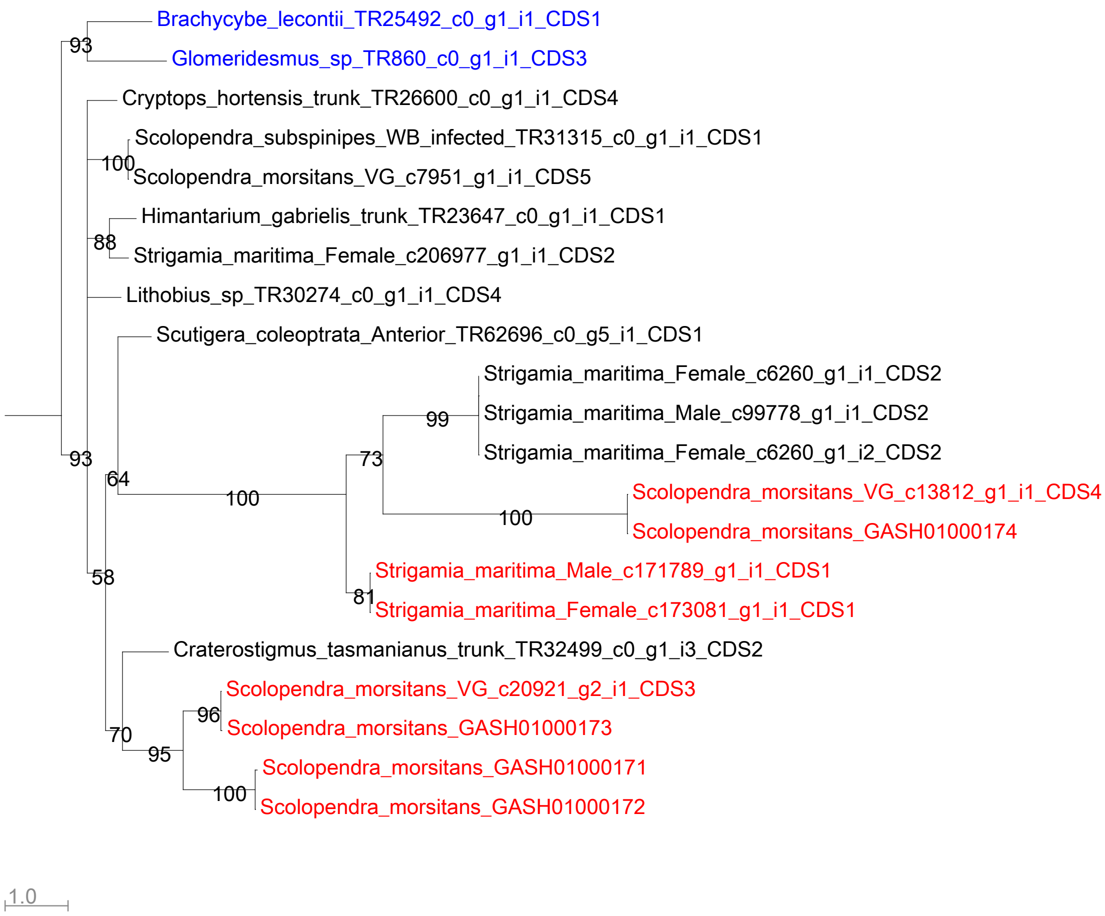


**Supplementary Figure S58:** Phylogenetic reconstruction of the SLPTX21 peptide family by ML under LG+G4 (chosen according to BIC) displayed as mid-point rooted tree. Sequences identified in venom proteomes are coloured red, while non-chilopod sequences are coloured blue. Bootstrap support values are shown at each node, and nodes with support < 50 are collapsed into multifurcations.

## Supplementary Figure S59

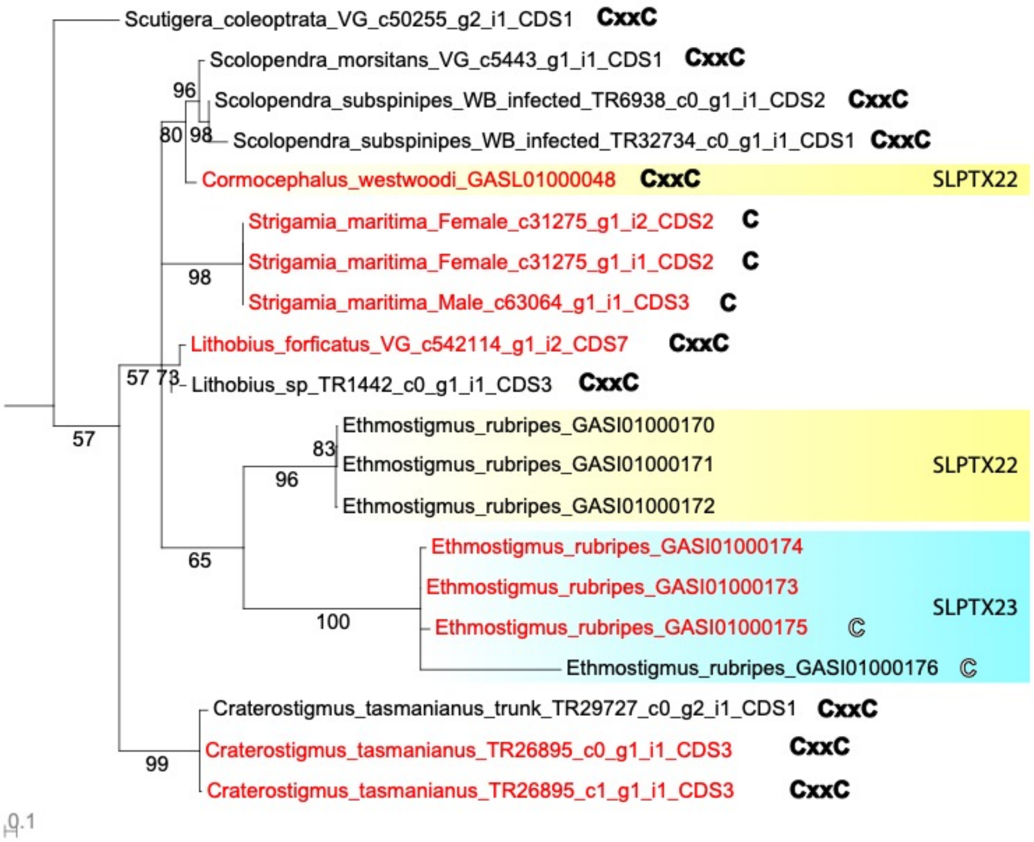


**Supplementary Figure S30:** Phylogenetic reconstruction of the SLPT22 and SLPTX23 families by ML under VT+G4 (chosen according to BIC) displayed as mid-point rooted tree. Sequences identified in venom proteomes are coloured red, previously assigned families are indicated by gradient coloured boxes, while the presence of homologous cysteines is indicated by solid "CxxC" (two cysteines) or "C" (single cysteine), and analogous cysteines by unfilled "C" (see alignment in Supplementary Material SS7). Bootstrap support values are shown at each node, and nodes with support < 50 are collapsed into multifurcations.

## Supplementary Figure S60

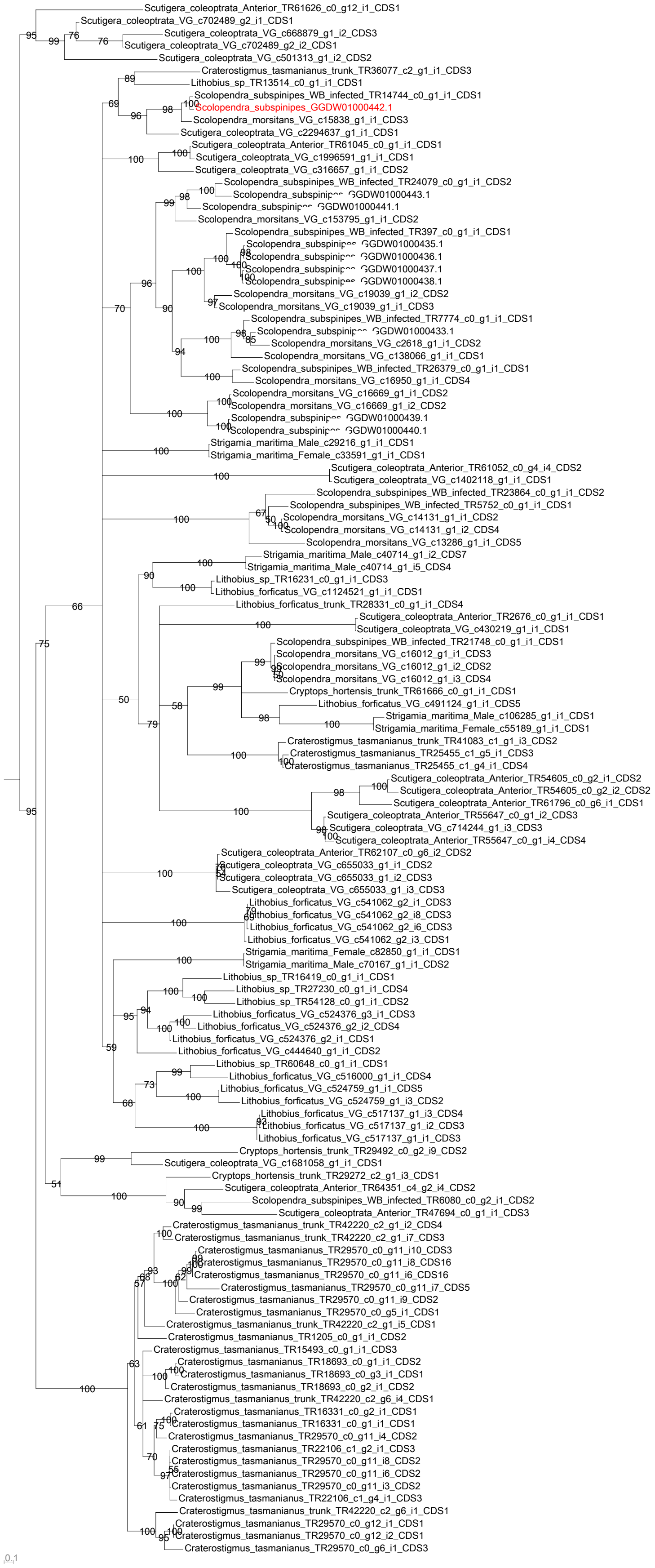

**Supplementary Figure S60:** Phylogenetic reconstruction of the SLPTX29 peptide family by ML under VT+R4 (chosen according to BIC) displayed as mid-point rooted tree. Sequences identified in venom proteomes are coloured red. Bootstrap support values are shown at each node, and nodes with support < 50 are collapsed into multifurcations.

## Supplementary Figure S61

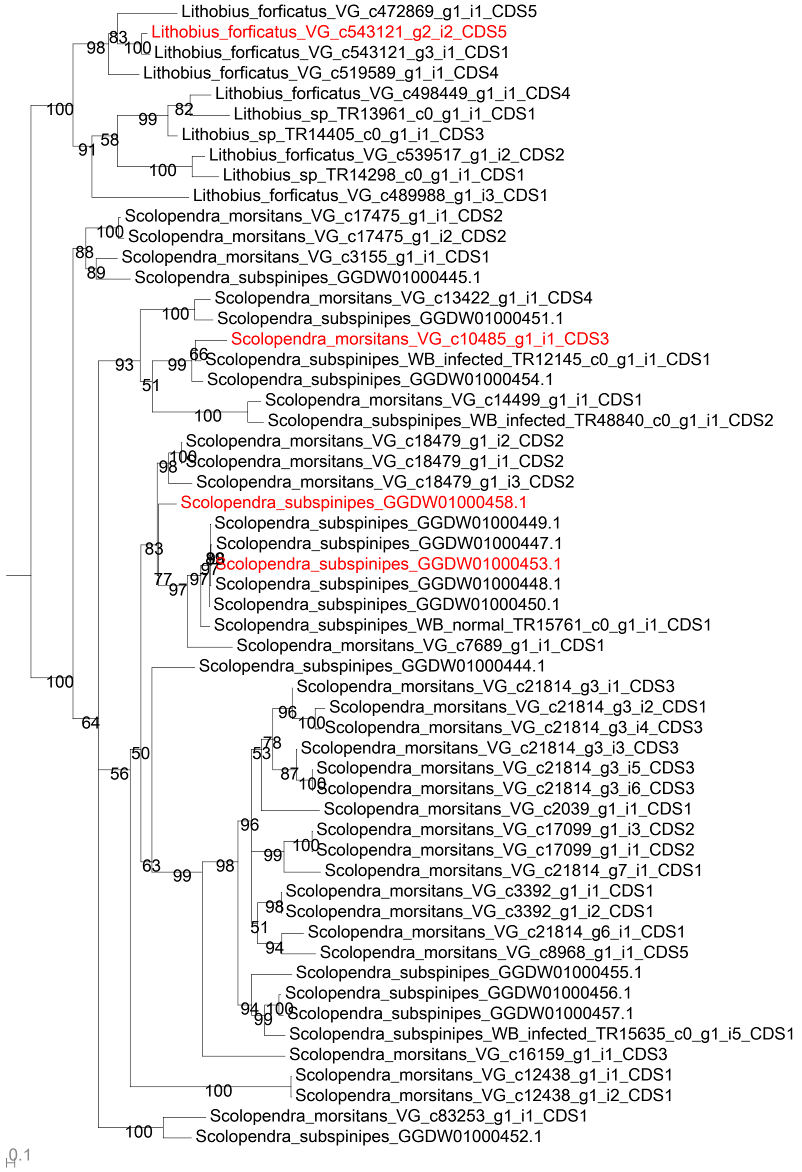


**Supplementary Figure S61:** Phylogenetic reconstruction of the SLPTX30 peptide family by ML under VT+R4 (chosen according to BIC) displayed as mid-point rooted tree. Sequences identified in venom proteomes are coloured red. Bootstrap support values are shown at each node, and nodes with support < 50 are collapsed into multifurcations.

## Supplementary Figure S62

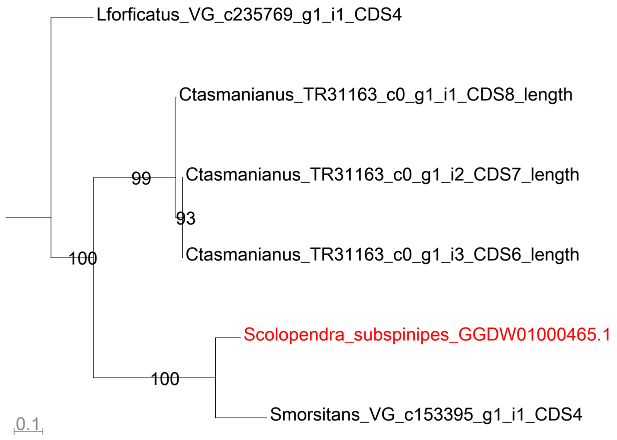


**Supplementary Figure S62:** Phylogenetic reconstruction of the centipede TGF-beta-like family by ML under FLU+I (chosen according to BIC) displayed as mid-point rooted tree. Sequences identified in venom proteomes are coloured red. Bootstrap support values are shown at each node, and nodes with support < 50 are collapsed into multifurcations.

## Supplementary Figure S63

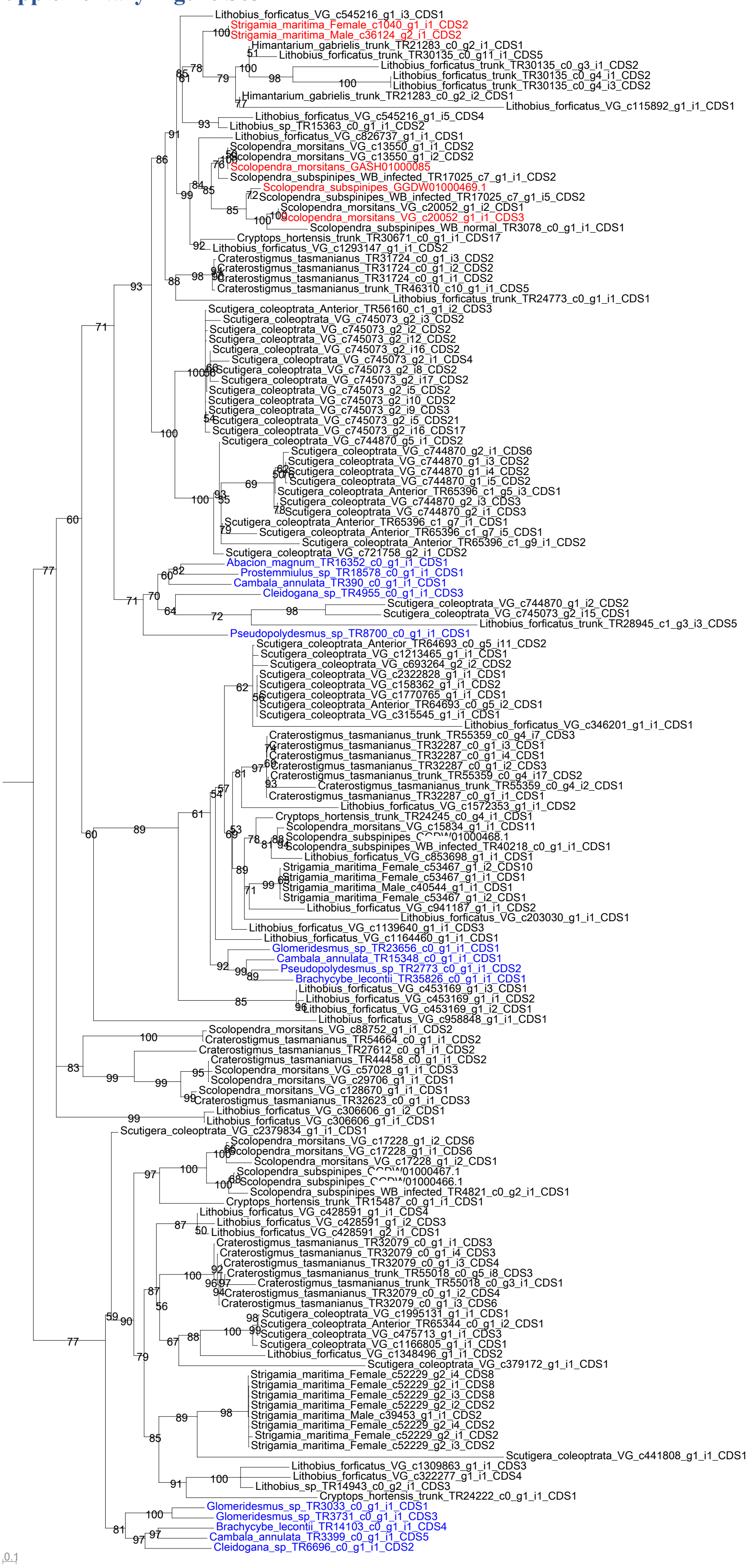

**Supplementary Figure S63:** Phylogenetic reconstruction of myriapod transferrins by ML under VT+R4 (chosen according to BIC) displayed as mid-point rooted tree. Sequences identified in venom proteomes are coloured red, while non-chilopod sequences are coloured blue. Bootstrap support values are shown at each node, and nodes with support < 50 are collapsed into multifurcations.

## Supplementary Figure S64

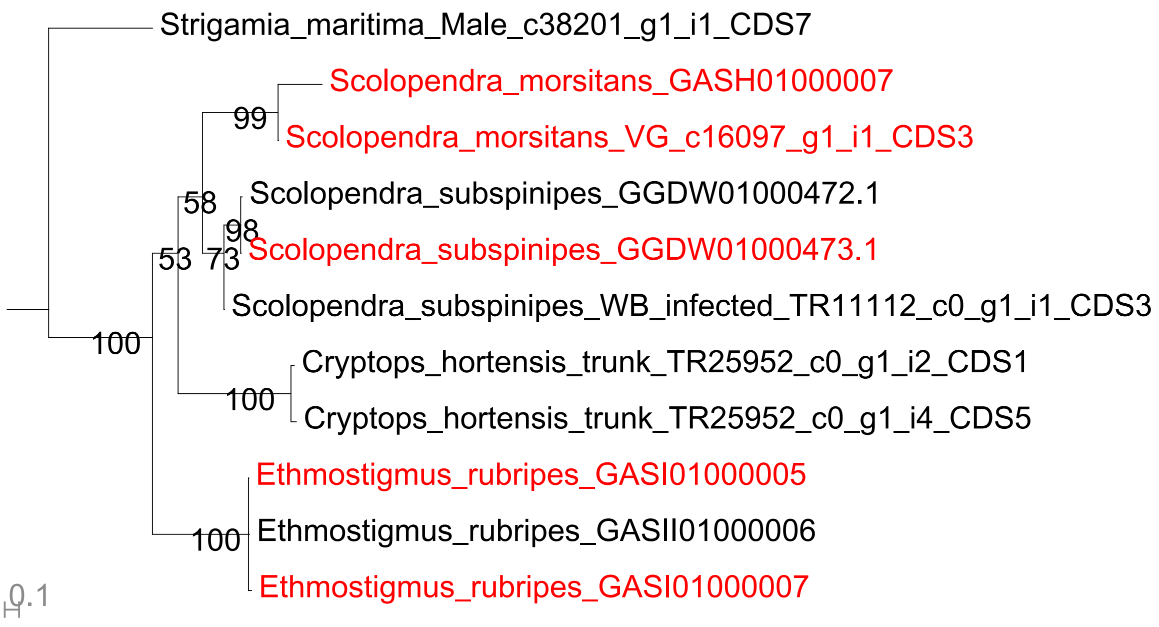


**Supplementary Figure S64:** Phylogenetic reconstruction of centipede uncharacterised protein family 5 by ML under VT+G4 (chosen according to BIC) displayed as mid-point rooted tree. Sequences identified in venom proteomes are coloured red. Bootstrap support values are shown at each node. Note the poor support for the nesting of *Cryptops* within scolopendrids.

## Supplementary Figure S65

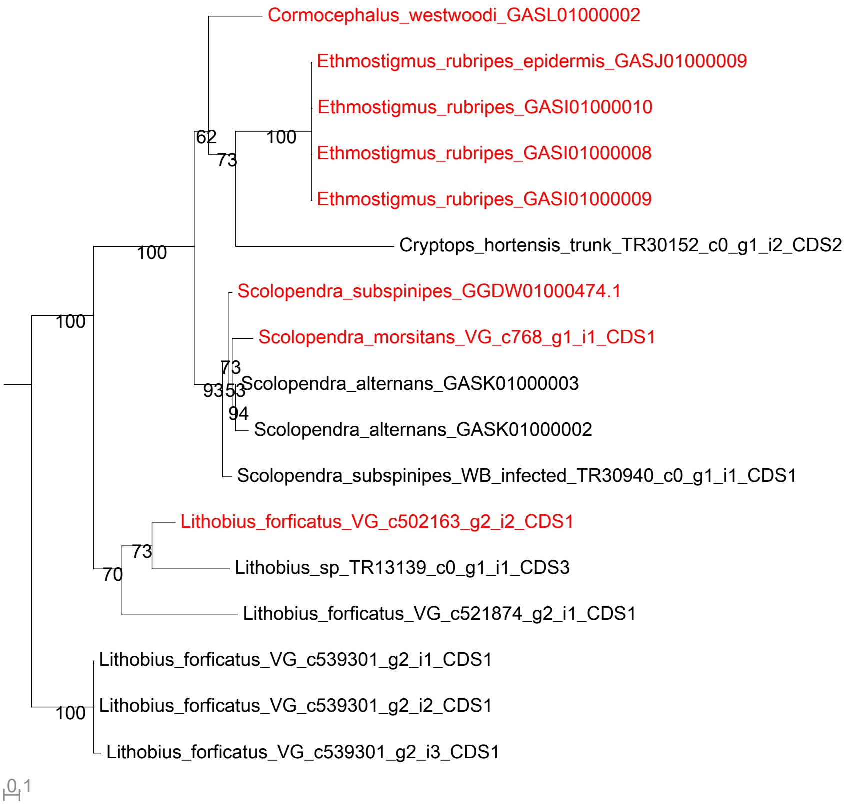


**Supplementary Figure S65:** Phylogenetic reconstruction of centipede uncharacterised protein family 6 by ML under VT+G4 (chosen according to BIC) displayed as mid-point rooted tree. Sequences identified in venom proteomes are coloured red. Bootstrap support values are shown at each node, and nodes with support < 50 are collapsed into multifurcations.

## Supplementary Figure S66

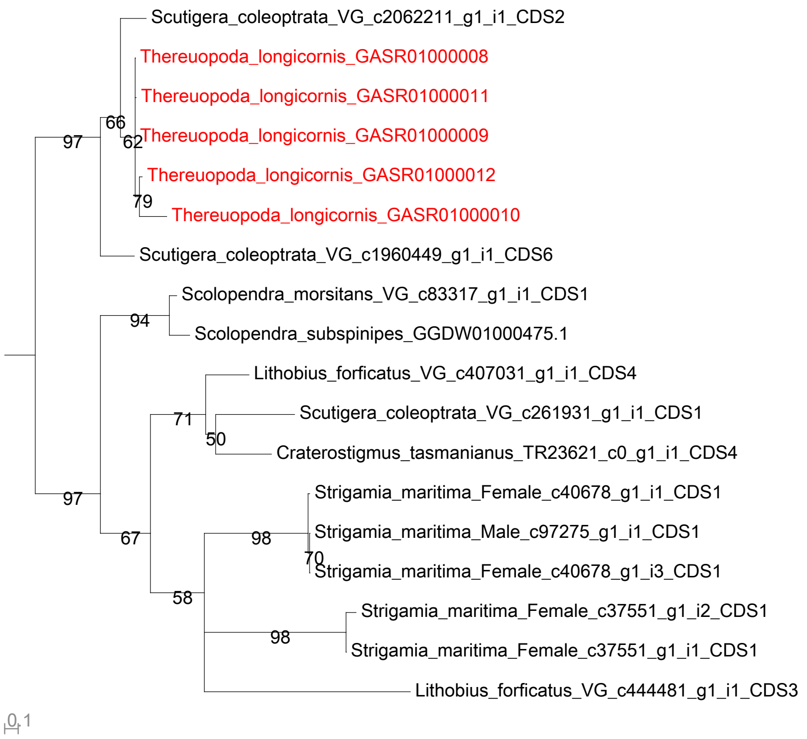


**Supplementary Figure S66:** Phylogenetic reconstruction of centipede uncharacterised protein family 8 by ML under VT+R2 (chosen according to BIC) displayed as mid-point rooted tree. Sequences identified in venom proteomes are coloured red. Bootstrap support values are shown at each node, and nodes with support < 50 are collapsed into multifurcations.

## Supplementary Figure S67

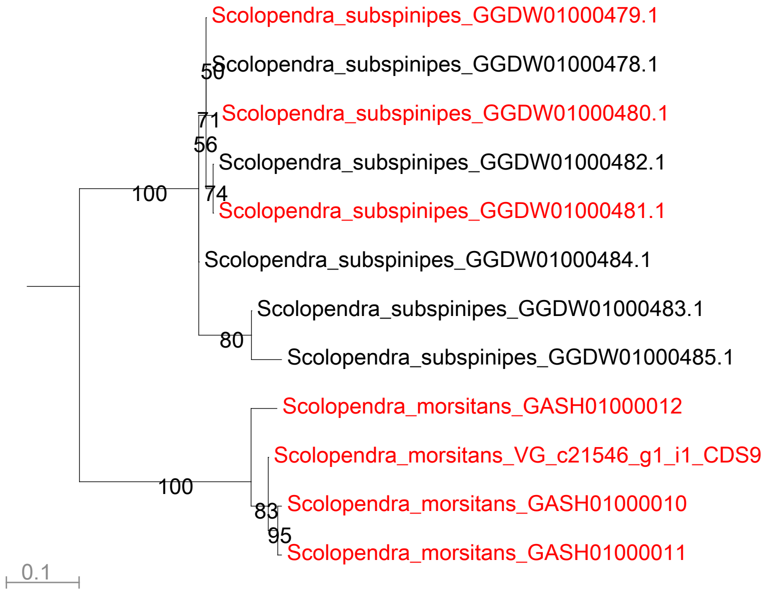


**Supplementary Figure S67:** Phylogenetic reconstruction of centipede uncharacterised protein family 10 by ML under JTT (chosen according to BIC) displayed as mid-point rooted tree. Sequences identified in venom proteomes are coloured red. Bootstrap support values are shown at each node, and nodes with support < 50 are collapsed into multifurcations.

### Supplementary Figure S68

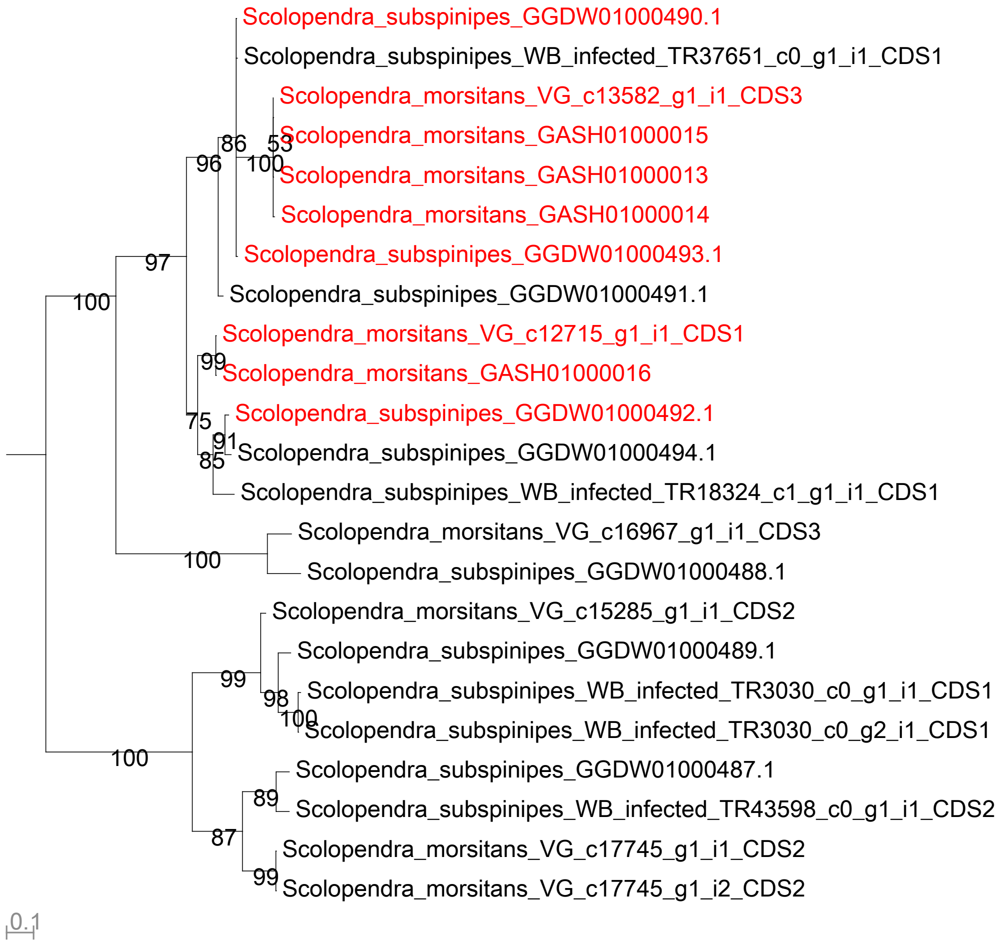


**Supplementary Figure S68:** Phylogenetic reconstruction of centipede uncharacterised protein family 11 by ML under WAG+G4 (chosen according to BIC) displayed as mid-point rooted tree. Sequences identified in venom proteomes are coloured red. Bootstrap support values are shown at each node, and nodes with support < 50 are collapsed into multifurcations.

## Supplementary Figure S69

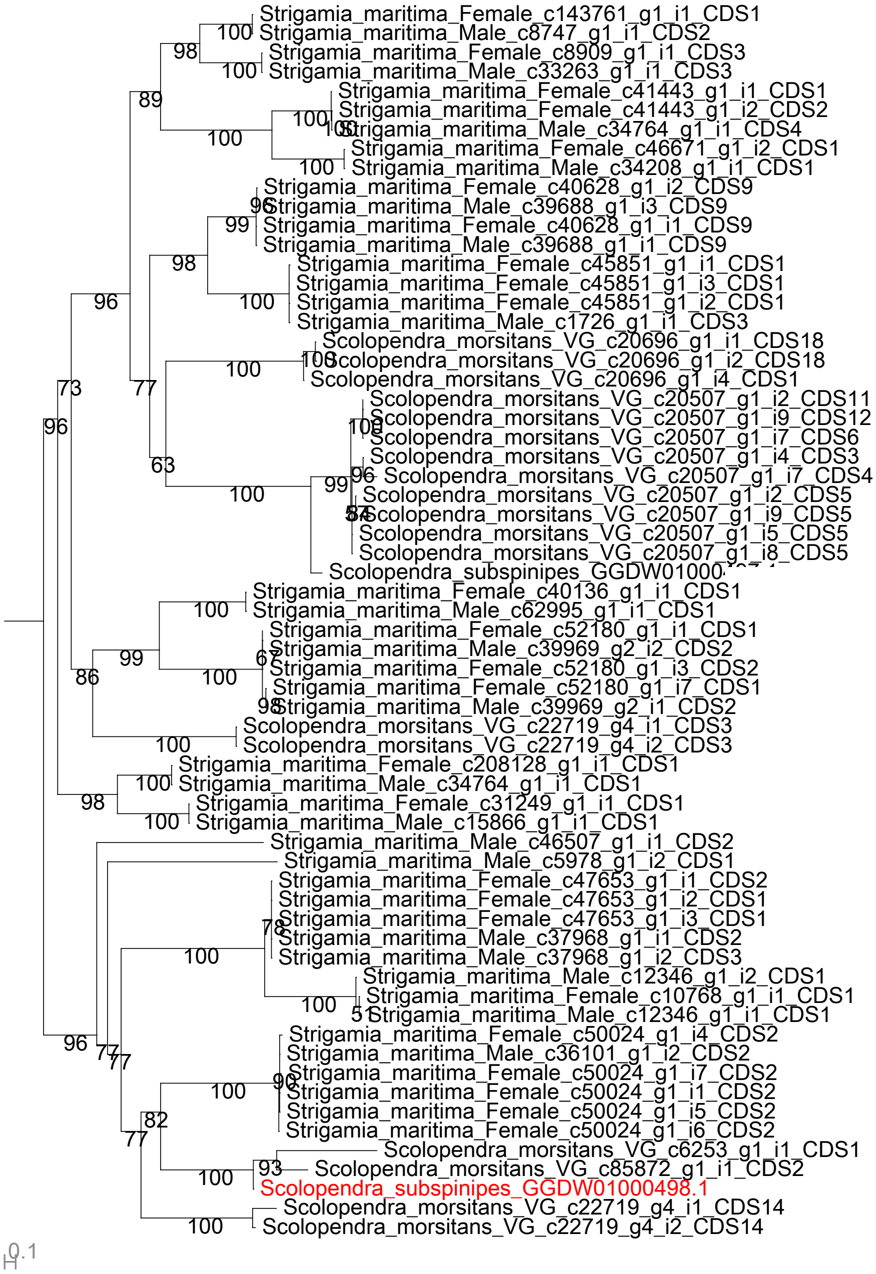


**Supplementary Figure S69:** Phylogenetic reconstruction of centipede uncharacterised protein family 12 by ML under VT+R3 (chosen according to BIC) displayed as mid-point rooted tree. Sequences identified in venom proteomes are coloured red. Bootstrap support values are shown at each node, and nodes with support < 50 are collapsed into multifurcations.

## Supplementary Figure S70

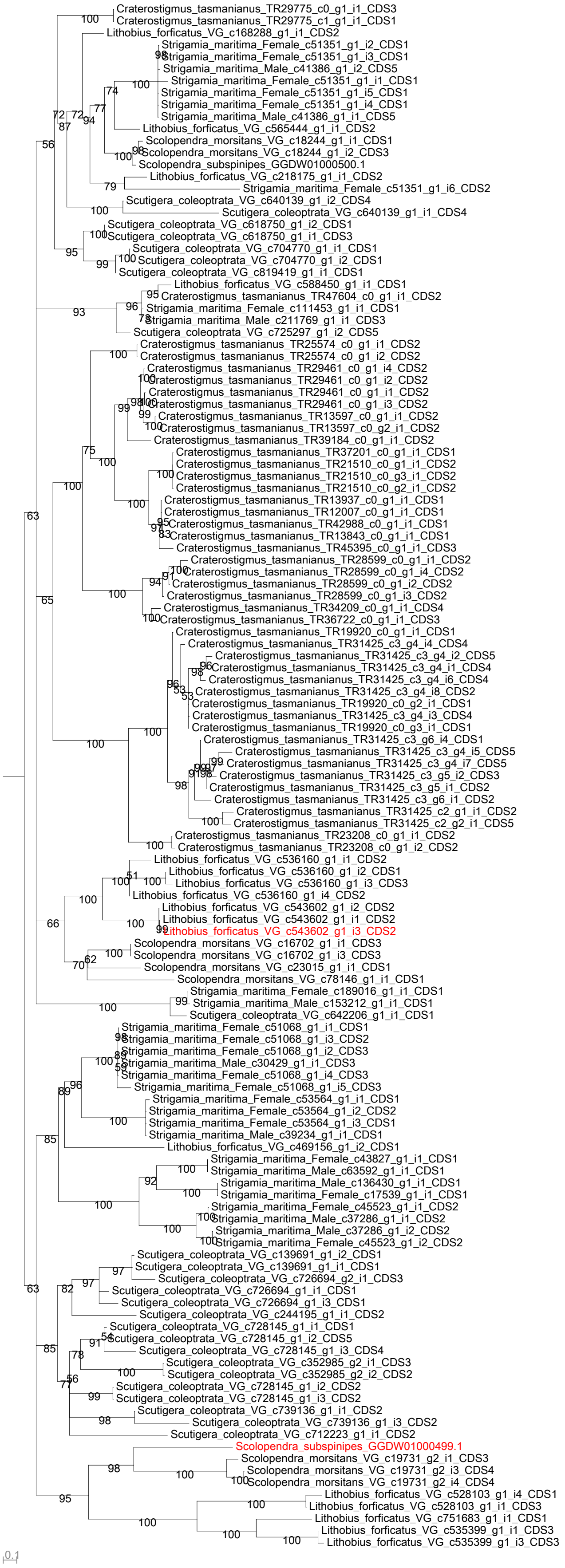

**Supplementary Figure S70:** Phylogenetic reconstruction of centipede uncharacterised protein family 13 by ML under WAG+F+I+G4 (chosen according to BIC) displayed as mid-point rooted tree. Sequences identified in venom proteomes are coloured red. Bootstrap support values are shown at each node, and nodes with support < 50 are collapsed into multifurcations.

## Supplementary Figure S71

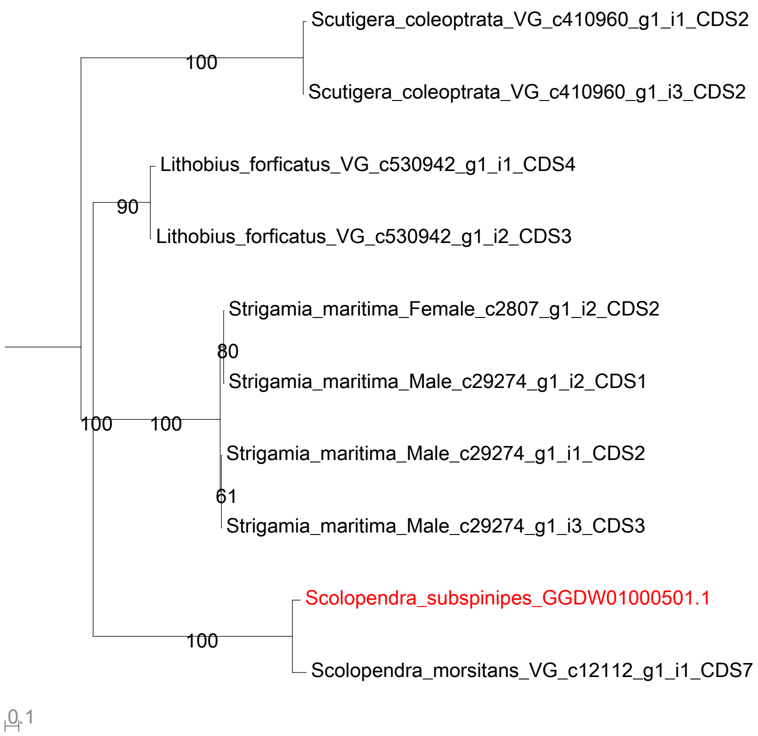


**Supplementary Figure S71:** Phylogenetic reconstruction of centipede uncharacterised protein family 14 by ML under JTT+G4 (chosen according to BIC) displayed as mid-point rooted tree. Sequences identified in venom proteomes are coloured red. Bootstrap support values are shown at each node, and nodes with support < 50 are collapsed into multifurcations.

.

## Supplementary Figure S72

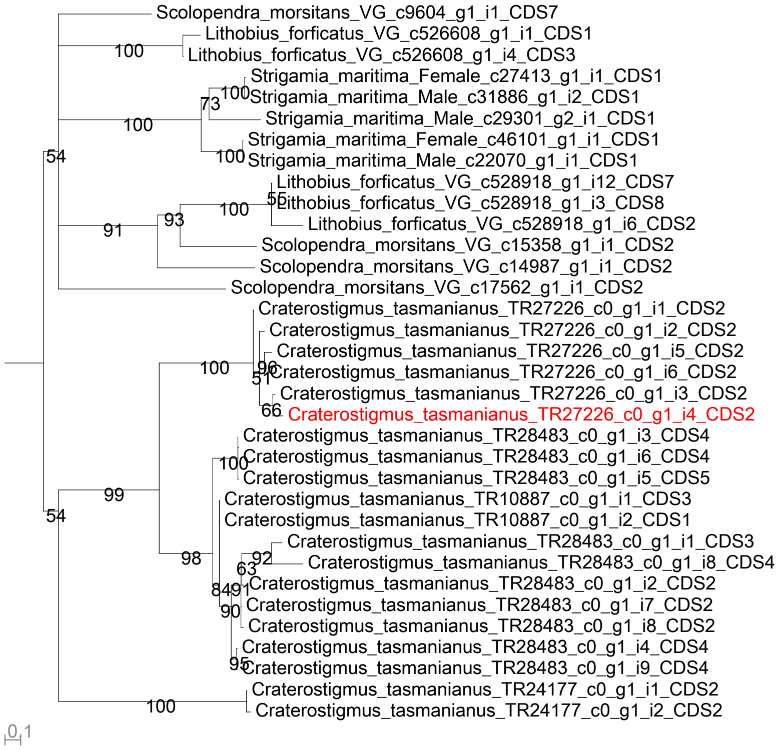


**Supplementary Figure S72:** Phylogenetic reconstruction of centipede uncharacterised protein family 16 by ML under PMB+I+G4 (chosen according to BIC) displayed as mid-point rooted tree. Sequences identified in venom proteomes are coloured red. Bootstrap support values are shown at each node, and nodes with support < 50 are collapsed into multifurcations.
