# Supplementary material for: Parallel Evolution of Complex Centipede Venoms Revealed by Comparative Proteotranscriptomic Analyses
Source: Mol Biol Evol. 2019 Aug 8;36(12):2748–63. doi: 10.1093/molbev/msz181 (PMC6878950; doi:10.1093/molbev/msz181)
Supplement: msz181_Supplementary_Data [file msz181_supplementary_data.zip › Supplementary_Material_S7.pdf]

Supplementary Material S9

The following trees depict the functional recruitments and losses of toxin families across the species tree based on ACCTRAN (Accelerated Transformation) and DELTRAN (Delayed Transformation) parsimony optimizations. See the main article for abbreviations of species names. Recruitments are shown in green and losses in red, with 'T' denoting the recruitment or loss of the toxin family from transcriptomes and 'P' denoting recruitment or loss of the toxin family from the venom proteomes. Where ACCTRAN and DELTRAN optimizations differ both are shown.

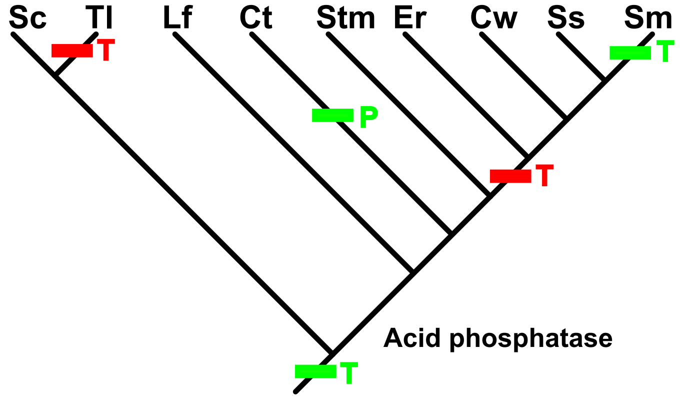
Sc
Tl
Lf
Ct
Stm
Er
Cw
Ss
Sm
T
P
T
T
T
Acid phosphatase

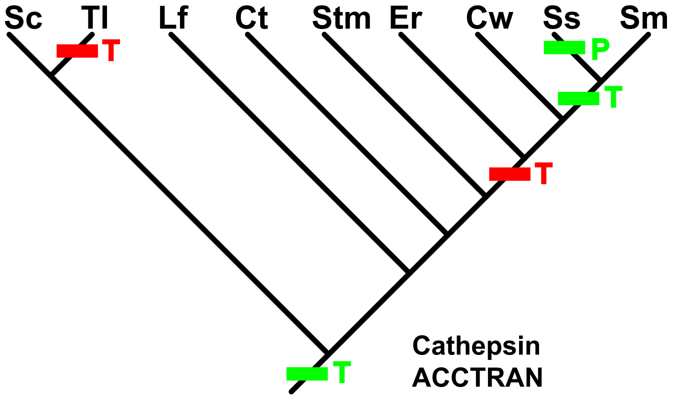
Sc
Tl
Lf
Ct
Stm
Er
Cw
Ss
Sm
T
P
T
T
T
Cathepsin
ACCTRAN

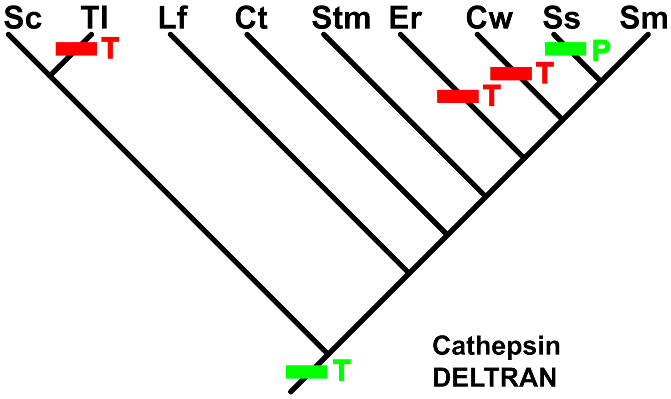
Sc
Tl
Lf
Ct
Stm
Er
Cw
Ss
Sm
T
P
T
T
T
Cathepsin
DELTRAN

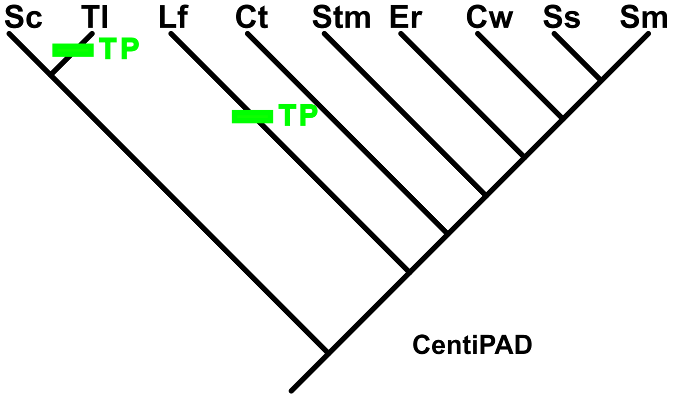
Sc
Tl
Lf
Ct
Stm
Er
Cw
Ss
Sm
TP
TP
CentiPAD

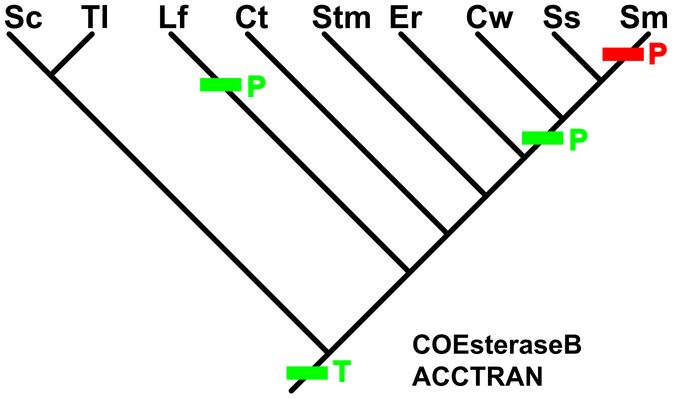
Sc
Tl
Lf
Ct
Stm
Er
Cw
Ss
Sm
P
P
P
T
COEsteraseB
ACCTRAN

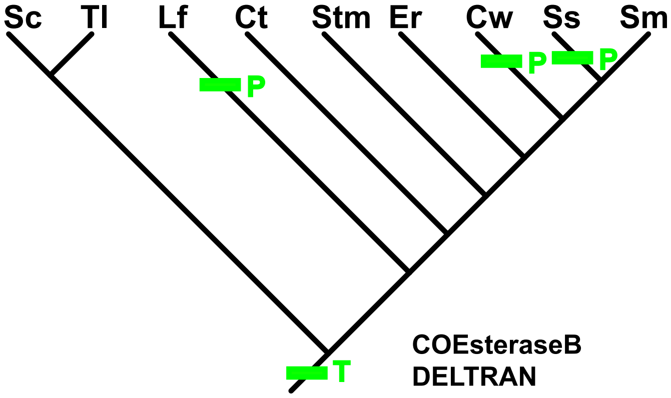
Sc
Tl
Lf
Ct
Stm
Er
Cw
Ss
Sm
P
P
P
T
COEsteraseB
DELTRAN

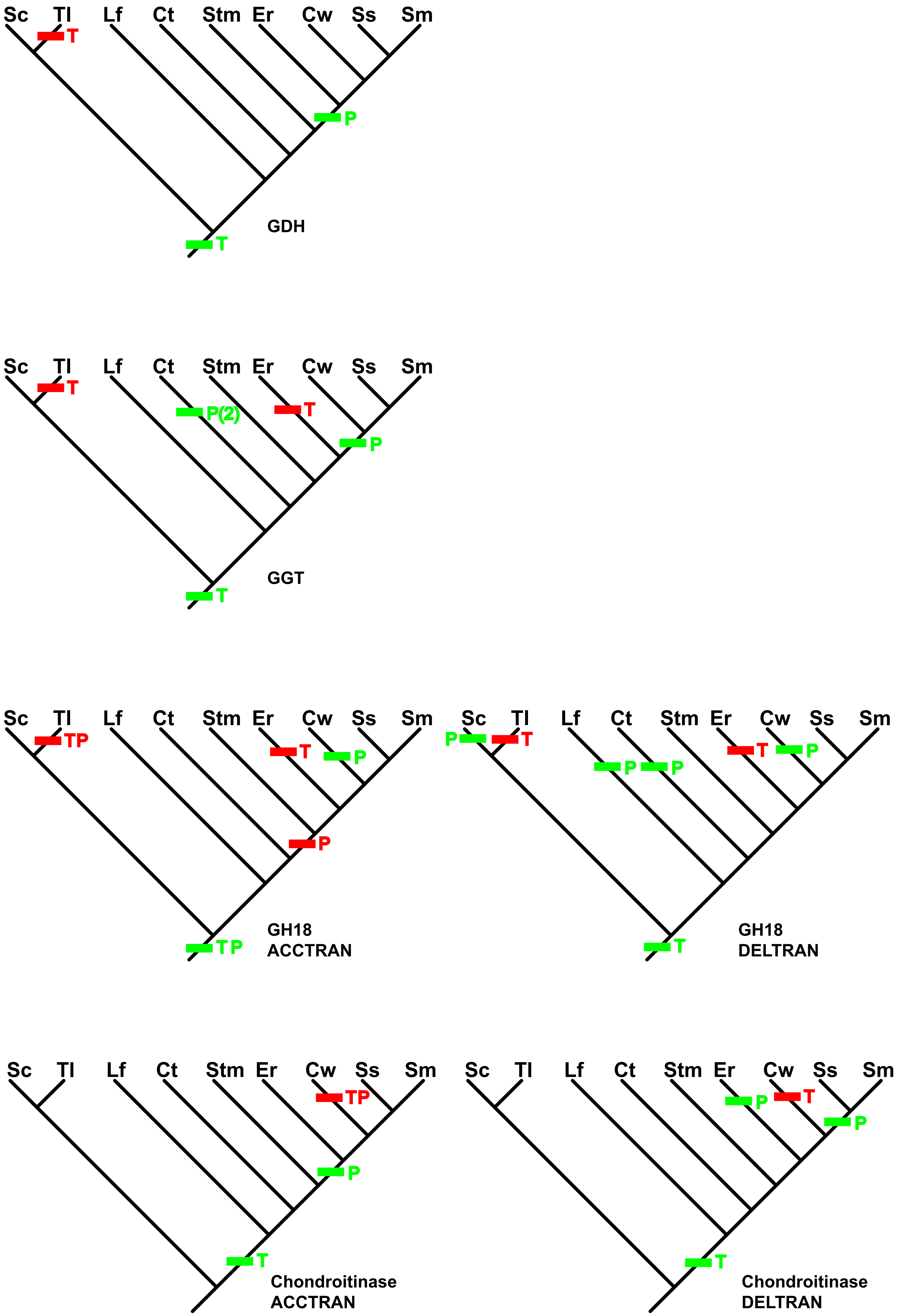
Sc Tl Lf Ct Stm Er Cw Ss Sm
T
P
T
GDH
Sc Tl Lf Ct Stm Er Cw Ss Sm
T
P(2)
T
P
T
GGT
Sc Tl Lf Ct Stm Er Cw Ss Sm
TP
T
P
P
TP
GH18
ACCTRAN
Sc Tl Lf Ct Stm Er Cw Ss Sm
P
T
P
P
T
P
T
GH18
DELTRAN
Sc Tl Lf Ct Stm Er Cw Ss Sm
TP
P
T
Chondroitinase
ACCTRAN
Sc Tl Lf Ct Stm Er Cw Ss Sm
P
T
P
T
Chondroitinase
DELTRAN

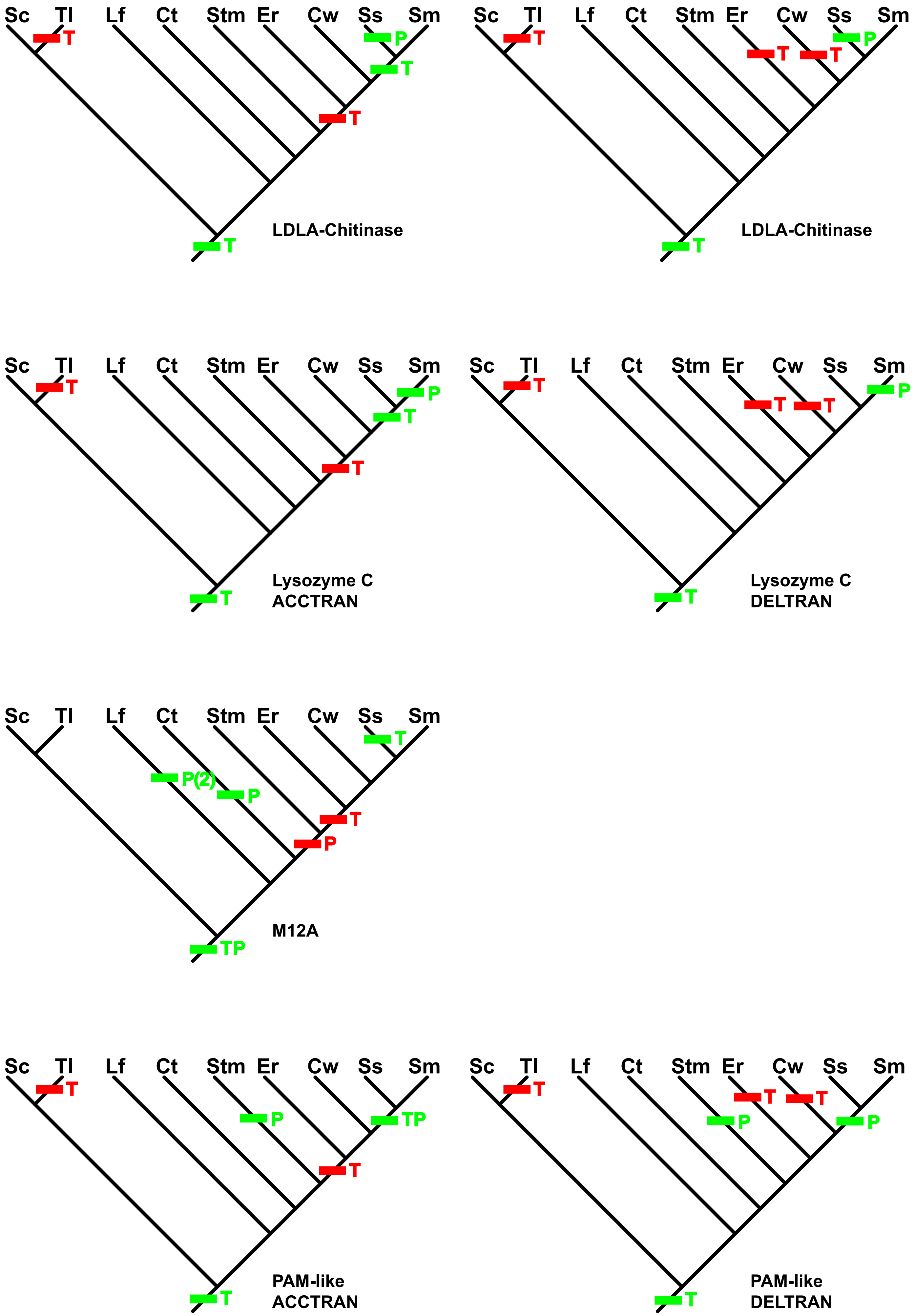
Sc Tl Lf Ct Stm Er Cw Ss Sm
T P T T T
LDLA-Chitinase
Sc Tl Lf Ct Stm Er Cw Ss Sm
T T T P T
LDLA-Chitinase
Sc Tl Lf Ct Stm Er Cw Ss Sm
T P T T T
Lysozyme C
ACCTRAN
Sc Tl Lf Ct Stm Er Cw Ss Sm
T T T P T
Lysozyme C
DELTRAN
Sc Tl Lf Ct Stm Er Cw Ss Sm
T P(2) P T P TP
M12A
Sc Tl Lf Ct Stm Er Cw Ss Sm
T P TP T T
PAM-like
ACCTRAN
Sc Tl Lf Ct Stm Er Cw Ss Sm
T T T P P T
PAM-like
DELTRAN

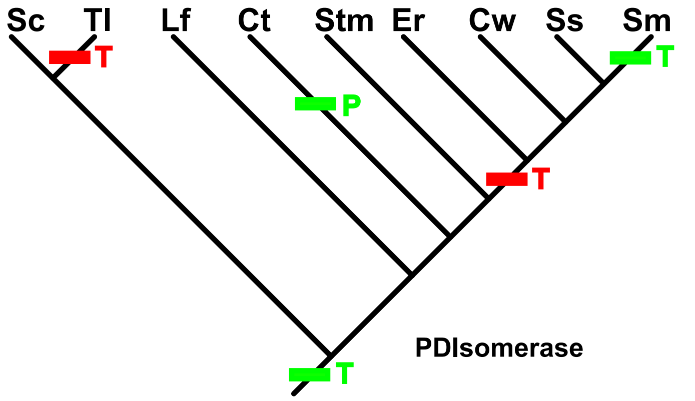
Sc Tl Lf Ct Stm Er Cw Ss Sm
T
P
T
T
T
PDIsomerase

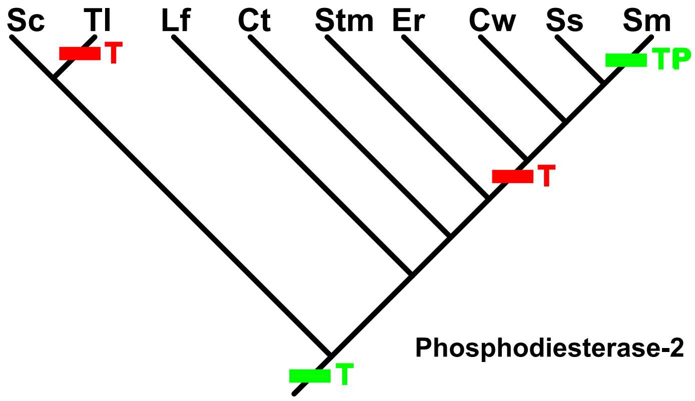
Sc Tl Lf Ct Stm Er Cw Ss Sm
T
TP
T
T
Phosphodiesterase-2

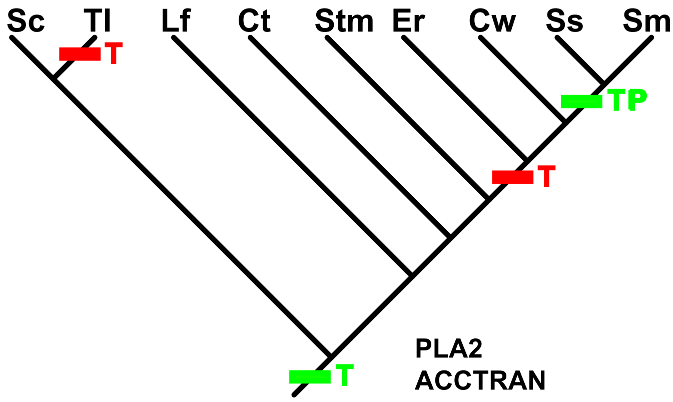
Sc Tl Lf Ct Stm Er Cw Ss Sm
T
TP
T
T
PLA2
ACCTRAN

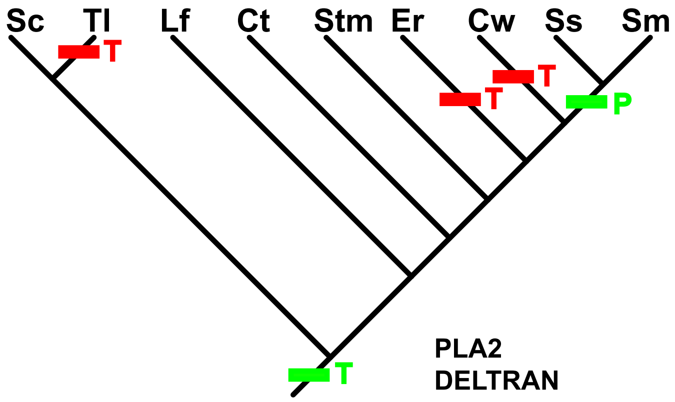
Sc Tl Lf Ct Stm Er Cw Ss Sm
T
T
T
P
T
PLA2
DELTRAN

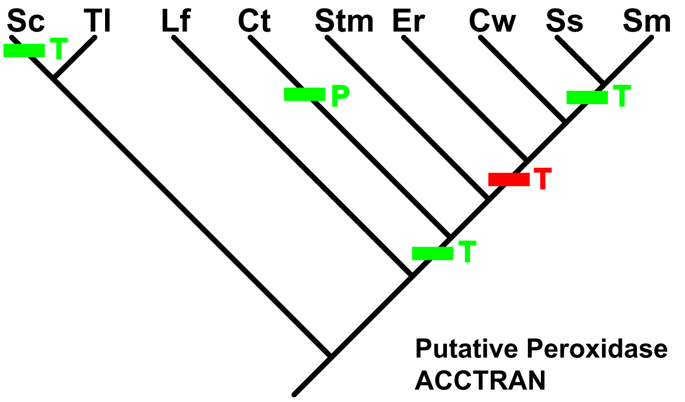
Sc Tl Lf Ct Stm Er Cw Ss Sm
T
P
T
T
T
Putative Peroxidase
ACCTRAN

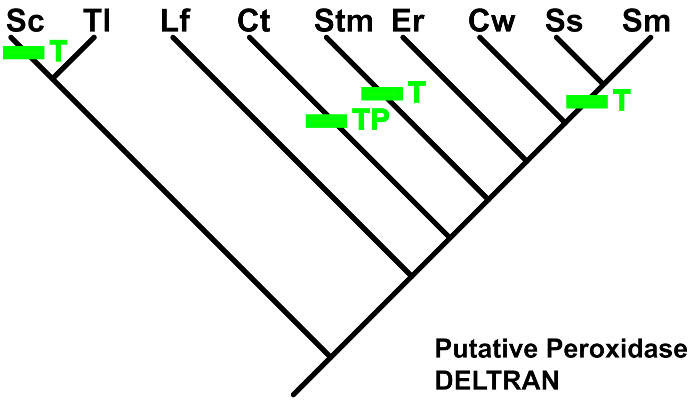
Sc Tl Lf Ct Stm Er Cw Ss Sm
T
T
TP
T
Putative Peroxidase
DELTRAN

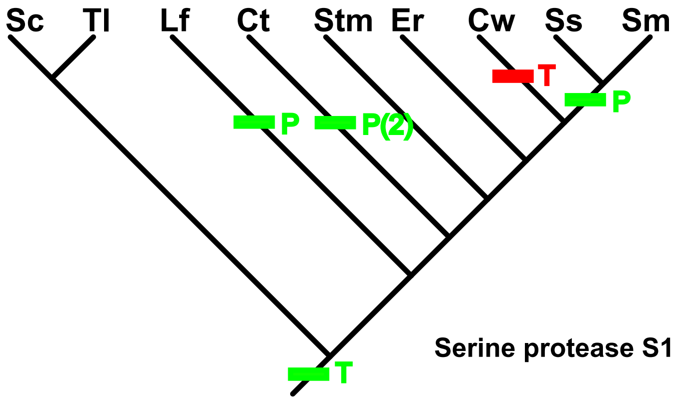

Serine protease S1

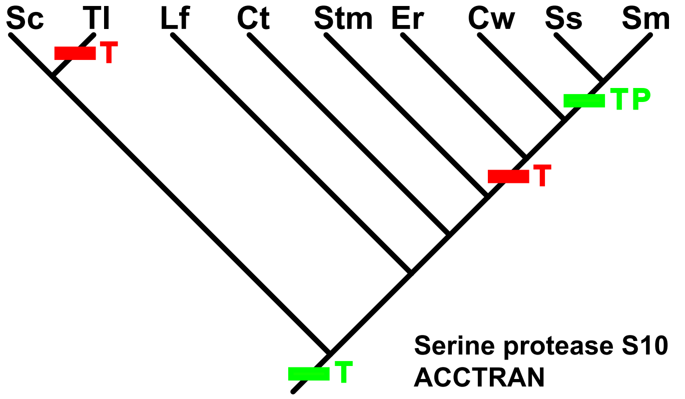

Serine protease S10
ACCTRAN

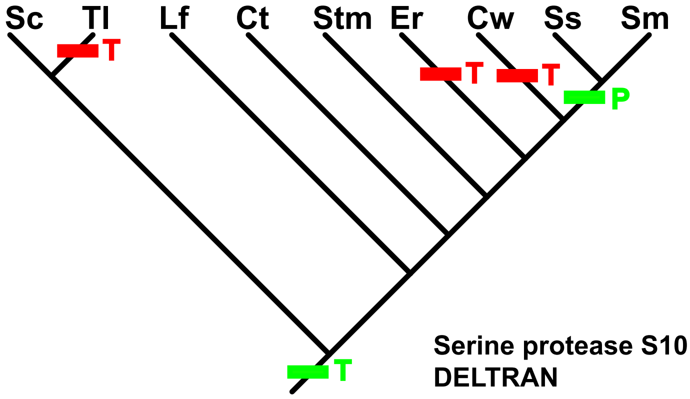

Serine protease S10
DELTRAN

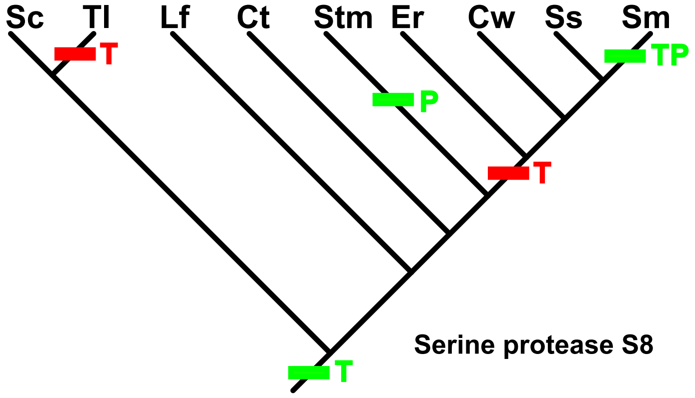

Serine protease S8

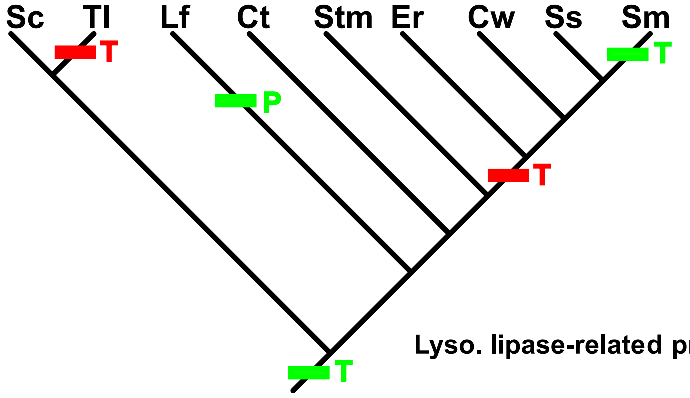

Lyso. lipase-related prot.

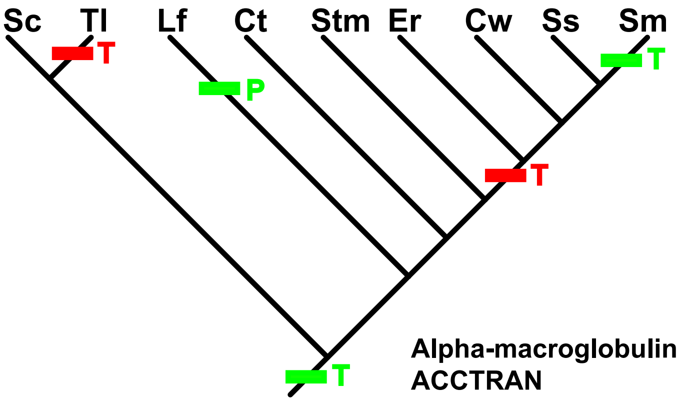

Alpha-macroglobulin-like
ACCTRAN

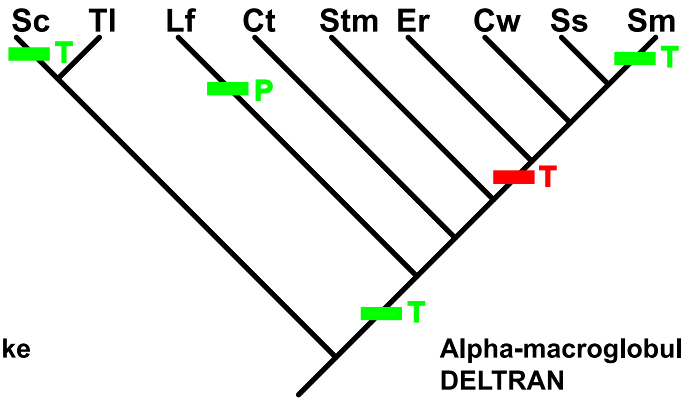

Alpha-macroglobulin-like
DELTRAN

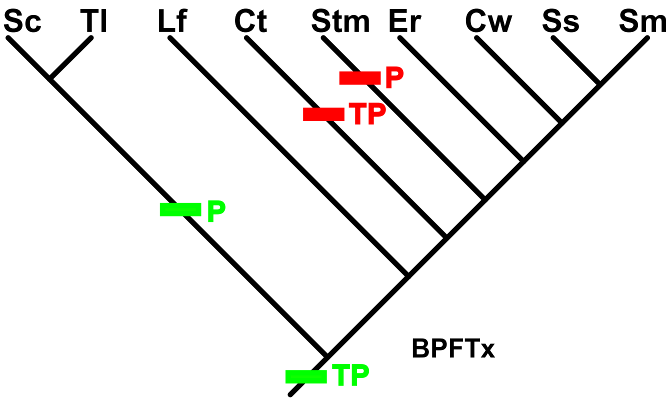

BPFTx

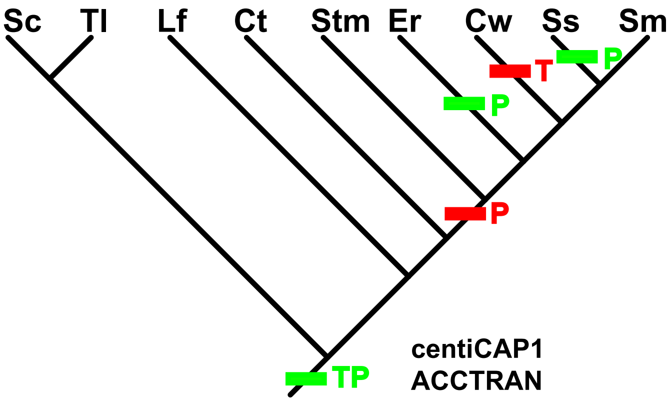

centiCAP1
ACCTRAN

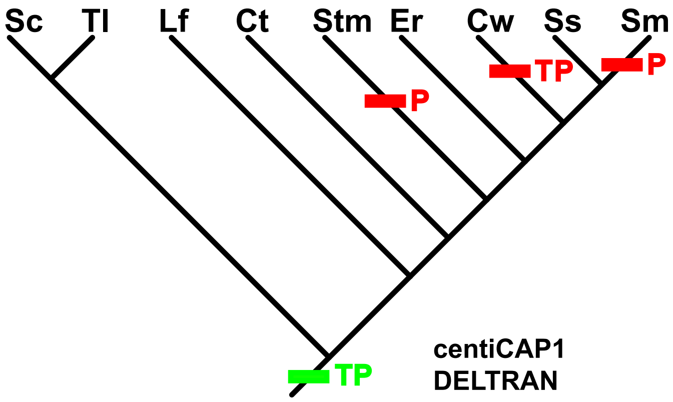

centiCAP1
DELTRAN

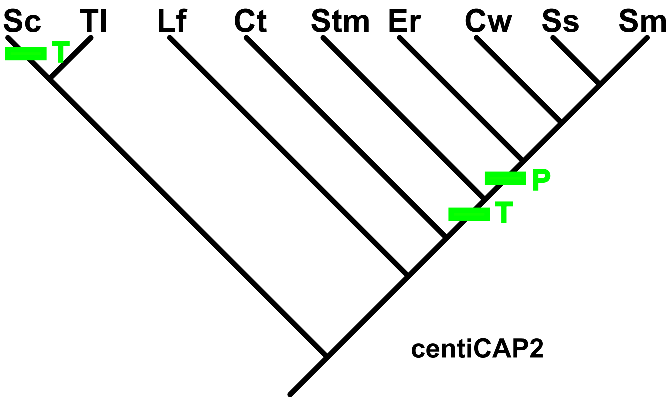

centiCAP2

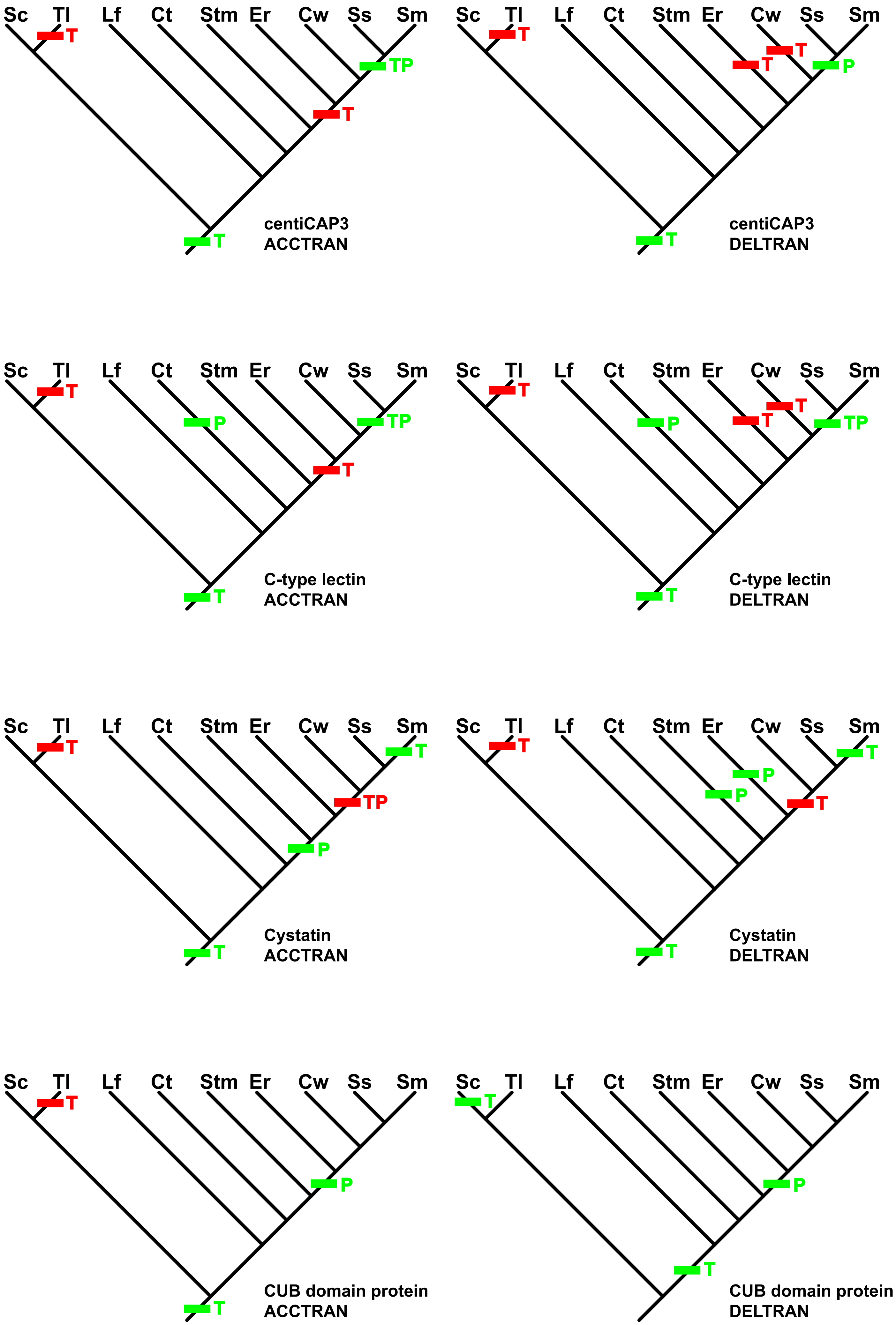
Sc Tl Lf Ct Stm Er Cw Ss Sm
T
TP
T
T
centiCAP3
ACCTRAN
Sc Tl Lf Ct Stm Er Cw Ss Sm
T
T
T
P
T
centiCAP3
DELTRAN
Sc Tl Lf Ct Stm Er Cw Ss Sm
T
P
TP
T
T
C-type lectin
ACCTRAN
Sc Tl Lf Ct Stm Er Cw Ss Sm
T
P
T
T
TP
T
C-type lectin
DELTRAN
Sc Tl Lf Ct Stm Er Cw Ss Sm
T
T
TP
P
T
Cystatin
ACCTRAN
Sc Tl Lf Ct Stm Er Cw Ss Sm
T
T
P
P
T
T
Cystatin
DELTRAN
Sc Tl Lf Ct Stm Er Cw Ss Sm
T
P
T
CUB domain protein
ACCTRAN
Sc Tl Lf Ct Stm Er Cw Ss Sm
T
P
T
CUB domain protein
DELTRAN

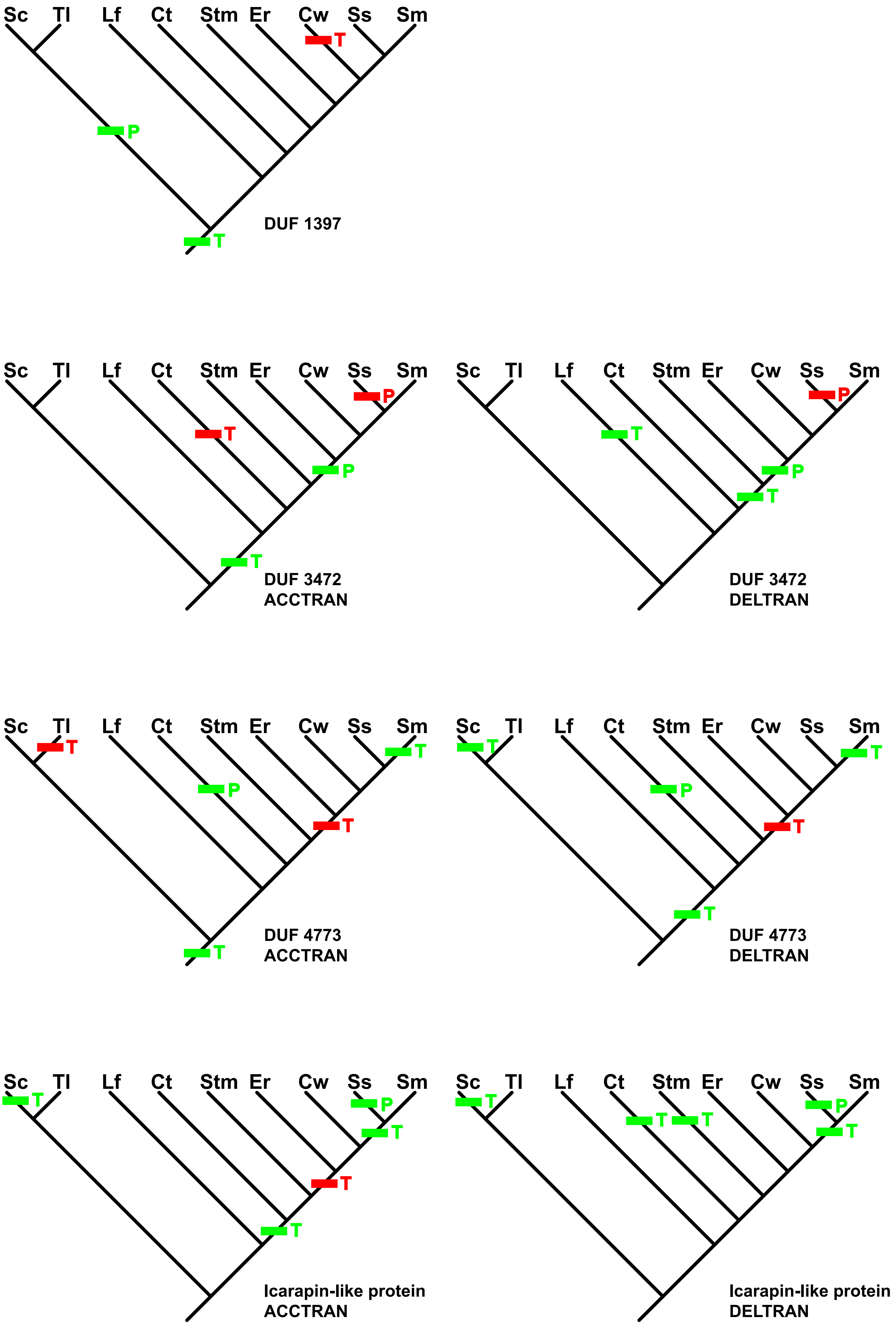

Sc Tl Lf Ct Stm Er Cw Ss Sm
T
P
T
DUF 1397
Sc Tl Lf Ct Stm Er Cw Ss Sm
P
T
P
T
DUF 3472
ACCTRAN
Sc Tl Lf Ct Stm Er Cw Ss Sm
P
T
P
T
DUF 3472
DELTRAN
Sc Tl Lf Ct Stm Er Cw Ss Sm
T
T
P
T
T
DUF 4773
ACCTRAN
Sc Tl Lf Ct Stm Er Cw Ss Sm
T
T
P
T
T
DUF 4773
DELTRAN
Sc Tl Lf Ct Stm Er Cw Ss Sm
T
P
T
T
T
Icarapin-like protein
ACCTRAN
Sc Tl Lf Ct Stm Er Cw Ss Sm
T
T
T
P
T
Icarapin-like protein
DELTRAN

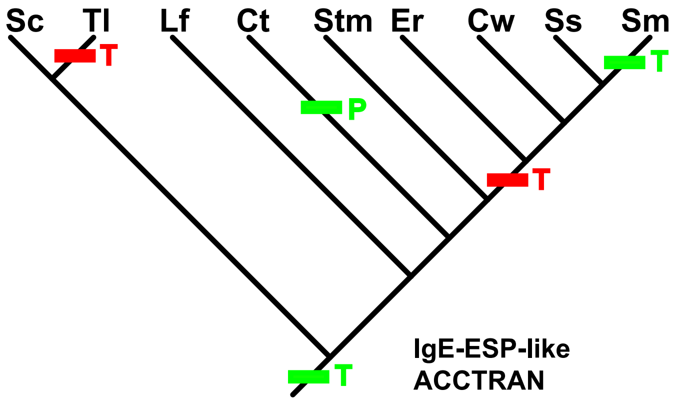

IgE-ESP-like
ACCTRAN

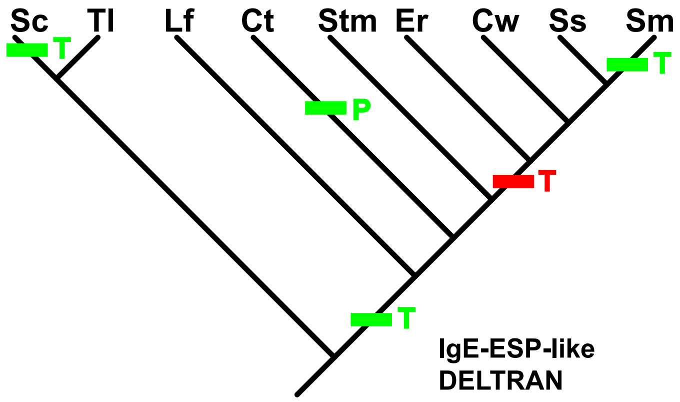

IgE-ESP-like
DELTRAN

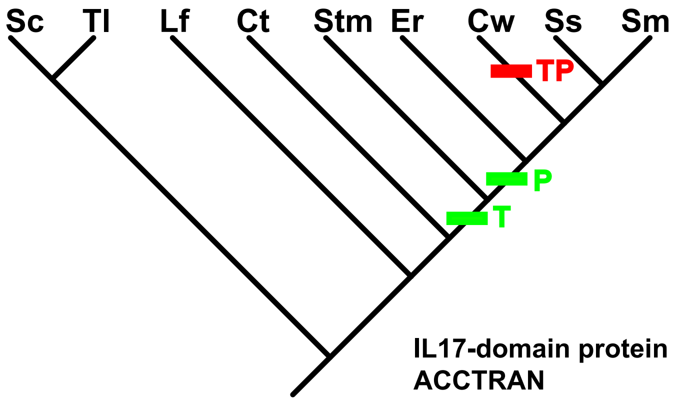

IL17-domain protein
ACCTRAN

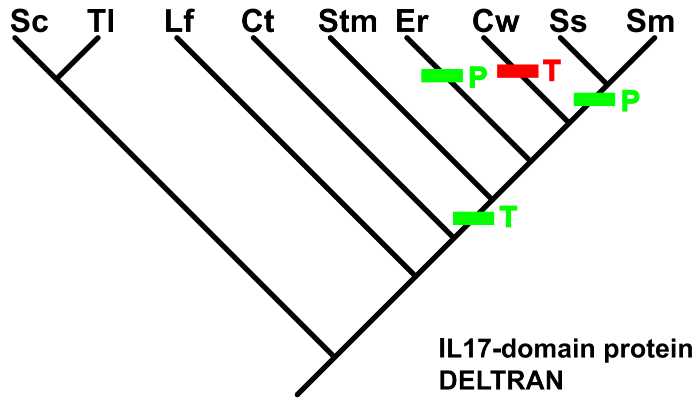

IL17-domain protein
DELTRAN

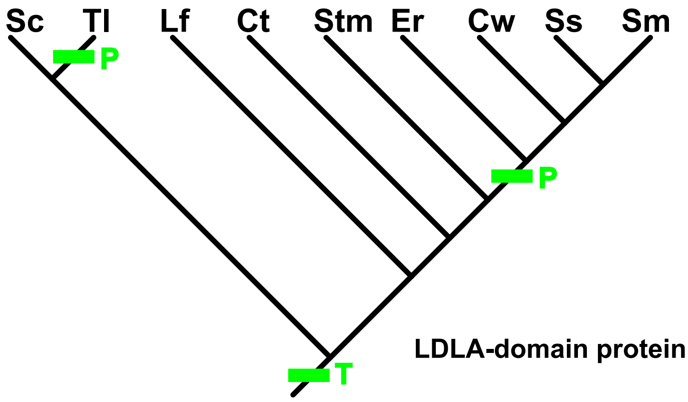

LDLA-domain protein

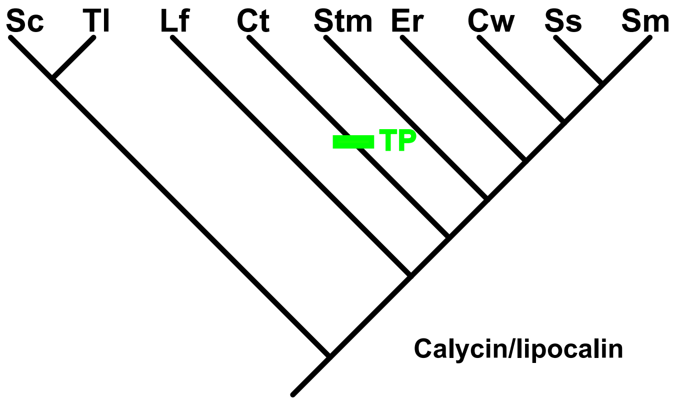

Calycin/lipocalin

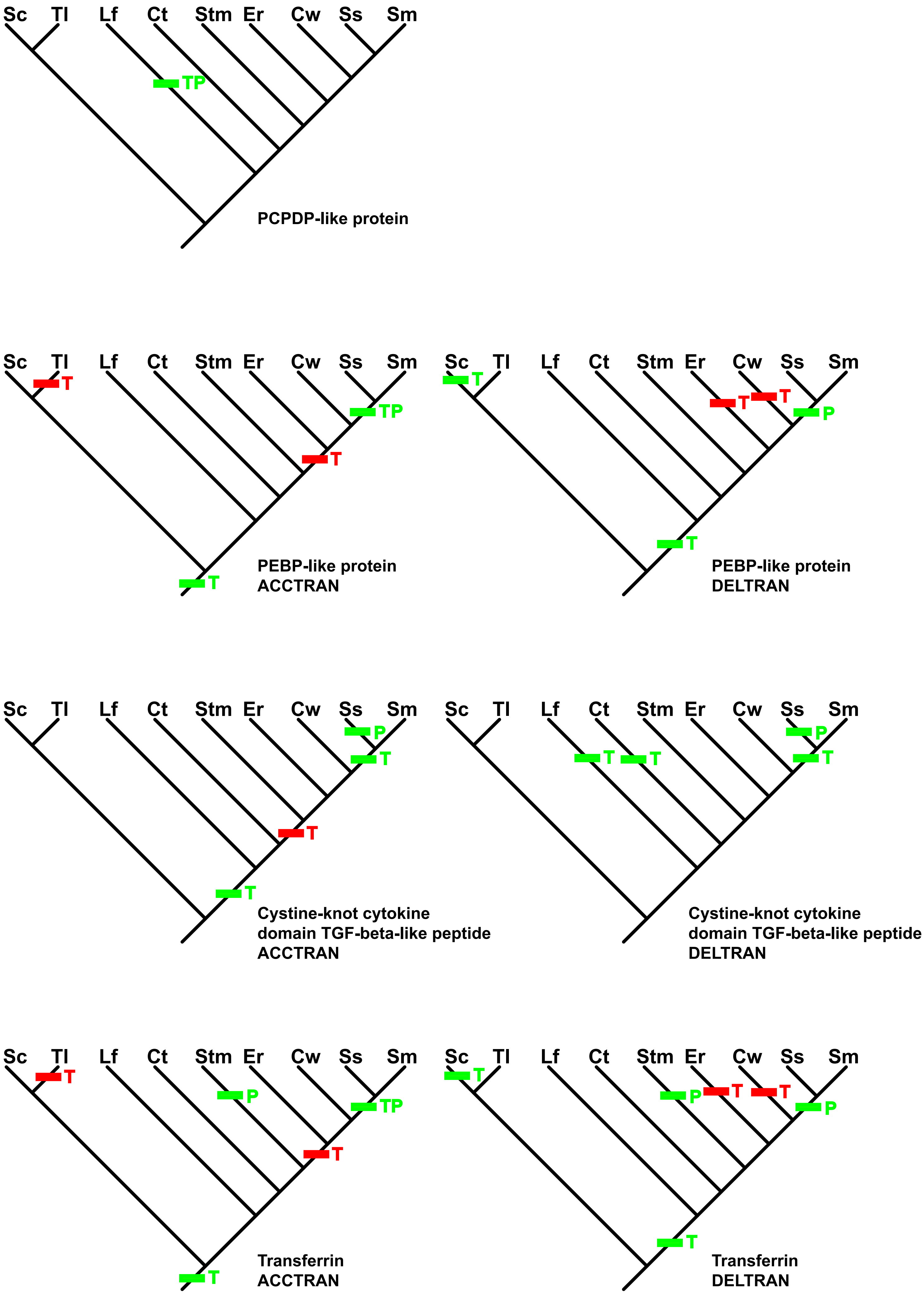
Sc Tl Lf Ct Stm Er Cw Ss Sm
TP
PCPDP-like protein
T
T
TP
T
PEBP-like protein
ACCTRAN
T
T
T
P
T
PEBP-like protein
DELTRAN
P
T
T
T
Cystine-knot cytokine
domain TGF-beta-like peptide
ACCTRAN
T
T
P
T
Cystine-knot cytokine
domain TGF-beta-like peptide
DELTRAN
T
P
TP
T
T
Transferrin
ACCTRAN
T
P
T
T
P
T
Transferrin
DELTRAN

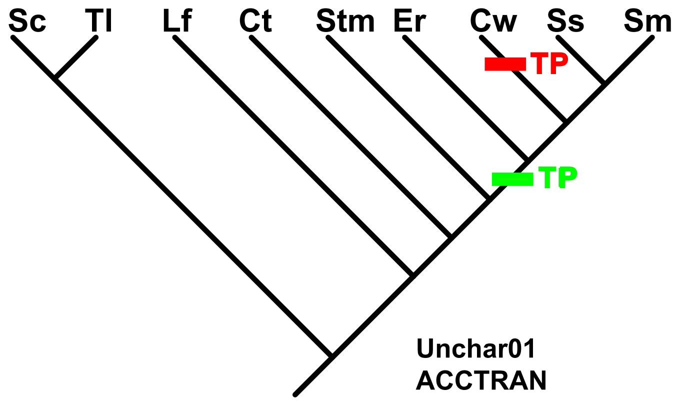
Sc Tl Lf Ct Stm Er Cw Ss Sm
TP
TP
Unchar01
ACCTRAN

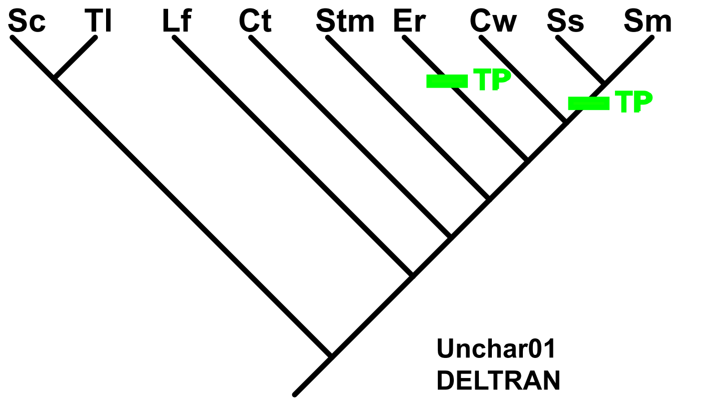
Sc Tl Lf Ct Stm Er Cw Ss Sm
TP
TP
Unchar01
DELTRAN

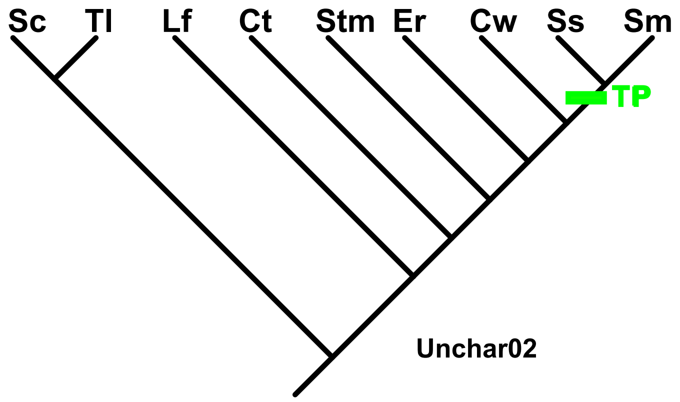
Sc Tl Lf Ct Stm Er Cw Ss Sm
TP
Unchar02

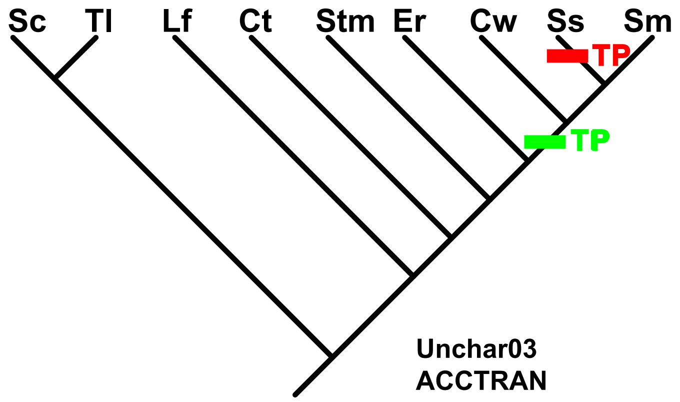
Sc Tl Lf Ct Stm Er Cw Ss Sm
TP
TP
Unchar03
ACCTRAN

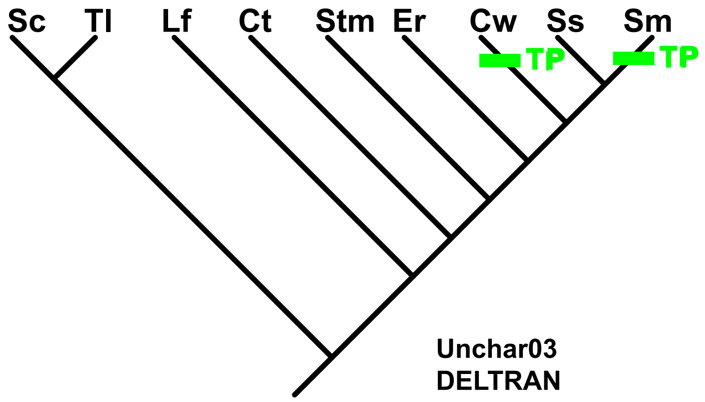
Sc Tl Lf Ct Stm Er Cw Ss Sm
TP
TP
Unchar03
DELTRAN

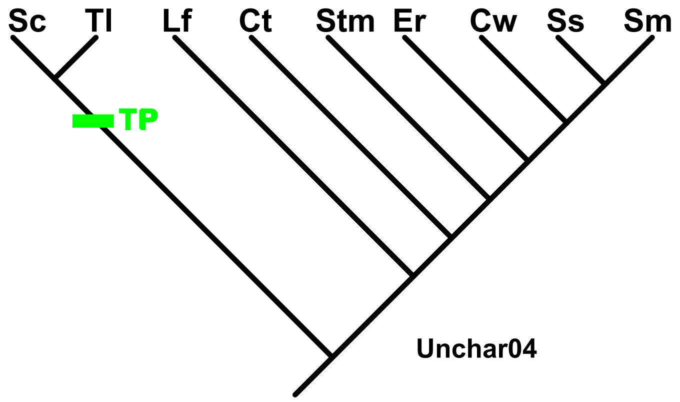
Sc Tl Lf Ct Stm Er Cw Ss Sm
TP
Unchar04

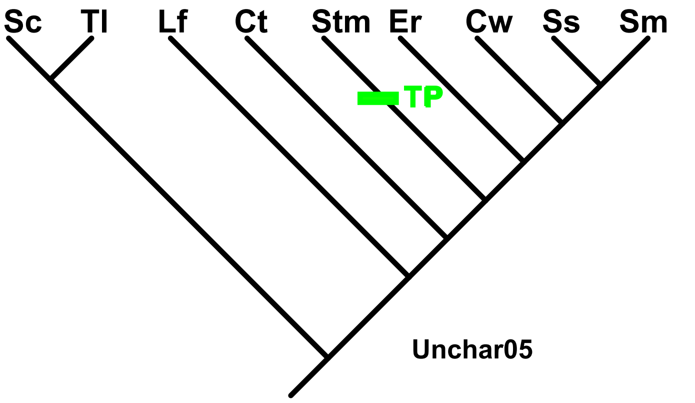
Sc
Tl
Lf
Ct
Stm
Er
Cw
Ss
Sm
TP
Unchar05

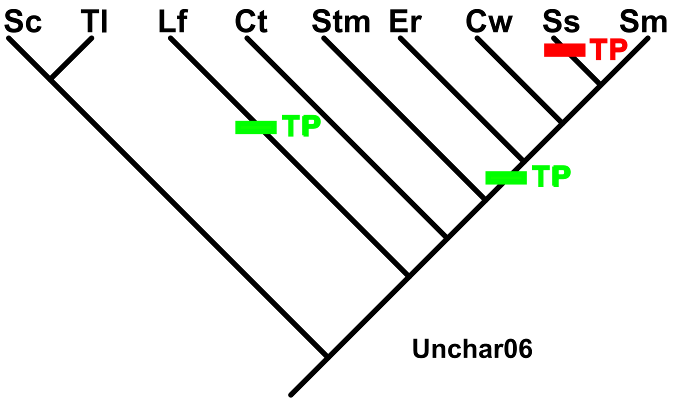
Sc
Tl
Lf
Ct
Stm
Er
Cw
Ss
Sm
TP
TP
TP
Unchar06

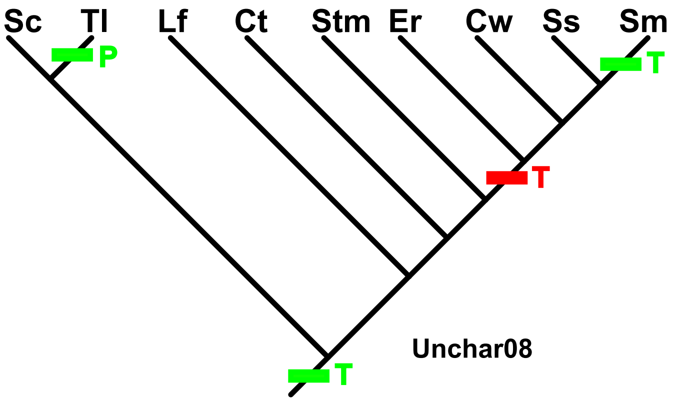
Sc
Tl
Lf
Ct
Stm
Er
Cw
Ss
Sm
P
T
T
T
Unchar08

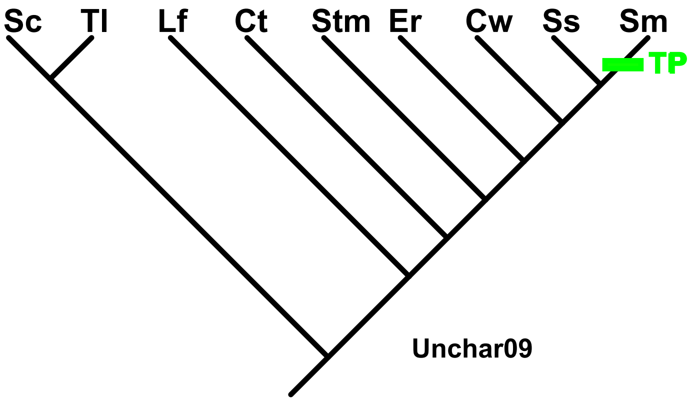
Sc
Tl
Lf
Ct
Stm
Er
Cw
Ss
Sm
TP
Unchar09

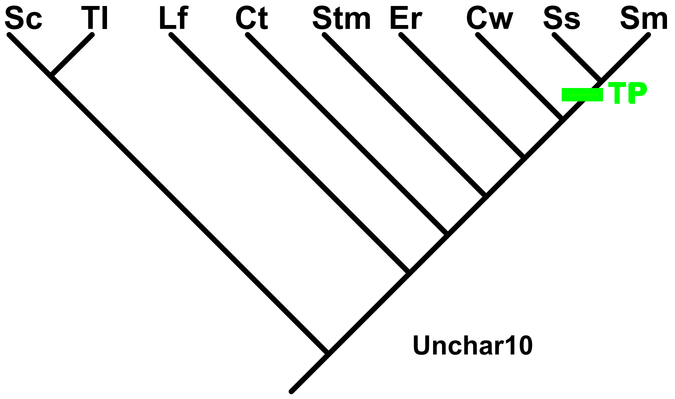

Unchar10

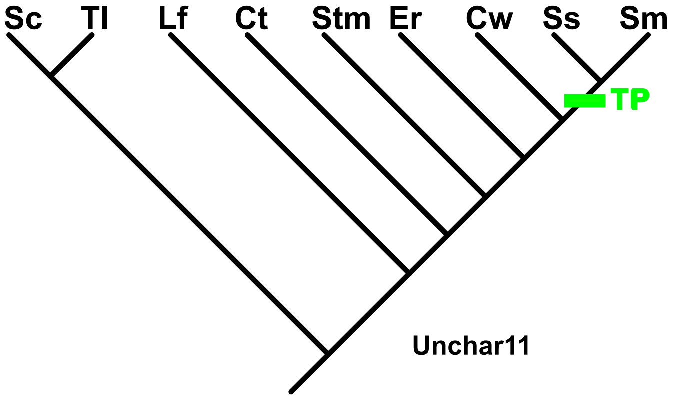

Unchar11

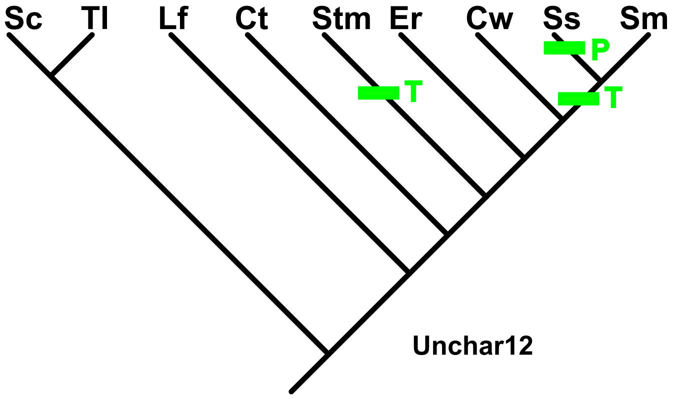

Unchar12

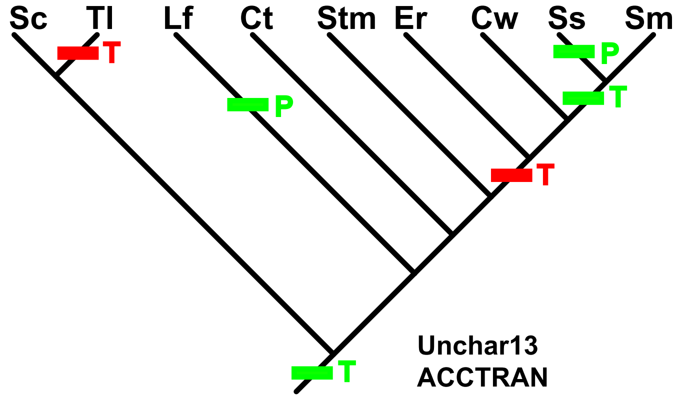

Unchar13
ACCTRAN

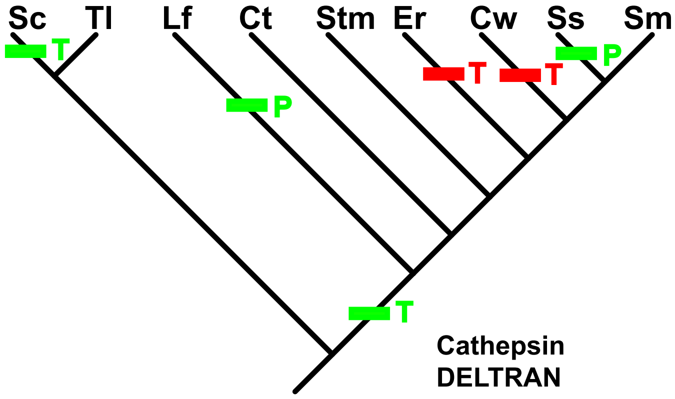

Cathepsin
DELTRAN

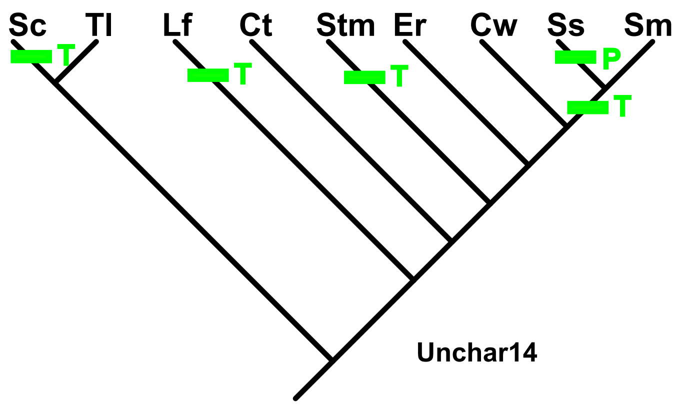
Sc
Tl
Lf
Ct
Stm
Er
Cw
Ss
Sm
T
T
T
P
T
Unchar14

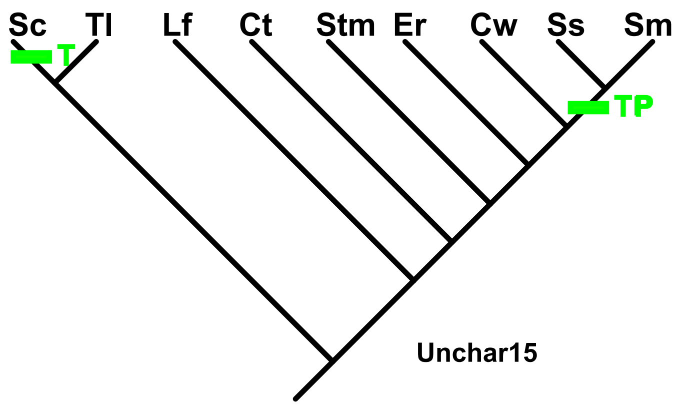
Sc
Tl
Lf
Ct
Stm
Er
Cw
Ss
Sm
T
TP
Unchar15

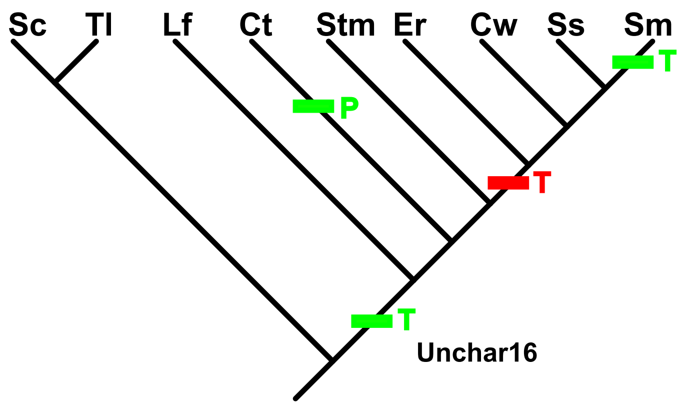
Sc
Tl
Lf
Ct
Stm
Er
Cw
Ss
Sm
T
P
T
T
Unchar16

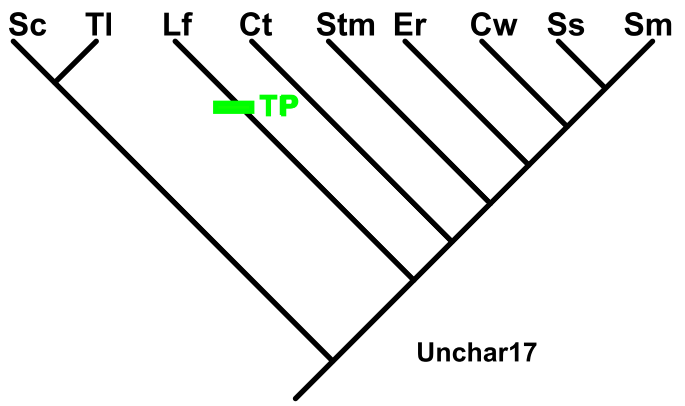
Sc
Tl
Lf
Ct
Stm
Er
Cw
Ss
Sm
TP
Unchar17

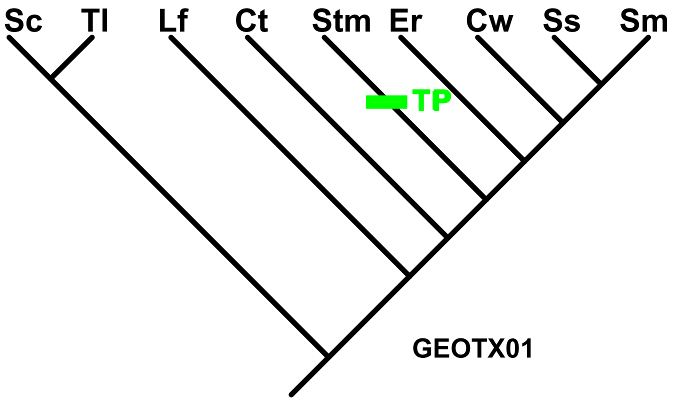

GEOTX01

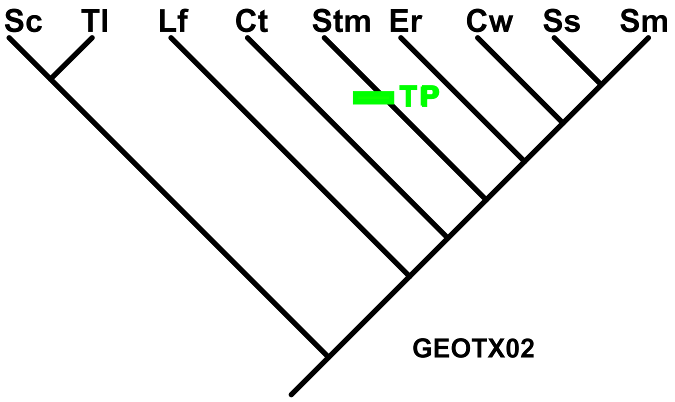

GEOTX02

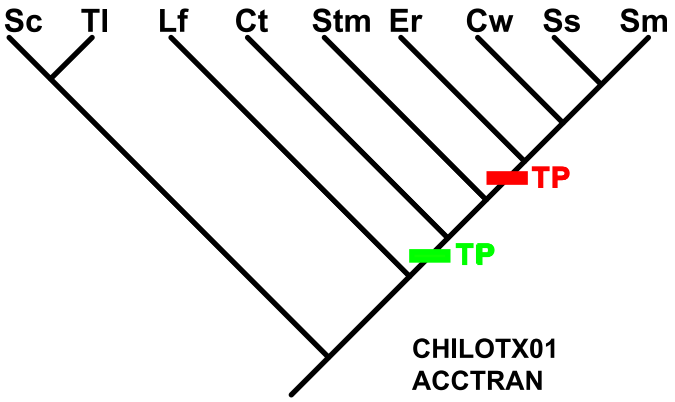

CHILOTX01
ACCTRAN

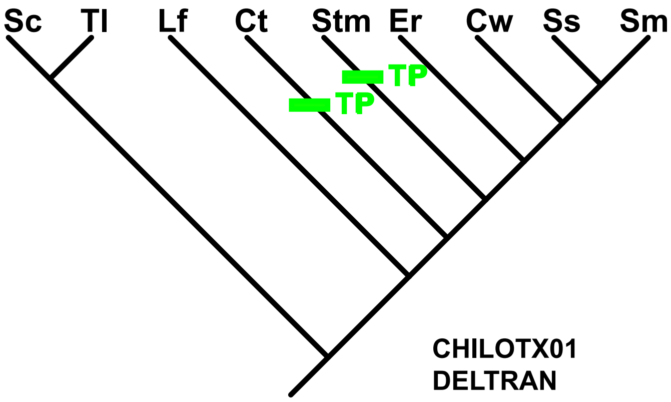

CHILOTX01
DELTRAN

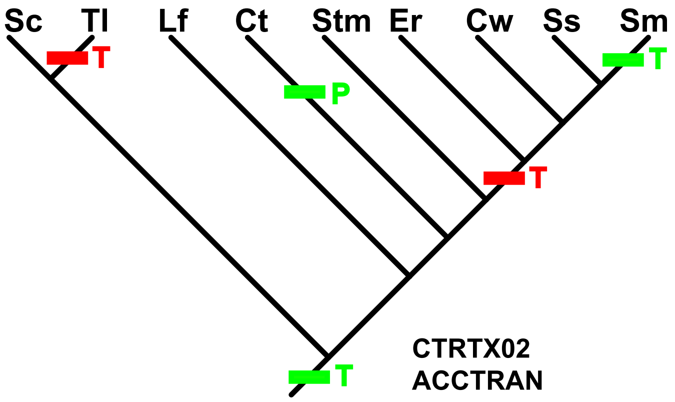

CTRTX02
ACCTRAN

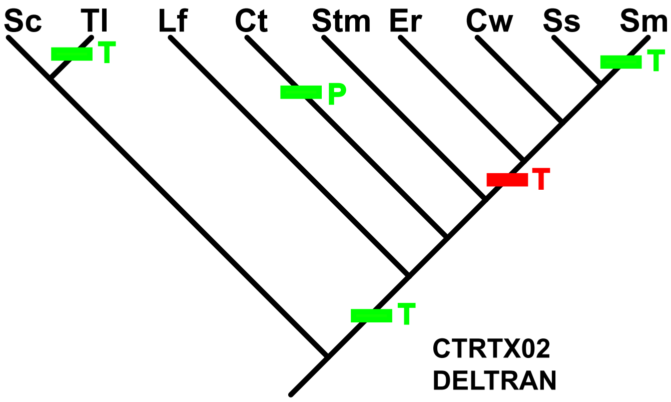

CTRTX02
DELTRAN

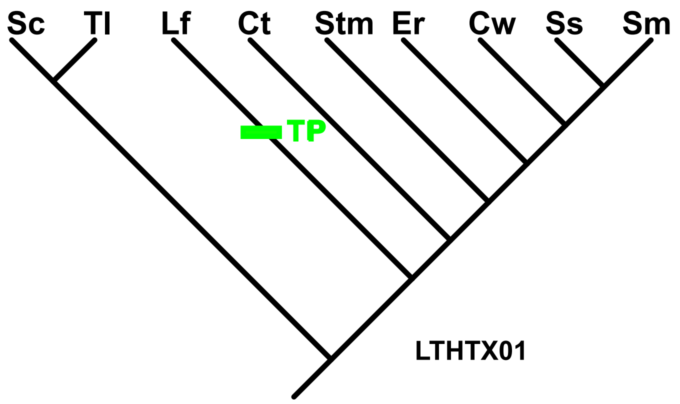

LTHTX01

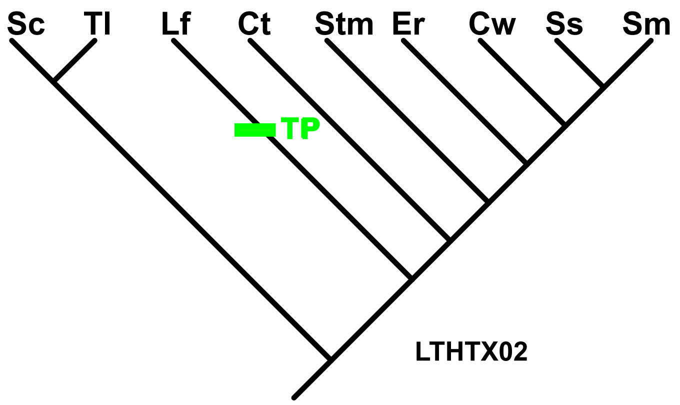

LTHTX02

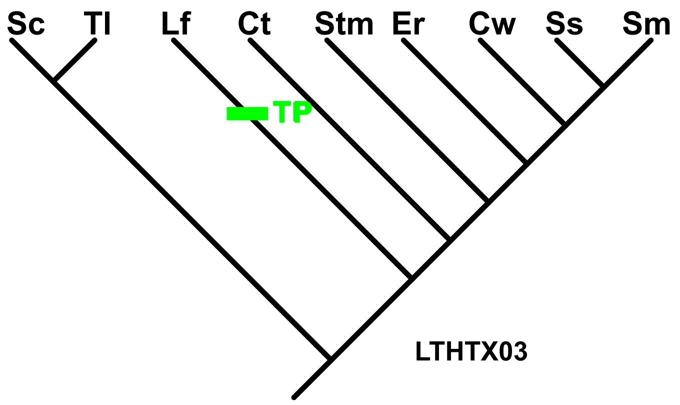

LTHTX03

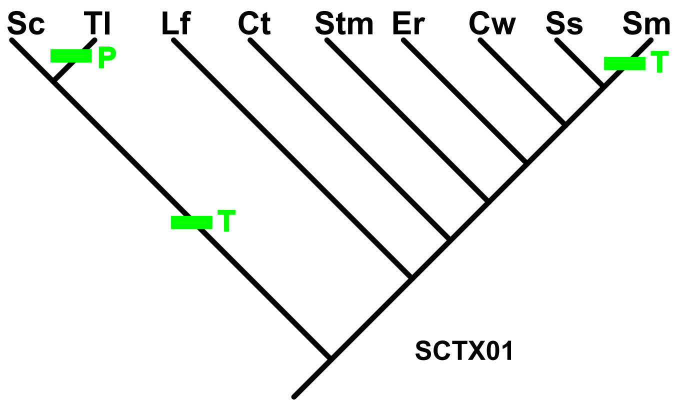

SCTX01

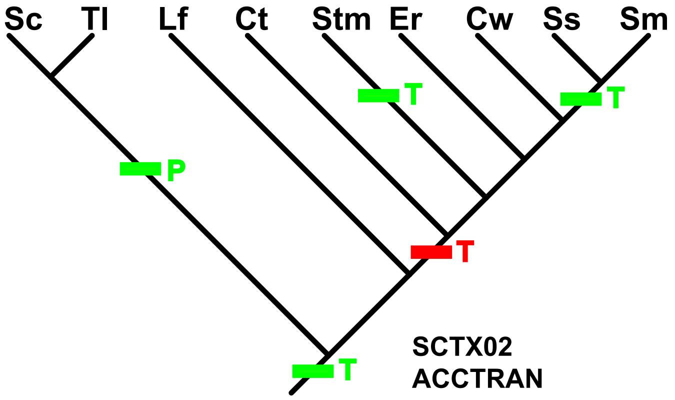

SCTX02
ACCTRAN

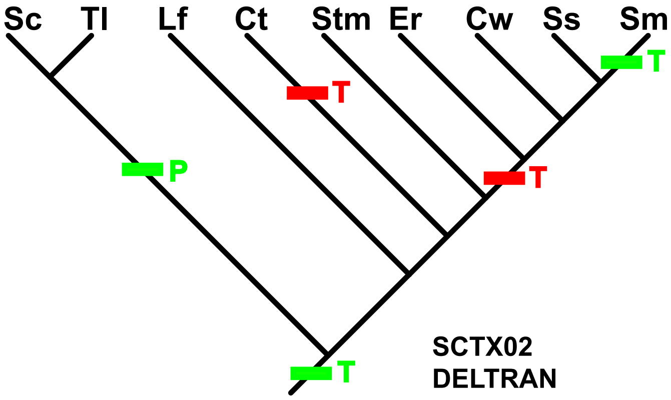

SCTX02
DELTRAN

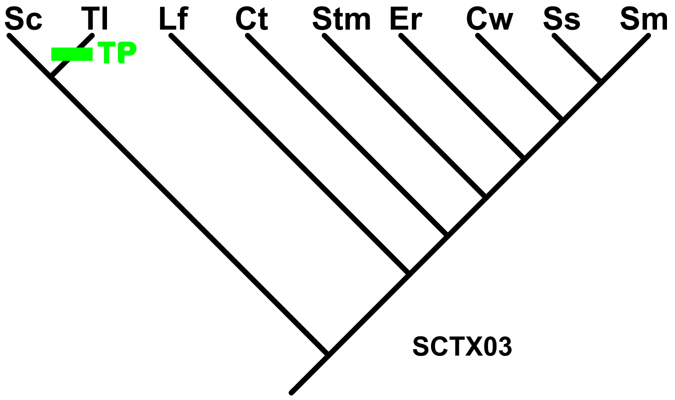

SCTX03

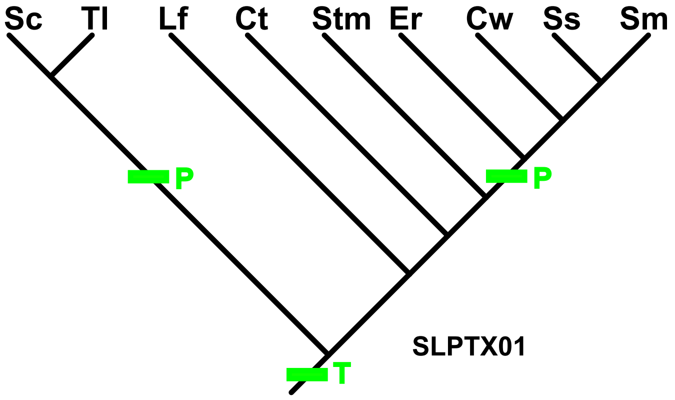

SLPTX01

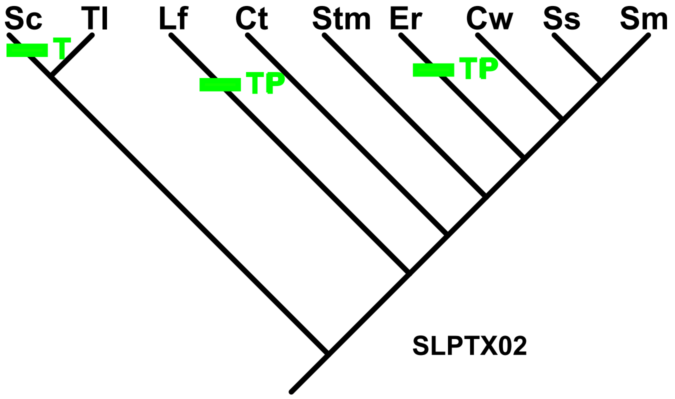

SLPTX02

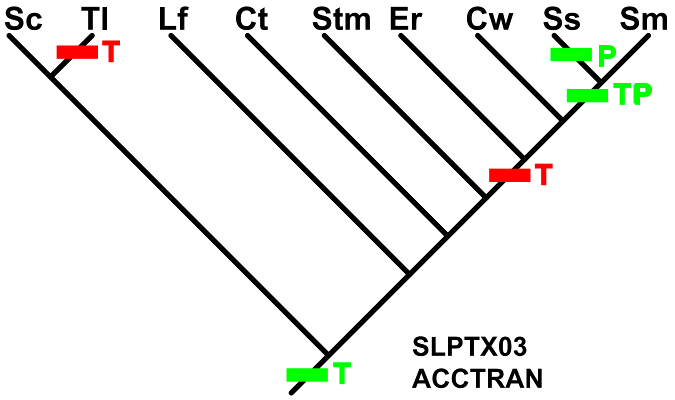

SLPTX03
ACCTRAN

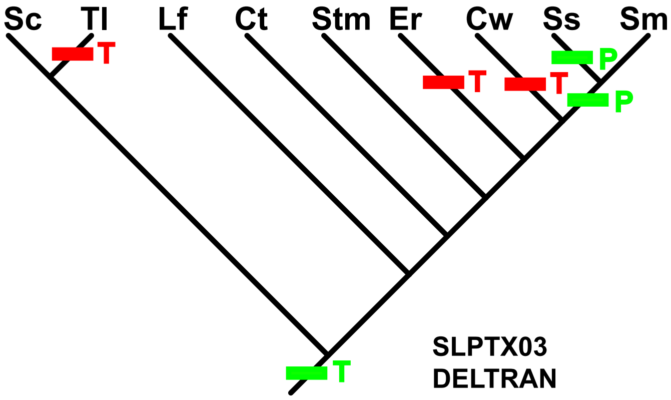

SLPTX03
DELTRAN

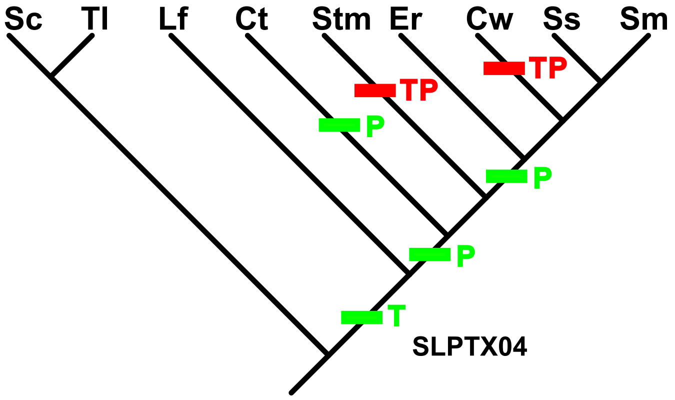

SLPTX04

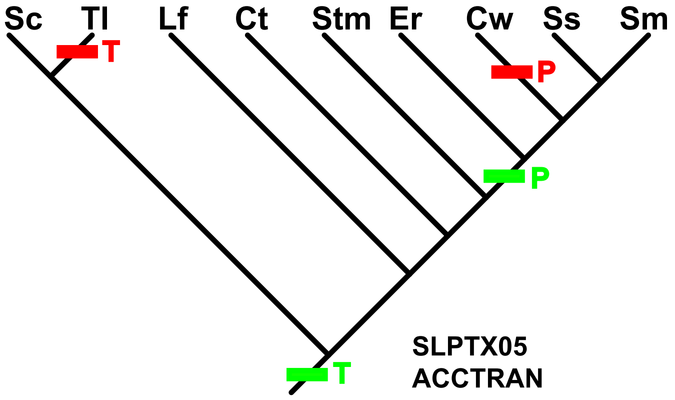

SLPTX05
ACCTRAN

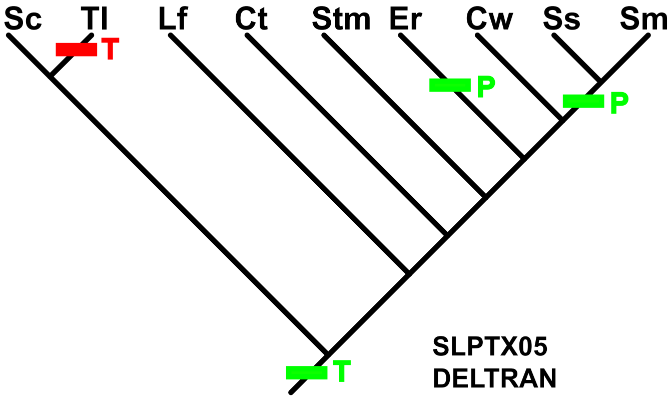

SLPTX05
DELTRAN

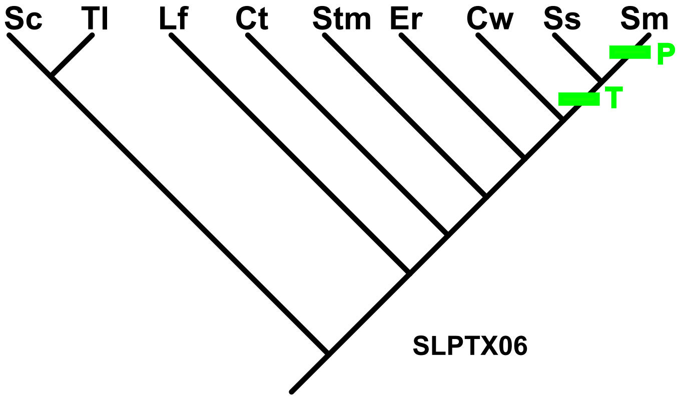

SLPTX06

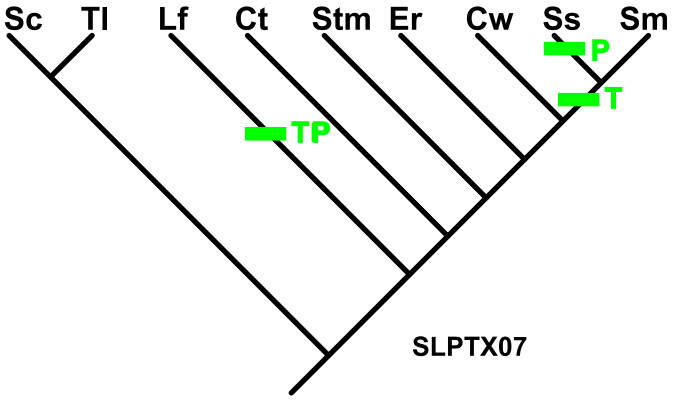
Sc Tl Lf Ct Stm Er Cw Ss Sm
P
T
TP
SLPTX07

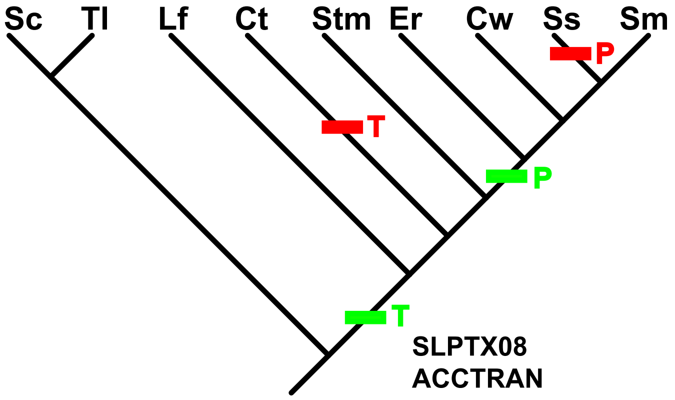
Sc Tl Lf Ct Stm Er Cw Ss Sm
P
T
P
T
SLPTX08
ACCTRAN

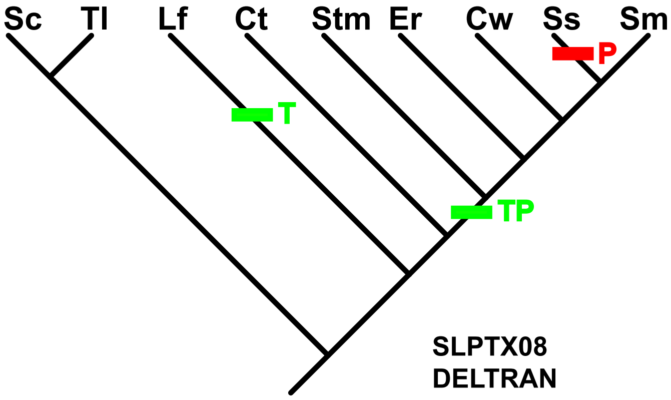
Sc Tl Lf Ct Stm Er Cw Ss Sm
P
T
TP
SLPTX08
DELTRAN

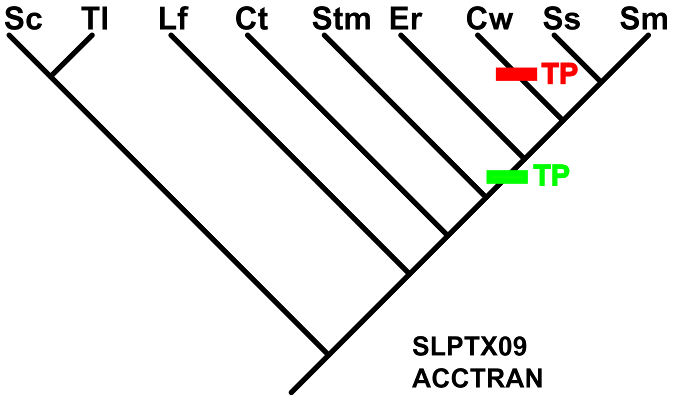
Sc Tl Lf Ct Stm Er Cw Ss Sm
TP
TP
SLPTX09
ACCTRAN

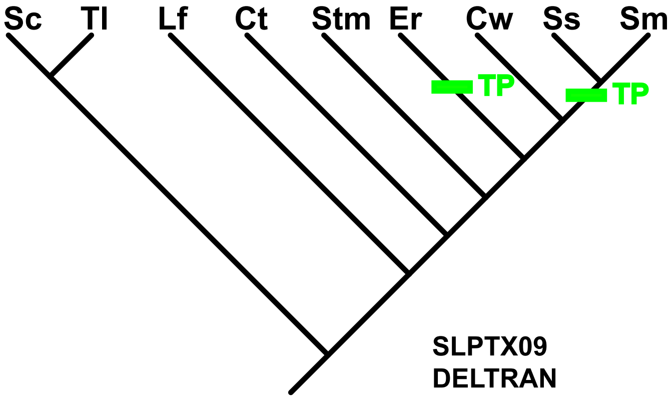
Sc Tl Lf Ct Stm Er Cw Ss Sm
TP
TP
SLPTX09
DELTRAN

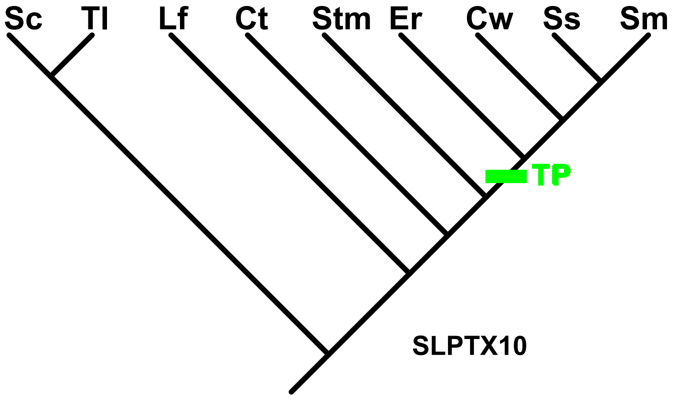
Sc Tl Lf Ct Stm Er Cw Ss Sm
TP
SLPTX10

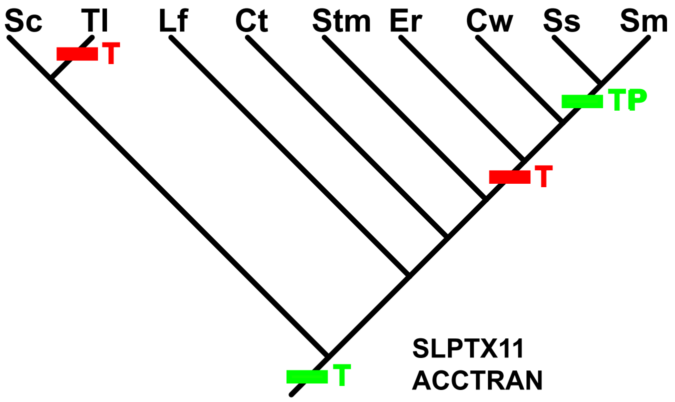

SLPTX11
ACCTRAN

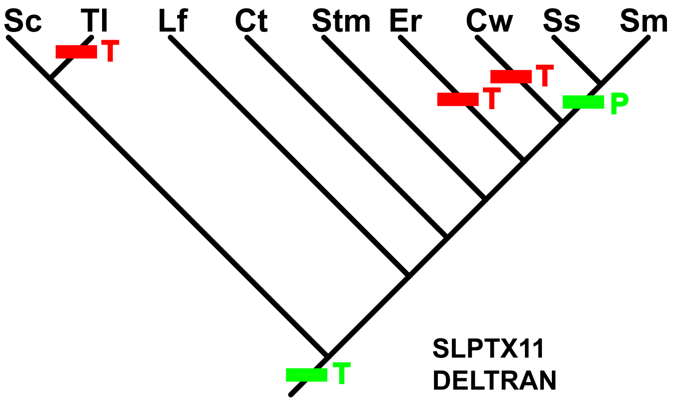

SLPTX11
DELTRAN

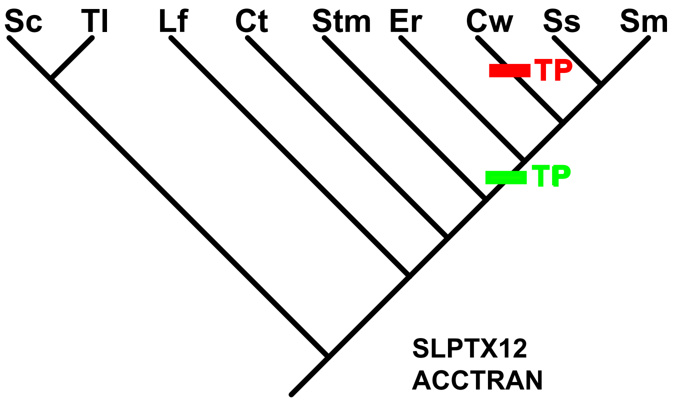

SLPTX12
ACCTRAN

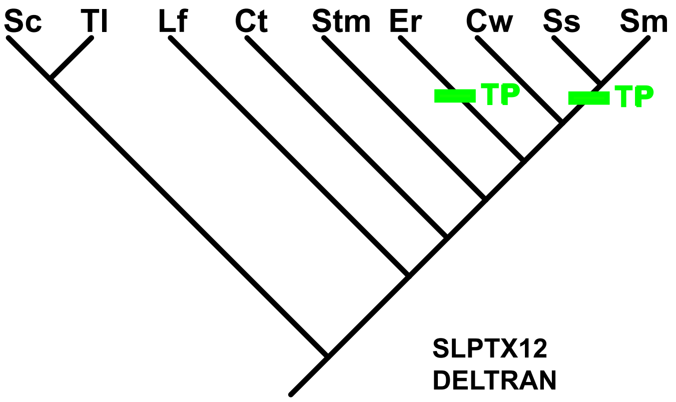

SLPTX12
DELTRAN

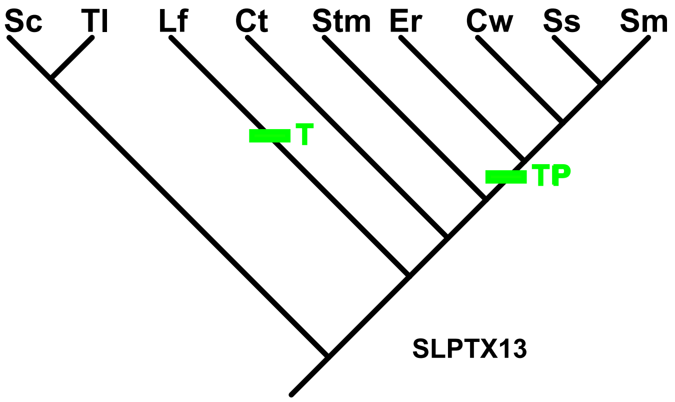

SLPTX13

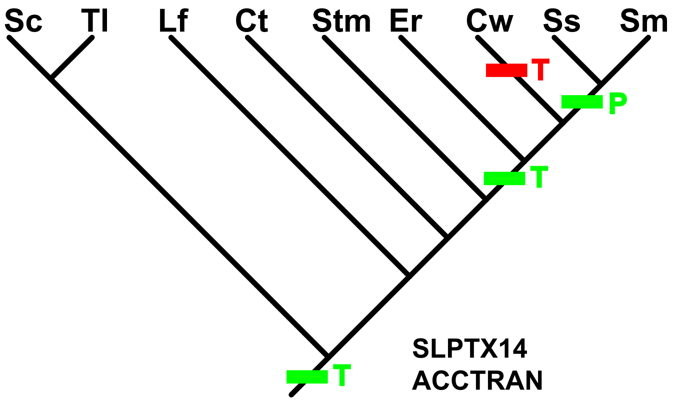

SLPTX14
ACCTRAN

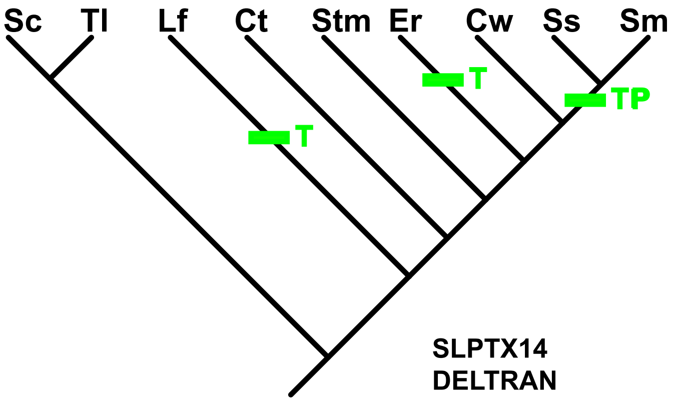

SLPTX14
DELTRAN

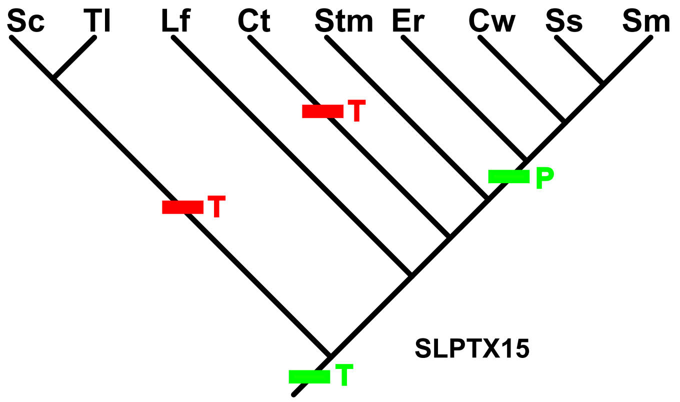
Sc
Tl
Lf
Ct
Stm
Er
Cw
Ss
Sm
T
T
P
T
SLPTX15

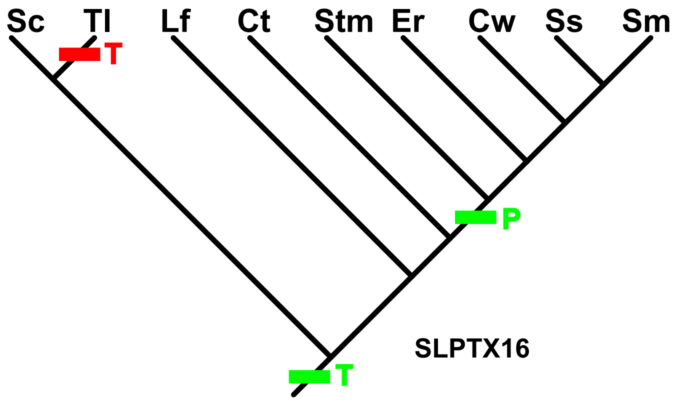
Sc
Tl
Lf
Ct
Stm
Er
Cw
Ss
Sm
T
P
T
SLPTX16

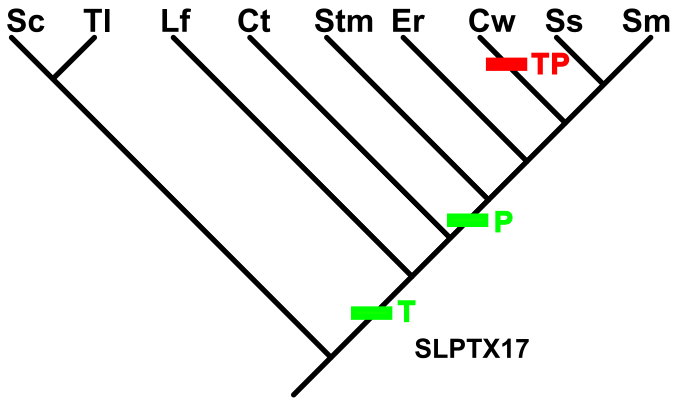
Sc
Tl
Lf
Ct
Stm
Er
Cw
Ss
Sm
TP
P
T
SLPTX17

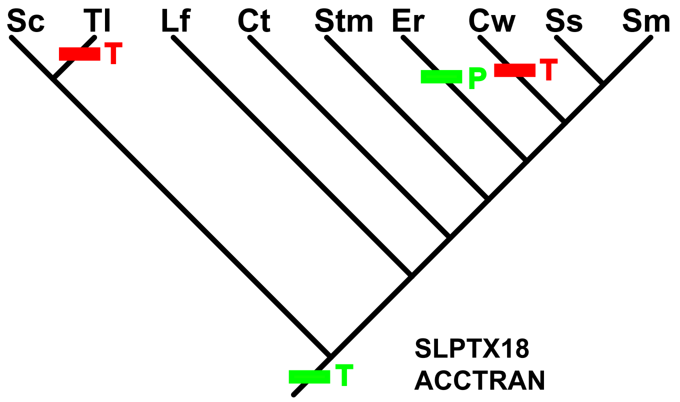
Sc
Tl
Lf
Ct
Stm
Er
Cw
Ss
Sm
T
P
T
T
SLPTX18
ACCTRAN

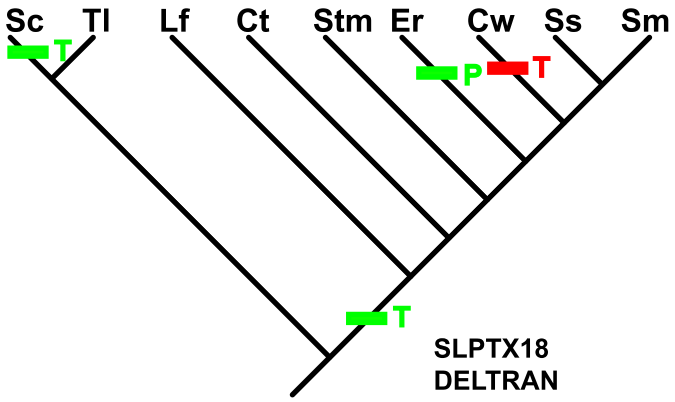
Sc
Tl
Lf
Ct
Stm
Er
Cw
Ss
Sm
T
P
T
T
SLPTX18
DELTRAN

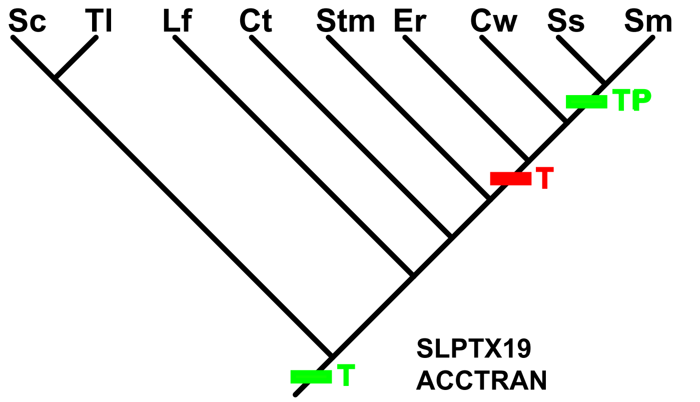
Sc Tl Lf Ct Stm Er Cw Ss Sm
TP
T
T
SLPTX19
ACCTRAN

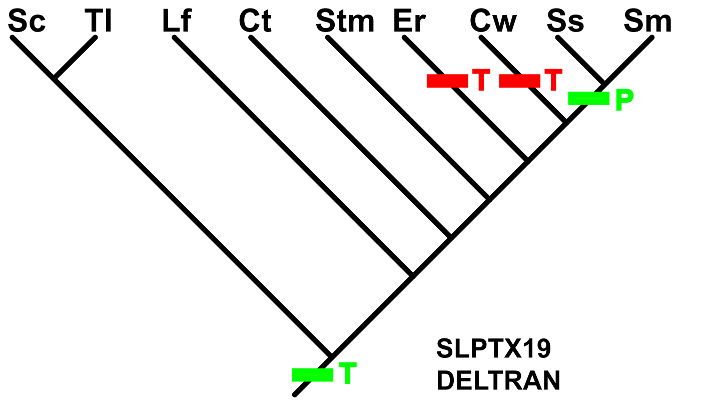
Sc Tl Lf Ct Stm Er Cw Ss Sm
T
T
P
T
SLPTX19
DELTRAN

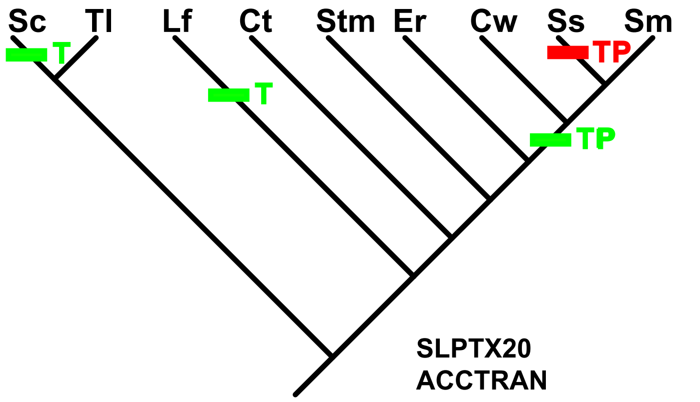
Sc Tl Lf Ct Stm Er Cw Ss Sm
T
TP
T
TP
SLPTX20
ACCTRAN

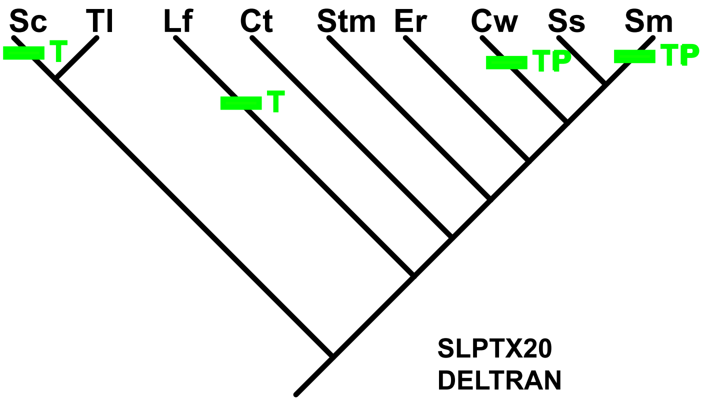
Sc Tl Lf Ct Stm Er Cw Ss Sm
T
TP
TP
T
SLPTX20
DELTRAN

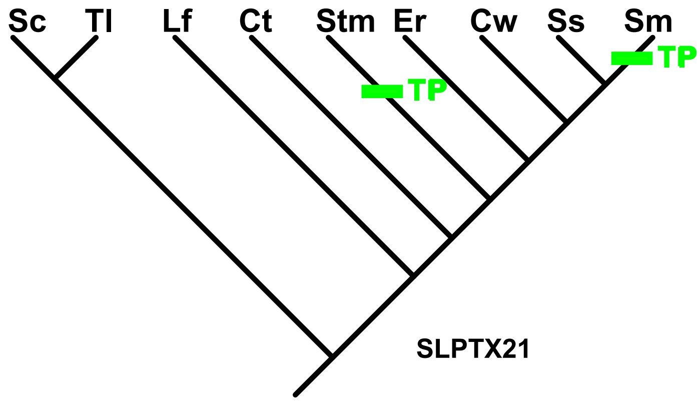
Sc Tl Lf Ct Stm Er Cw Ss Sm
TP
TP
SLPTX21

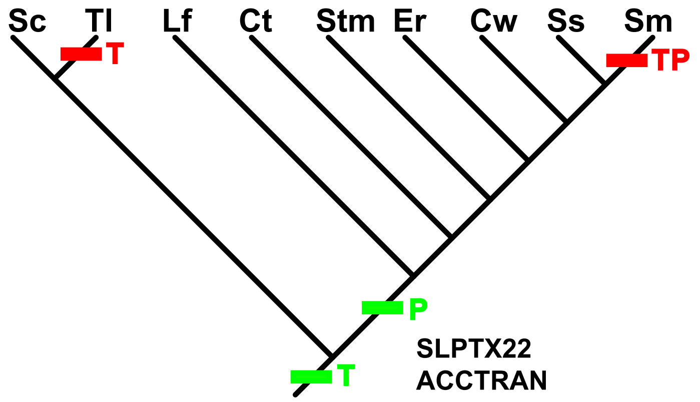
Sc Tl Lf Ct Stm Er Cw Ss Sm
T
TP
P
T
SLPTX22
ACCTRAN

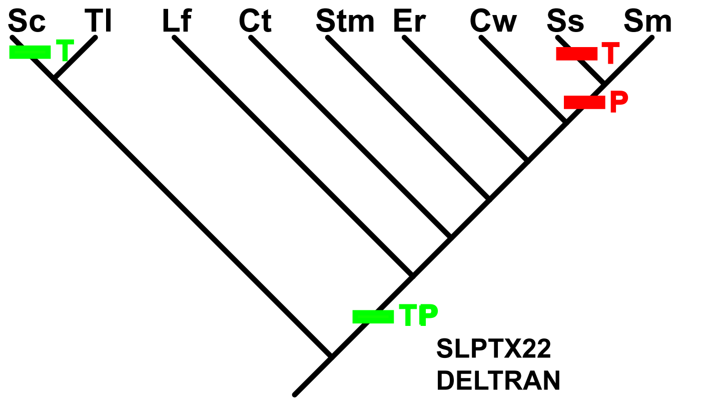
Sc Tl Lf Ct Stm Er Cw Ss Sm
T
T
P
TP
SLPTX22
DELTRAN

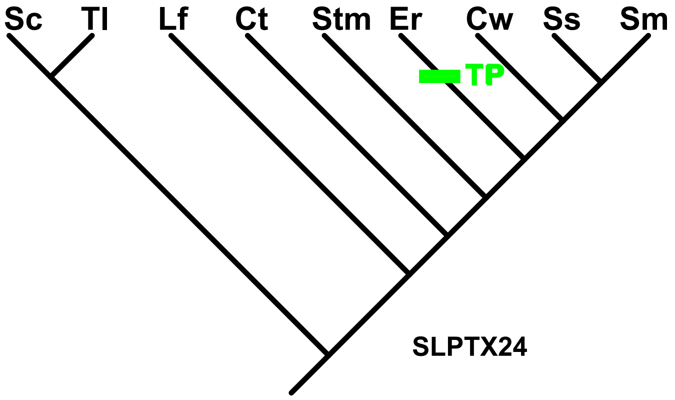

SLPTX24

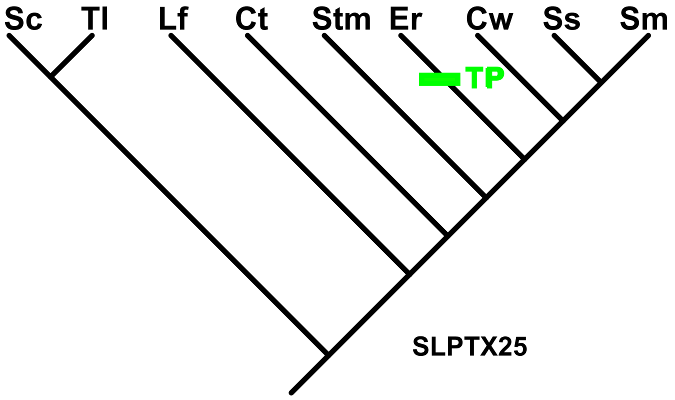

SLPTX25

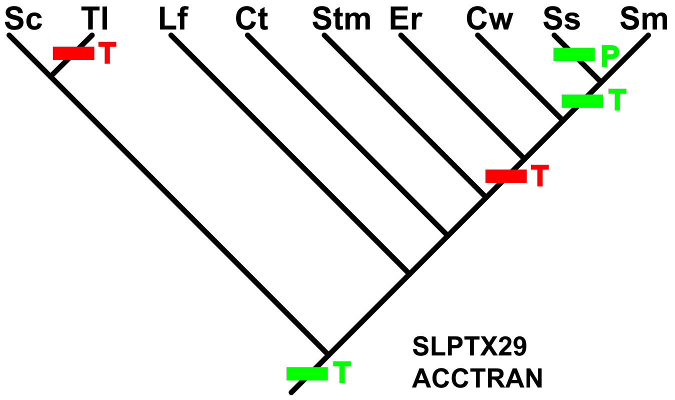

SLPTX29
ACCTRAN

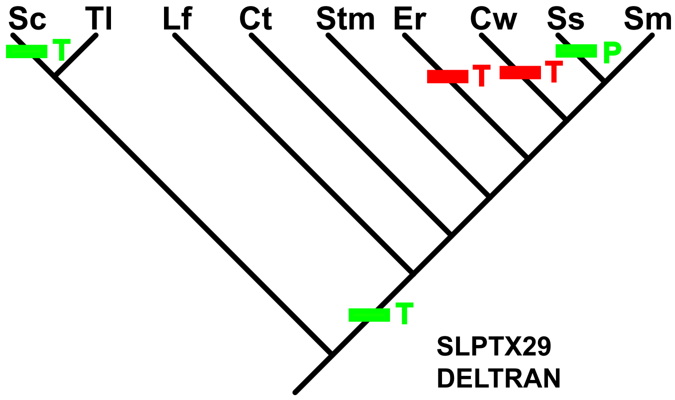

SLPTX29
DELTRAN

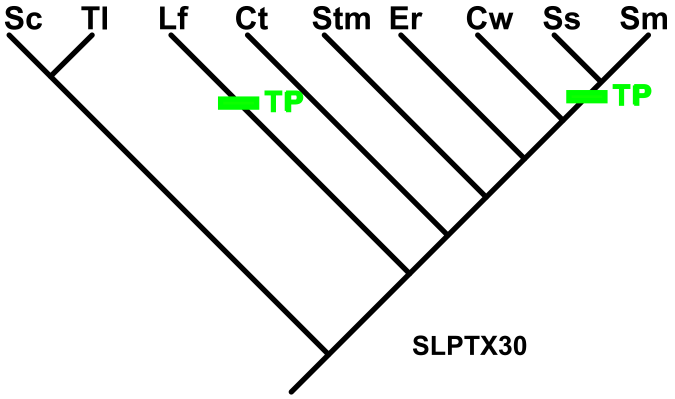

SLPTX30

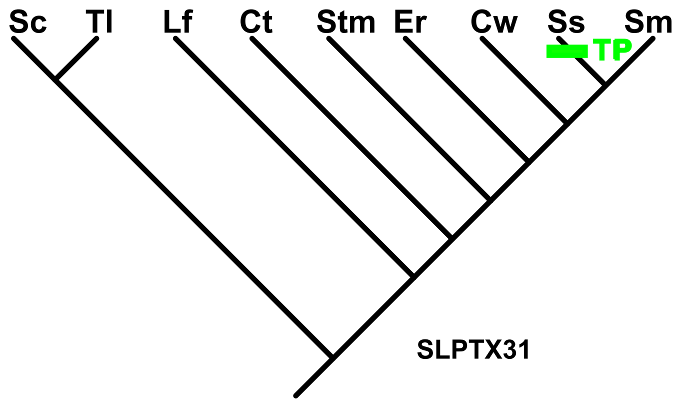
Sc
Tl
Lf
Ct
Stm
Er
Cw
Ss
Sm
TP
SLPTX31
