# Supplementary material for: Parallel Evolution of Complex Centipede Venoms Revealed by Comparative Proteotranscriptomic Analyses
Source: Mol Biol Evol. 2019 Aug 8;36(12):2748–63. doi: 10.1093/molbev/msz181 (PMC6878950; doi:10.1093/molbev/msz181)
Supplement: msz181_Supplementary_Data [file msz181_supplementary_data.zip › Supplementary_Material_S9.pdf]

Supplementary Materal S10: Details and NCBI sequence read archive accession numbers for publicly available myriapod transcriptomes used as additional samples and outgroups, along with the sequenced tissue type and the number of assembled contigs.

CLASS	SPECIES	TISSUE	ACCESSION	CONTIGS
Diplopoda	*Abacion magnum*	Whole body	SRR945438	37008
Diplopoda	*Petaserpes* sp.	Whole body	SRR945431	28529
Diplopoda	*Brachycybe lecontii*	Whole body	SRR945430	44049
Diplopoda	*Cambala annulata*	Whole body	SRR945440	29150
Diplopoda	*Glomeridesmus* sp.	Whole body	SRR941771	46226
Diplopoda	*Prostemmiulus* sp.	Whole body	SRR945439	21872
Diplopoda	*Cleidogona* sp.	Whole body	SRR945437	40791
Diplopoda	*Pseudopolydesmus* sp.	Whole body	SRR945436	45440
Chilopoda	*Lithobius* sp.	Whole body	SRR945441	83643
Chilopoda	*Craterostigmus tasmanianus*	Trunk	SRR1157986	352640
Chilopoda	*Cryptops hortensis*	Trunk	SRR1153457	152987
Chilopoda	*Himantarium gabrielis*	Trunk	SRR1159787	56139
Chilopoda	*Lithobius forficatus*	Trunk	SRR1159752	170803
Chilopoda	*Scutigera coleoptrata*	Anterior whole body	SRR1158078	336940
Chilopoda	*Scolopendra subspinipes mutilans*	Whole body (infected)	SRR867647	63865
Chilopoda	*Scolopendra subspinipes mutilans*	Whole body (normal)	SRR867646	31836
